# Cladobotric Acids: Metabolites from Cultures of *Cladobotryum* sp., Semisynthetic Analogues and Antibacterial Activity

**DOI:** 10.1021/acs.jnatprod.1c01063

**Published:** 2022-02-16

**Authors:** Trong-Tuan Dao, Katherine Williams, Kate M. J. de Mattos-Shipley, Zhongshu Song, Yuiko Takebayashi, Thomas J. Simpson, James Spencer, Andrew M. Bailey, Christine L. Willis

**Affiliations:** † School of Chemistry, University of Bristol, Cantock’s Close, Bristol, BS8 1TS, U.K.; ‡ School of Biological Sciences, Life Sciences Building, University of Bristol, 24 Tyndall Avenue, Bristol, BS8 1TQ, U.K.; § School of Cellular and Molecular Medicine, University of Bristol, University Walk, Bristol, BS8 1TD, U.K.

## Abstract

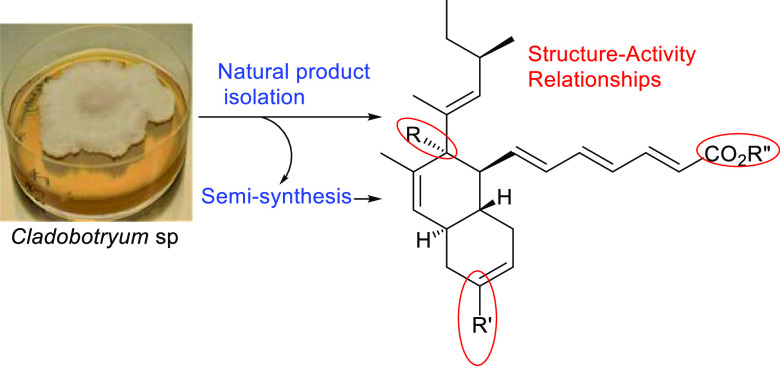

Three new polyketide-derived natural products, cladobotric acids G–I (**1**–**3**), and six known metabolites (**4**, **5**, **8**–**11**) were isolated from fermentation of the fungus *Cladobotryum* sp. grown on rice. Their structures were elucidated by extensive spectroscopic methods. Two metabolites, cladobotric acid A (**4**) and pyrenulic acid A (**10**), were converted to a series of new products (**12**–**20**) by semisynthesis. The antibacterial activities of all these compounds were investigated against the Gram-positive pathogen *Staphylococcus aureus* including methicillin-susceptible (MSSA), methicillin-resistant and vancomycin-intermediate (MRSA/VISA), and heterogeneous vancomycin-intermediate (hVISA) strains. Results of these antibacterial assays revealed structural features of the unsaturated decalins important for biological activity.

With the increase of antibiotic-resistant bacteria worldwide and the lack of new antibiotics,^1,2^ there is a continuing need for the discovery and development of effective antibacterial agents. The majority of commonly used antibiotics in both the clinic and agriculture either are natural products or are analogues or derivatives inspired by natural product leads.^2^ With significant advances in genome mining there are excellent prospects for discovering new compounds with antibiotic activity, potentially with novel modes of action.^3−7^ In the course of screening for antibacterial natural products, we turned our attention to the fungal strain *Cladobotryum* sp. CANU E1042. *Cladobotryum* fungi are known to be the causal agents of “cobweb disease” in agriculture^8^ and have been reported to produce a number of bioactive secondary metabolites, including cyclodepsipeptides,^9^ cladobotric acids,^10^ tricyclic derivatives,^11^ substituted pyridinediones,^12^ cladobotrins,^13^ furopyridines,^14^ and azatricyclic phosphate esters.^15^ In 2006, Munro and co-workers reported the isolation of six unsaturated decalin-type natural products named cladobotric acids A–F (**4**–**9**) from the fermentation broth of a New Zealand *Cladobotryum* species.^10^ The absolute configuration of cladobotric acid A (**4**) was determined using X-ray crystallography of the *p*-bromo ester derivative. The results of feeding studies with [^13^C]-labeled precursors were in accord with the proposed polyketide origin of the cladobotric acids. More recently two compounds closely related to the cladobotric acids, pyrenulic acids A and B (**10** and **11**, respectively), were isolated from a spore-derived mycobiont of a crustose *Pyrenula* sp. lichen collected in Vietnam, which showed cytotoxic effects against HCT116 human colon carcinoma.^16^

**Chart 1:**
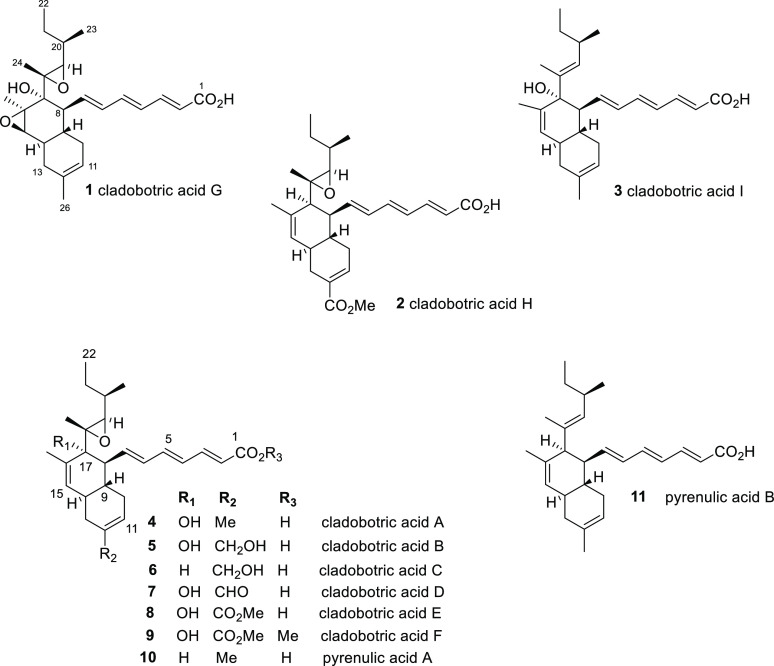


Herein we report the isolation and structure elucidation of three new cladobotric acids (**1**–**3**) from cultures of *Cladobotryum* sp. CANU E1042,^10^ which are now named cladobotric acids G–I, along with six known natural products (**4**, **5**, **8**–**11**). Structural modifications of the major metabolites cladobotric acid A (**4**) and pyrenulic acid A (**10**) via either reduction or treatment with acid gave nine new unsaturated decalins. The structure–activity relationships (SAR) within this family were investigated by establishing antibacterial activity against the Gram-positive bacterial pathogen *Staphylococcus aureus*.

## Results and Discussion

### Isolation and Structure Elucidation

*Cladobotryum* sp. was grown on rice. After 14 days the growth medium was extracted with EtOAc. Purification of the metabolites by successive chromatographic procedures (silica gel, Sephadex LH-20, RP-18, and HPLC) yielded the six known polyketide-derived natural products (**4**, **5**, **8**–**11**) as well as three new related compounds, **1**–**3**.

Compound **1** was obtained as a pale yellow solid (4.5 mg) with the molecular formula C_26_H_36_O_5_ as determined from the sodium adduct ion [M + Na]^+^ peak at *m*/*z* 451.2470 (calcd for C_26_H_36_O_5_Na, 451.2460) in the HRMS spectrum. Its UV spectrum showed an intense absorption band, λ_max_ (log ε), at 302 nm. Its IR spectrum revealed the presence of hydroxy and carbonyl groups (3407 and 1694 cm^–1^, respectively). The structure was deduced by detailed analysis of the 1D and 2D NMR data (Table 1). The ^1^H NMR spectrum of **1** displayed signals for seven olefinic protons, including three *E*-double bonds, six methine protons including two oxygenated methines, three pairs of methylene protons, and five methyl groups. The ^13^C NMR data (Table 1) revealed 26 carbon atoms, including four double bonds, of which one was trisubstituted with a signal for the quaternary carbon at δ_C_ 134.0 (C-12), three oxygenated quaternary sp^3^ carbons, δ_C_ 73.1 (C-17), 64.5 (C-18), and 62.4 (C-16), and a signal at δ_C_ 169.7 (C-1) assigned to a carboxylic acid. The full assignment was achieved using 2D (COSY, HSQC, and HMBC) NMR experiments, which revealed the partial structures of **1** as a highly substituted, unsaturated decalin with a trienoic acid side-chain at C-8, similar to that of cladobotric acid A (**4**).^10^ The major differences in the ^13^C NMR spectra of the two metabolites were the signals at δ_C_ 134.2 (C-15) and 124.3 (C-16) assigned to the 15,16-alkene in cladobotric acid A (**4**) versus those at δ_C_ 64.2 and 62.4 in the new product **1**, which when taken together with the MS data, were in accord with a 15,16-epoxide. HMBC correlations of 25-H_3_/C-15, C-16, and C-17 and 15-H/C-9, C-13, C-14, C-16, and C-25 confirmed the presence of the 15,16-epoxide. The relative configuration of the 15,16-oxirane ring in **1** was deduced from ^1^H NMR, in which 15-H appeared as a singlet (δ_H_ 2.88) in the ^1^H NMR and there were NOE correlations between 8-H/14-H, 15-H/25-H_3_, and 19-H/25-H_3_ in the 2D NOESY spectrum (Table 1 and Supporting Information Figure S11). All previously reported cladobotric acids have a negative optical rotation, and therefore the absolute configuration of **1** was assigned on the basis of its similar negative value ([α]_D_ −70.2 (*c* 0.1, CHCl_3_)). This new metabolite is now named cladobotric acid G (**1**).

**Table 1:** ^1^H (500 MHz) and ^13^C (125 MHz) NMR Data^a^ for **1**–**3**

	**1**		**2**		**3**	
position	δ_H_ mult. (*J* in Hz)	δ_C_	δ_H_ mult. (*J* in Hz)	δ_C_	δ_H_ mult. (*J* in Hz)	δ_C_
1		169.7		169.9		171.6
2	5.65 d (15.5)	121.0	5.83 d (15.5)	118.9	5.88 d (15.5)	119.3
3	7.15 dd (15.5, 11.5)	146.5	7.36 dd (15.5, 11.5)	147.1	7.36 dd (15.5, 11.5)	146.8
4	6.14 dd (15.0, 11.5)	130.0	6.25 dd (15.0, 11.5)	128.4	6.29 dd (15.5, 11.0)	128.3
5	6.52 dd (15.0, 11.0)	140.6	6.71 dd (15.0, 11.0)	141.9	6.35 dd (15.0, 11.0)	141.3
6	6.09 dd (14.8, 11.0)	134.9	6.15 dd (14.8, 11.0)	130.6	6.20 dd (15.0, 11.0)	131.9
7	5.88 dd (15.0, 11.0)	135.9	6.18 dd (15.0, 11.0)	141.4	5.59 dd (15.0, 11.0)	140.0
8	2.05 m	59.7	2.35 dt (12.0, 6.5)	48.9	2.23 t (11.5)	57.9
9	1.76 m	32.8	1.73 m	35.8	1.65 m	36.6
10	1.84 m	31.7	2.27 br d (17.5)	32.4	1.81 m	30.8
	1.40 m		1.67 m		1.53 m	
11	5.33 br s	120.9	6.94 br s	139.6	5.33 br s	121.0
12		134.0		130.3		133.9
13	2.09 m	34.5	2.60 dd (14.0, 2.0)	32.0	2.02 m	37.2
	2.05 m		1.91 m		1.80 m	
14	1.86 m	39.1	1.93 m	38.1	2.05 m	38.2
15	2.88 s	64.2	5.50 br s	128.9	5.47 br s	129.1
16		62.4		132.9		135.9
17		73.1	1.80 d (6.5)	53.4		79.9
18		64.5		61.1		133.6
19	3.18 d (8.5)	63.9	2.46 d (8.5)	69.0	5.16 br d (9.5)	135.0
20	1.42 m	34.2	1.30 m	34.9	2.31 m	34.4
21	1.59 m	28.3	1.66 m	27.8	1.37 m	30.2
	1.33 m		1.29 m		1.23 m	
22	0.95 t (7.5)	11.7	0.94 t (7.5)	11.4	0.84 t (7.5)	12.1
23	1.02 d (7.0)	16.0	0.96 d (7.0)	15.6	0.93 d (6.5)	20.9
24	1.66 s	16.9	1.28 s	15.8	1.62 s	15.3
25	1.37 s	20.1	1.74 s	23.2	1.59 s	17.4
26	1.67 s	23.7		167.8	1.66 s	23.4
26-COO*CH*_*3*_			3.73 s	51.8		

a Recorded in CDCl_3_.

Compound **2** was obtained as a pale yellow solid (6.5 mg) with a molecular formula of C_27_H_36_O_5_ (HRMS *m*/*z* 463.2468 [M + Na]^+^, calcd for C_27_H_36_O_5_Na, 463.2460). The ^1^H and ^13^C NMR data of **2** (Table 1) closely resembled those of cladobotric acid C (**6**)^10^ except for the primary alcohol at C-12 (δ_H_ 4.15, δ_C_ 76.2) being replaced by a methyl ester (CO_2_CH_3_ δ_H_ 3.73, δ_C_ 51.8, 167.8). Consistent with this, the signal assigned to 11-H which appeared at δ_H_ 5.66 (br s) in **6** was now downfield at δ_H_ 6.94 (br s) in the new metabolite. These assignments were confirmed from HMBC correlations between 11-H/C-9, C-10, C-13, and C-26, between 13-H/C-11, C-12, C-14, and C-26, and between OCH_3_ of the methyl ester and C-26. Thus, compound **2** is assembled on the *trans* decalin system with the C-26 methyl ester and is now named cladobotric acid H.

Compound **3** was obtained as a pale yellow solid (4.5 mg) with a molecular formula of C_26_H_36_O_3_ (HRMS *m*/*z* 419.2544 [M + Na]^+^, calcd for C_26_H_36_O_3_Na, 419.2557). The UV, IR, and NMR spectroscopic data were again in agreement with a cladobotric acid metabolite, with data similar to those reported for pyrenulic acid B (**11**).^16^ The only difference between the two structures was the presence of the hydroxylated C-17 in **3** (δ_C_ 79.9) versus the 17-CH (δ_C_ 53.4) in pyrenulic acid B (**11**).^16^ Further characteristic NMR signals included an olefinic proton at δ_H_ 5.16 (19-H, br d, *J* 9.5 Hz) and two sp^2^ carbons at δ_C_ 133.6 (C-18) and 135.0 (C-19) in accord with a trisubstituted 18,19-alkene rather than the 18,19-epoxide characteristic of cladobotric acids A–H (Table 1). HMBC correlations from 19-H to C-18, C-20, and C-23 and from 24-H_3_ to C-17, C-18, and C-19 confirmed the presence of the 18,19-alkene in **3**. The *E* geometry was confirmed by NOE correlations between 20-H/24-H_3_. The absolute configuration was assigned on the basis of the negative value of the optical rotation [α]_D_ −60.4 (*c* 0.15, CHCl_3_), and compound **3** is thus named cladobotric acid I.

**Figure 1:**
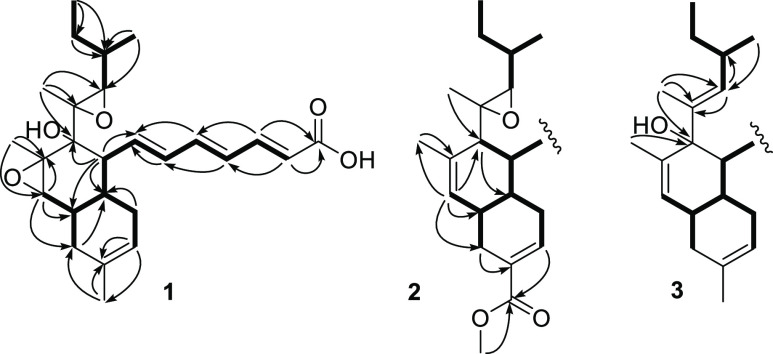
Key COSY (^1^H**―**^1^H) and HMBC (^1^H → ^13^C) correlations for compounds **1**–**3**.

### New Cladobotric Acid Analogues (**12**–**20**) Produced by Semisynthesis

**Scheme 1:**
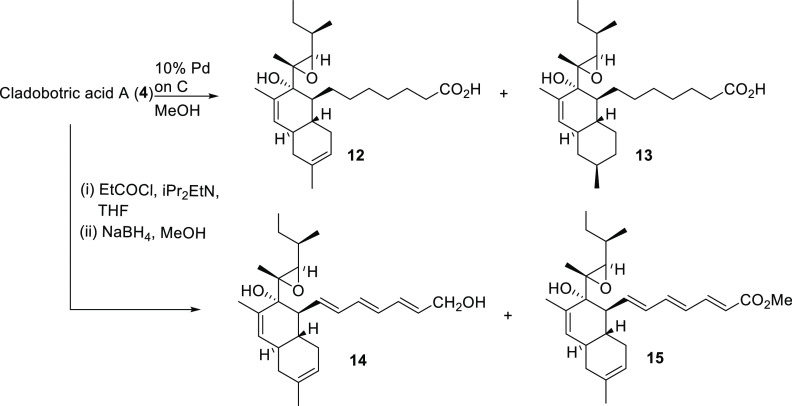
Reduction of Cladobotric Acid A (**4**)

The major metabolites isolated from extracts of *Cladobotryum* sp. grown on rice were cladobotric acid A (**4**) (600 mg) and pyrenulic acid A (**10**) (85 mg), providing sufficient material to use as starting materials for the semisynthesis of analogues for structure–activity studies on this family of polyketide-derived natural products. Reduction of **4** with H_2_ and 10% Pd on C gave a complex mixture of products, from which two pure compounds, **12** and **13** (4.5% and 13.5% yield, respectively), were isolated using reverse-phase HPLC (Scheme 1). In both cases the 8-trienoic acid side-chain of cladobotric acid A had been reduced, giving **12** as one of the products. In the second compound (**13**) it was evident that one of the trisubstituted alkenes had also been reduced, giving a single diastereomer. Extensive 2D NMR investigations revealed that the 11,12-alkene had been reduced, giving the equatorial methyl group at C-12, as determined from the coupling constants for 13-H_ax_ (app. q, *J* 12.5 Hz) that were in accord with a geminal and two axial–axial couplings and from NOE correlations between 8-H/14-H and 12-H/14-H.

Next, reduction of the carboxylic acid of **4** was investigated via generation of the mixed anhydride using propionyl chloride in the presence of ^i^Pr_2_EtN followed by treatment with NaBH_4_ in MeOH (Scheme 1). Two products were obtained, which were purified by HPLC to give primary alcohol **14** and methyl ester **15** (from reaction of the mixed anhydride with MeOH) in 11% and 48% yield, respectively.

**Scheme 2:**
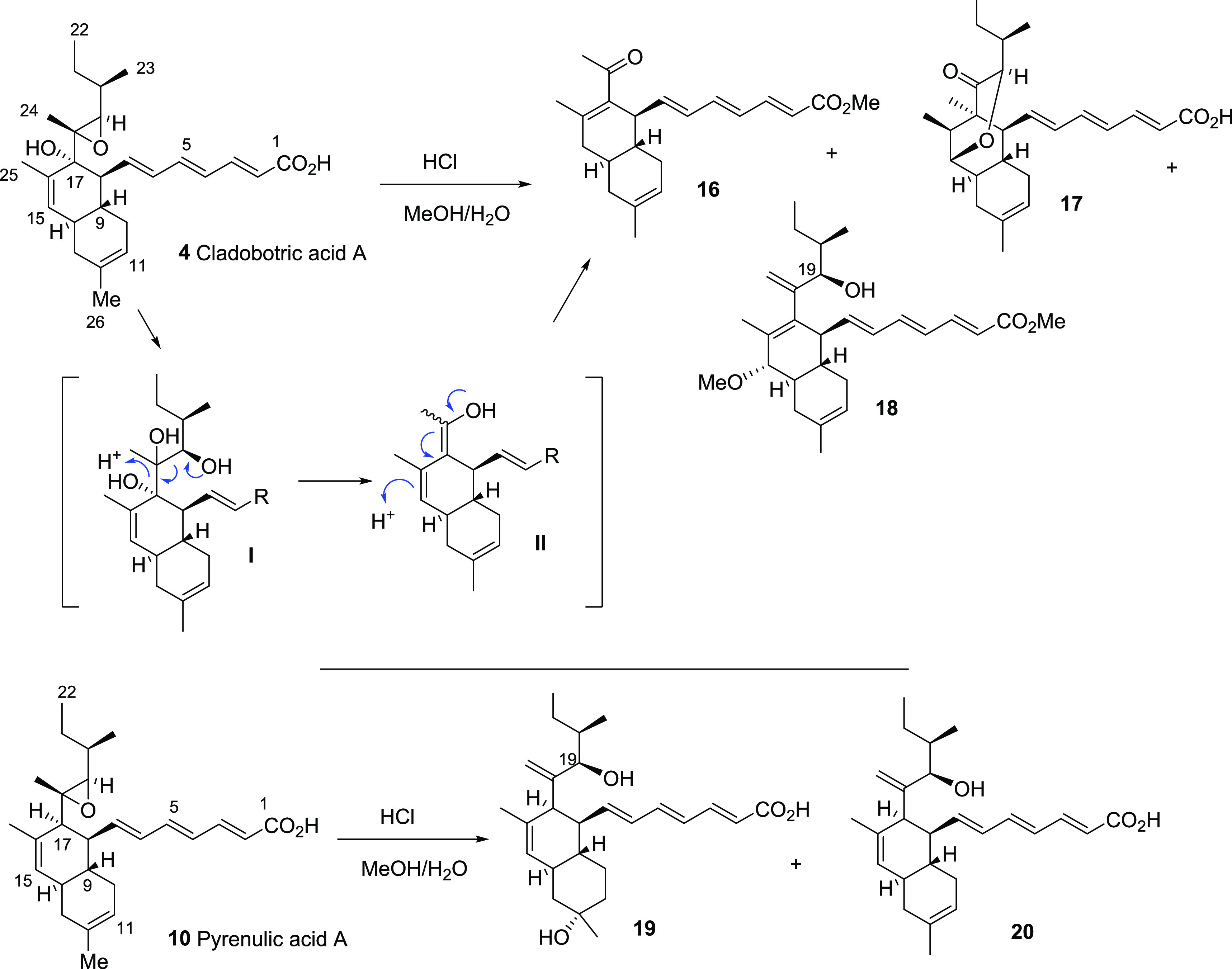
Acid-Mediated Reactions of Cladobotric Acid A (**4**) and Pyrenulic Acid A (**10**) and Proposed Mechanism for Fragmentation to Give **16**

Attention was then turned to the reaction of cladobotric acid A (**4**) with HCl in MeOH/H_2_O. Three products were isolated by reverse-phase HPLC, and their structures confirmed by extensive spectroscopic studies (Scheme 2). Compound **16** (33% yield) was a pale yellow solid with the molecular formula C_22_H_28_O_3_ (HRMS *m*/*z* 363.1940 [M + Na]^+^, calcd for C_22_H_28_O_3_Na, 363.1936), indicating that fragmentation had occurred as the product had four fewer carbon atoms than the starting material. The ^13^C NMR showed 22 signals including a carbonyl at δ 205.3, while the ^1^H NMR revealed a singlet at δ 1.89 (3H) in accord with loss of C-20–C-23 from the side-chain and formation of a methyl ketone. Furthermore, it was evident that 17-OH had been lost with generation of an α,β-unsaturated ketone **16**. It is proposed that **16** is formed via acid-mediated ring opening of the 18,19-epoxide to diol **I** followed by fragmentation to enol **II** and tautomerization to the enone (Scheme 2). The HRMS (*m*/*z* 411.2524 [M – H]^−^ calcd for C_26_H_35_O_4_, 411.2535) of the second compound, **17** (15% yield), showed it to have the same molecular formula as cladobotric acid A (**4**), but the NMR spectra lacked signals arising from the 18,19-epoxide, the 15,16-double bond, and 17-OH. New signals included a ketone carbonyl (δ_C_ 213.4) as well as two oxygenated methines, δ_C_ 82.1, δ_H_ 3.66 (1H, d, *J* 3.5 Hz) and δ_C_ 79.3, δ_H_ 3.88 (1H, s), and a new quaternary carbon δ_C_ 55.3 assigned to C-17. Hence, it was evident that a methyl shift had occurred from C-18 to C-17. NOE studies revealed correlations of 14-H/16-H and 19-H/24-H_3_ in accord with the structure **17**. Ketone **17** is also likely to be formed via diol **I**, but in this case, acid-mediated loss of 17-OH occurs with creation of the C-19–C-15 ether bridge and migration of 24-CH_3_ to C-17 generates the new carbon framework of **17**. The spectroscopic data of the final compound, **18** (30% yield), to be isolated from treatment of cladobotric acid A (**4**) with HCl in MeOH/H_2_O revealed that the carboxylic acid had been esterified to a methyl ester, δ_H_ 3.74 (3H, s) and δ_C_ 51.7, as well as the introduction of a methyl ether at C-15, δ_C_ 54.7, δ_H_ 3.27 (3H, s). NOE correlations were apparent between 9-H/15-H, confirming the stereochemistry of the 15-methoxy group. Furthermore, the epoxide had been transformed to an allylic alcohol with characteristic NMR signals for the *exo*-alkene at δ_H_ 5.33 and 4.89 (each 1H, each s) and δ_C_ 114.1 (C-24) and 150.9 (C-18) and an NOE 8-H/19-H in accord with the proposed structure **18**.

Pyrenulic acid A (**10**) was treated under the same acidic conditions as cladobotric acid A (**4**), and the products were isolated by HPLC (Scheme 2). In this case two products, **19** and **20** (27% and 15% yield, respectively), were fully characterized, with both possessing a C-17 side-chain incorporating an allylic alcohol formed by the acid-mediated rearrangement of the 18,19-epoxide, with NOE experiments confirming retention of stereochemistry at C-19 (as also found in ester **18**). For compound **19** hydration of the 11,12-alkene had also occurred, giving the tertiary alcohol at C-12.

### Bioactivity Screening

With a series of new and known cladobotric acids as well as semisynthetic derivatives available, their antibacterial activities were assessed against the Gram-negative bacterium *Escherichia coli* and three different strains of the Gram-positive pathogen *S. aureus*, comprising one methicillin-susceptible (MSSA), one methicillin-resistant and vancomycin-intermediate (MRSA/VISA), and one heterogeneous vancomycin-intermediate (hVISA) strain. The results of these investigations are shown in Table 2. None of the compounds tested revealed any activity against *E. coli* (data not shown). In contrast, with the exception of cladobotric acid F (**9**), all compounds tested showed detectable activity against all three *S. aureus* strains at concentrations ≤ 128 μg/mL. Overall, significant (i.e., minimum inhibitory concentrations (MIC) values more than one dilution apart) strain-dependent activity differences were apparent only for compounds **11** and **19**, where in both cases potency increased for the hVISA, compared to the MSSA strain. Pyrenulic acid B (**11**) showed significant antibacterial activity against both antibiotic-susceptible (MSSA) and -resistant (MRSA/VISA and hVISA) *S. aureus* strains with MIC values ranging from 4 to 16 μg/mL, while cladobotric acids A (**4**) and I (**3**) and pyrenulic acid A (**10**) exhibited moderate activities (MIC values from 16 to 64 μg/mL) (Table 2). Analysis of the results of assay data for these compounds suggested that compounds lacking an 18,19-epoxide showed enhanced antibacterial activity, for example, comparing **11**, with an 18,19-alkene (MIC values from 4 to 16 μg/mL), with the pyrenulic acid A (**10**) (MIC values 16 to 32 μg/mL). The presence of a 17-OH leads to a decrease in activity compared to the analogous natural products with a 17-H, as evidenced by the decrease in activity of **4**, **8**, and **3** (MIC range 16–128 μg/mL) in comparison with **10**, **2**, and **11**, (MIC range 4–64 μg/mL; Table 2). Compounds containing C-26 methyl esters (e.g., cladobotric acids E, F, and H, **8**, **9**, and **2**, respectively) tend to exhibit reduced activity (MICs ≥ 64 μg/mL). A similar pattern of antimicrobial activity was observed against *Bacillus subtilis* compared with *S. aureus* (Supporting Information, Table S1).

**Table 2:** Minimum Inhibitory Concentrations (MICs) of Tested Compounds against *Staphylococcus aureus* Strains

	bacterial strain (MIC, μg/mL)		
compound	MSSA	MRSA/VISA	hVISA
**1**	64	64	64
**2**	64	64	64
**3**	16	16	32
**4**	64	64	32
**5**	128	128	64
**8**	128	128	128
**9**	>256	>256	>256
**10**	32	16	16
**11**	16	8	4
**12**	32	32	32
**13**	16	32	16
**14**	64	64	64
**15**	128	128	128
**16**	256	128	256
**17**	64	128	64
**18**	128	64	64
**19**	64	32	16
20	4	4	4
pseudomonic acid A^a^	0.125	0.125	0.125
vancomycin^a^	2	4	4

a Positive control.

To further analyze the structure–activity relationship of this class of cladobotric acids, antibacterial activities of the semisynthetic derivatives **12**–**20** were examined (Table 2). The most active compound of all those tested was allylic alcohol **20** (MIC value 4 μg/mL), which lacks the 18,19-epoxide and has a 17-H rather than 17-OH, in accordance with the SAR results obtained from studies on the natural products. Reduction of the triene in the C-8 side-chain has little effect on bioactivity (comparing the activities of **4** and **12**), but the carboxylic acid at C-1 appears important, as methyl esters **9**, **15**, **16**, and **18** all exhibited reduced activity, e.g., comparing **8** and **9** (MICs of 128 and >256 μg/mL, respectively).

In conclusion, the three new cladobotric acids G–I (**1**–**3**) in addition to six known metabolites (**4**, **5**, **8**–**11**) have been isolated from *Cladobotryum* sp. CANU E1042, and their structures confirmed by spectroscopic methods. The major metabolites, cladobotric acid A (**4**) and pyrenulic acid A (**10**), were converted to a series of novel analogues by semisynthesis. The antibacterial activities against methicillin- and vancomycin-susceptible and resistant *S. aureus* bacteria (MSSA, MRSA/VISA, and hVISA) were tested, indicating that anti-Gram-positive activity was largely independent of methicillin and vancomycin susceptibility and revealing key structural features for biological activity. A carboxylic acid at C-1 was important (cf. a methyl ester or alcohol at C-1 significantly reduced activity), and in general compounds lacking a 17-OH tended to be more active. The 18,19-epoxide does not appear to be important for bioactivity. Indeed, compounds lacking this moiety (e.g., **11** and **20**) exhibited greater activity. As polyketides with an unusual carbon folding pattern,^10,17^ further studies on the biosynthetic gene cluster encoding cladobotric acid biosynthesis are ongoing in our laboratories, with the biosynthetic pathway likely to be similar to the those for related compounds such as fusarielin^18^ and burnettiene A.^19^

## Experimental Section

### General Experimental Procedures

Optical rotations were measured using a Bellingham and Stanley Ltd. ADP220 polarimeter. UV spectra were recorded in MeOH on a PerkinElmer Lambda 25 UV/vis spectrometer. IR spectra were obtained using a PerkinElmer Spectrum One FT-IR spectrometer as a film on KBr discs. NMR spectra were recorded on a Bruker Advance III HD Cryo 500 MHz spectrometer with TMS (tetramethylsilane) as the reference. Full assignment of NMR data was achieved using 2D experiments including COSY (^1^H–^1^H correlation spectroscopy), HSQC (heteronuclear single quantum coherence), HMBC (heteronuclear multiple-bond correlation), and NOESY (nuclear Overhauser effect spectroscopy). HRESIMS (high-resolution electrospray ionization mass spectrometry) data were recorded on a MicrO-TOF II (Bruker, Daltonics) mass spectrometer. Silica gel (Merck, 63–200 μm particle size), RP-18 (Merck, 40–63 μm particle size), and Sephadex LH-20 were used for column chromatography. TLC (thin layer chromatography) was carried out with silica gel 60 F_254_ and RP-18 F_254_ plates. HPLC (high-performance liquid chromatography) was carried out using a Waters system using a Phenomenex Kinetex C_18_ column (10 × 250 mm, 5 μm particle size). Detection was achieved by a Waters 2998 diode array, a Waters Quattro Micro ESI mass spectrometer, and a Waters 2424 evaporative light scattering detector. All solvents used for extraction and isolation were of analytical grade.

### Fungal Material

The fungal strain *Cladobotryum* sp. CANU E1042 was isolated from a podocarp forest near Hokotika, New Zealand, and provided to us by Munro and co-workers.^10^

### Fermentation, Extraction, and Purification

The fungus *Cladobotryum* sp. CANU E1042 was inoculated in a 500 mL Erlenmeyer flask containing 100 mL of a PDB medium (2.4% potato dextrose broth). The flask was incubated statically at 18 °C for 3 days. Aliquots of 20 mL of this seed culture were transferred to five Erlenmeyer flasks, each containing white rice (100 g), soaked in H_2_O (100 mL) and autoclaved as a solid production medium. The flasks were incubated statically at 18 °C for 14 days. The solid fermentation was then extracted with EtOAc (3 L) by blending and sonicating for 30 min at room temperature. The extract was filtered and concentrated under vacuum to obtain 8.0 g of crude extract. This crude extract was then chromatographed using a silica gel column (4 × 30 cm; 63–200 μm particle size) and eluted with an *n*-hexane/acetone series (9:1, 8:2, ..., 1:9, each 0.5 L) to yield seven fractions (F1: 1.5 g; F2: 1.2 g; F3: 0.8 g; F4: 0.6 g; F5: 1.6 g; F6: 0.4 g; F7: 0.5 g). Fractions F4–F6 showed inhibitory effects on a diffusion paper disc assay against *S. aureus* (Mu50). These fractions were analyzed to characterize which compounds were responsible for this antibacterial activity. Fraction F4 was applied to an RP-18 column (3 × 20 cm; 40 μm particle size) and eluted with a stepwise gradient of MeOH/H_2_O (1:1 to 4:1) to afford four subfractions (F4.1–F4.4). F4.3 (230 mg) was further purified by HPLC [Phenomenex Kinetex C_18_ column (10 × 250 mm, 5 μm particle size); mobile phase MeCN in H_2_O containing 0.1% HCO_2_H (0–15 min: 87% MeCN, 15–20 min: 87–100% MeCN); flow rate 16 mL/min] to yield compounds **10** (*t*_R_ = 12.5 min, 85.0 mg, >98% purity) and **11** (*t*_R_ = 16.0 min, 6.0 mg, >98% purity). Fraction F5 was chromatographed over a Sephadex LH-20 column (3 × 30 cm) using MeOH as the eluting solvent to give three subfractions (F5.1–F5.3). From fraction F5.2 (1.1 g) the major compound **4** (600 mg, >97% purity) was crystallized from MeOH. The mother liquor was purified by HPLC (0–15 min: 73% MeCN, 15–20 min: 73–100% MeCN) to yield compounds **1** (*t*_R_ = 10.0 min, 4.5 mg, >98% purity), **2** (*t*_R_ = 10.8 min, 6.5 mg, >98% purity), and **9** (*t*_R_ = 12.5 min, 9.0 mg, >98% purity). Finally, compounds **5** (*t*_R_ = 7.8 min, 7.0 mg, >98% purity), **8** (*t*_R_ = 9.2 min, 12.0 mg, >98% purity), and **3** (*t*_R_ = 14.5 min, 4.5 mg, >98% purity) were purified by HPLC (0–10 min: 68% MeCN, 10–20 min: 68–100% MeCN) from fraction F6.

#### Cladobotric acid G (**1**):

pale yellow solid; [α]_D_ −70.2 (*c* 0.1, CHCl_3_); UV (MeOH) λ_max_ (log ε) 302 (4.21) nm; IR (KBr) ν_max_ 3407, 2962, 1694, 1616, 1381, 1003 cm^–1^; ^1^H (500 MHz) and ^13^C (125 MHz) NMR data, see Table 1; HRESIMS *m*/*z* 451.2470 [M + Na]^+^ (calcd for C_26_H_36_O_5_Na, 451.2460).

#### Cladobotric Acid H (**2**):

pale yellow solid; [α]_D_ −26.7 (*c* 0.15, CHCl_3_); UV (MeOH) λ_max_ (log ε) 305 (4.08) nm; IR (KBr) ν_max_ 3431, 2962, 1712, 1647, 1437, 1257, 1049 cm^–1^; ^1^H (500 MHz) and ^13^C (125 MHz) NMR data, see Table 1; HRESIMS *m*/*z* 463.2468 [M + Na]^+^ (calcd for C_27_H_36_O_5_Na, 463.2460).

#### Cladobotric acid I (**3**):

pale yellow solid; [α]_D_ −60.4 (*c* 0.15, CHCl_3_); UV (MeOH) λ_max_ (log ε) 306 (3.85) nm; IR (KBr) ν_max_ 3422, 2959, 1704, 1615, 1377, 1251, 1048 cm^–1^; ^1^H (500 MHz) and ^13^C (125 MHz) NMR data, see Table 1; HRESIMS *m*/*z* 419.2544 [M + Na]^+^ (calcd for C_26_H_36_O_3_Na, 419.2557).

### Reduction of Cladobotric Acid A (**4**) to Compounds **12** and **13**

A solution of **4** (100 mg, 0.25 mmol) in MeOH (3 mL) was treated with 10% Pd/C (5 mg, 0.005 mmol) and stirred under an atmosphere of H_2_ for 2 h. The reaction mixture was filtered through Celite and purified by HPLC (0–15 min: 80% MeCN, 15–20 min: 80–100% MeCN) to yield compounds **12** (*t*_R_ = 11.0 min, 4.5 mg, >98% purity) and **13** (*t*_R_ = 13.8 min, 13.5 mg, >98% purity).

#### **12**:

pale yellow solid; [α]_D_ −33.2 (*c* 0.1, CHCl_3_); UV (MeOH) λ_max_ (log ε) 228 (3.20) nm; IR (KBr) ν_max_ 3530, 2924, 1708, 1459, 1378, 1297, 1023 cm^–1^; ^1^H NMR (500 MHz, CDCl_3_) δ 5.43 (1H, br s, H-11), 5.41 (1H, br s, H-15), 2.97 (1H, d, *J* = 8.0 Hz, H-19), 2.36 (2H, m, H-2), 2.21 (1H, m, H-8), 2.12 (3H, m, H-6 and H-10a), 1.97 (1H, m, H-14), 1.68 (3H, s, H-25), 1.67–1.58 (3H, m, H-3, H-21a), 1.54–1.50 (3H, m, H-9, H-10b and H-13a), 1.47–1.44 (3H, m, H-4 and H-7a), 1.35 (3H, s, H-24), 1.33–1.25 (5H, m, H-5, H-13b, H-20 and H-21b), 0.98–0.94 (7H, m, H-7b, H-22, and H-26), 0.93 (3H, m, H-23); ^13^C NMR (125 MHz, CDCl_3_) δ 177.9 (C-1), 134.5 (C-15), 133.7 (C-12), 133.6 (C-16), 128.5 (C-11), 75.1 (C-17), 62.7 (C-18), 62.5 (C-19), 58.9 (C-8), 42.9 (C-9), 38.6 (C-13), 36.6 (C-14), 34.5 (C-20), 33.6 (C-2), 32.0 (C-6), 31.9 (C-10), 28.8 (C-4), 27.7 (C-5), 27.9 (C-21), 25.6 (C-7), 24.1 (C-3), 18.3 (C-25 and C-26), 15.6 (C-24), 15.5 (C-23), 11.3 (C-22); HRESIMS *m*/*z* 441.2972 [M + Na]^+^ (calcd for C_26_H_42_O_4_Na, 441.2975).

#### **13**:

pale solid; [α]_D_ −29.2 (*c* 0.15, CHCl_3_); UV (MeOH) λ_max_ (log ε) 227 (3.95) nm; IR (KBr) ν_max_ 2924, 1708, 1456, 1379, 1285, 1048 cm^–1^; ^1^H NMR(500 MHz, CDCl_3_) δ 5.44 (1H, br s, H-15), 2.97 (1H, d, *J* = 8.0 Hz, H-19), 2.33 (2H, t, *J* = 7.0 Hz, H-2), 1.89–1.78 (3H, m, H-7a, H-10a and H-11a), 1.74 (1H, m, H-14), 1.70 (1H, m, H-13a), 1.68 (3H, s, H-25), 1.65–1.60 (3H, m, H-3 and H-21a), 1.55 (1H, m, H-8), 1.45 (1H, m, H-12), 1.40 (1H, m, H-9), 1.39–1.32 (7H, m, H-4, H-5a, H-6a, H-7b, H-20 and H-21b), 1.29 (3H, s, H-24), 1.28–1.20 (2H, m, H-5b and H-6b), 1.03–0.88 (11H, m, H-10b, H-11b, H-22, H-23, and H-26), 0.71 (1H, q, *J* = 12.5 Hz, H-13b); ^13^C NMR (125 MHz, CDCl_3_) δ 179.6 (C-1), 135.0 (C-15), 133.8 (C-16), 76.5 (C-17), 63.1 (C-18), 62.9 (C-19), 53.5 (C-8), 44.6 (C-9), 43.3 (C-14), 41.9 (C-13), 35.9 (C-11), 34.6 (C-20), 34.1 (C-2), 32.9 (C-12), 32.0 (C-6), 30.5 (C-10), 29.9 (C-5), 29.2 (C-4), 28.2 (C-21), 26.3 (C-7), 24.9 (C-3), 22.7 (C-26), 18.7 (C-25), 15.8 (C-23), 15.7 (C-24), 11.5 (C-22); HRESIMS *m*/*z* 443.3133 [M + Na]^+^ (calcd for C_26_H_44_O_4_Na, 443.3132).

### Conversion of Cladobotric Acid A (**4**) to Compounds **14** and **15**

A solution of **4** (50 mg, 0.125 mmol) in THF (2 mL) was added with EtCOCl (15 mg, 0.162 mmol) and DIPEA (32 mg, 0.25 mmol) and stirred under a N_2_ atmosphere at 0 °C for 30 min. The reaction mixture was then filtered and concentrated under reduced pressure. The crude reaction mixture was dissolved in MeOH (2 mL), treated with NaBH_4_ (24 mg, 0.625 mmol), and stirred at −78 °C for 3 h. On completion of the reaction, it was quenched by the addition of a saturated NH_4_Cl solution (2 mL), diluted with H_2_O (8 mL), and extracted with EtOAc (3 × 10 mL). The combined organic extracts were washed with brine (20 mL), dried over anhydrous MgSO_4_, filtered, and concentrated under reduced pressure. The residue was purified by HPLC (0–15 min: 82% MeCN, 15–20 min: 82–100% MeCN) to yield compounds **14** (*t*_R_ = 12.5 min, 5.5 mg, >98% purity) and **15** (*t*_R_ = 15.8 min, 23.5 mg, >98% purity).

#### **14**:

pale yellow solid; [α]_D_ −87.5 (*c* 0.1, CHCl_3_); UV (MeOH) λ_max_ (log ε) 271 (3.38) nm; IR (KBr) ν_max_ 3353, 2973, 1381, 1086, 1045 cm^–1^; ^1^H NMR (500 MHz, CDCl_3_) δ 6.33 (1H, dd, *J* = 15.0, 11.0 Hz, H-5), 6.26 (1H, dd, *J* = 15.0, 11.0 Hz, H-3), 6.16 (1H, dd, *J* = 15.0, 11.0 Hz, H-4), 6.10 (1H, dd, *J* = 15.0, 10.0 Hz, H-7), 5.83 (1H, dt, *J* = 15.0, 10.0 Hz, H-2), 5.77 (1H, dd, *J* = 15.0, 11.0 Hz, H-6), 5.57 (1H, br s, H-15), 5.36 (1H, br s, H-11), 4.19 (2H, d, *J* = 6.0 Hz, H-1), 2.97 (1H, d, *J* = 8.0 Hz, H-19), 2.30 (1H, t, *J* = 11.0 Hz, H-8), 2.04 (2H, m, H-13a, H-14), 1.96–1.85 (2H, m, H-9 and H-10a), 1.78 (1H, m, H-13b), 1.72 (3H, s, H-25), 1.66 (3H, s, H-26), 1.63 (1H, m, H-21a), 1.49 (1H, m, H-10b), 1.37 (3H, s, H-24), 1.34–1.25 (2H, m, H-20 and H-21b), 0.95–0.92 (6H, m, H-22 and H-23); ^13^C NMR (125 MHz, CDCl_3_) δ 134.1 (C-12), 133.8 (C-16), 133.6 (C-7), 133.2 (C-5), 132.9 (C-6), 132.8 (C-15), 131.9 (C-2), 131.8 (C-3), 130.8 (C-4), 121.5 (C-11), 75.4 (C-17), 63.7 (C-1), 63.1 (C-19), 62.5 (C-18), 59.0 (C-8), 38.7 (C-14), 38.2 (C-9), 37.3 (C-13), 34.6 (C-20), 31.9 (C-10), 27.9 (C-21), 23.6 (C-26), 18.4 (C-25), 15.7 (C-24), 15.5 (C-23), 11.4 (C-22); HRESIMS *m*/*z* 397.2742 [M – H]^−^ (calcd for C_26_H_37_O_3_, 397.2743).

#### **15**:

pale yellow solid; [α]_D_ −50.2 (*c* 0.15, CHCl_3_); UV (MeOH) λ_max_ (log ε) 305 (4.40) nm; IR (KBr) ν_max_ 3450, 2961, 1716, 1616, 1435, 1244, 1006 cm^–1^; ^1^H NMR (500 MHz, CDCl_3_) δ 7.29 (1H, dd, *J* = 15.5, 11.5 Hz, H-3), 6.63 (1H, dd, *J* = 15.0, 11.0 Hz, H-5), 6.24 (1H, dd, *J* = 15.0, 11.0 Hz, H-4), 6.16 (1H, dd, *J* = 15.0, 10.0 Hz, H-6), 5.98 (1H, dd, *J* = 15.0, 11.0 Hz, H-7), 5.86 (1H, d, *J* = 15.0 Hz, H-2), 5.57 (1H, br s, H-15), 5.36 (1H, br s, H-11), 3.73 (3H, s, 1-OC*H*_*3*_), 2.96 (1H, d, *J* = 8.5 Hz, H-19), 2.34 (1H, t, *J* = 11.0 Hz, H-8), 2.04 (2H, m, H-13a, H-14), 1.95–1.85 (2H, m, H-9 and H-10a), 1.78 (1H, m, H-13b), 1.72 (3H, s, H-25), 1.66 (3H, s, H-26), 1.63 (1H, m, H-21a), 1.49 (1H, m, H-10b), 1.37 (3H, s, H-24), 1.34–1.24 (2H, m, H-20 and H-21b), 0.95–0.92 (6H, m, H-22 and H-23); ^13^C NMR (125 MHz, CDCl_3_) δ 167.7 (C-1), 144.9 (C-3), 140.6 (C-5), 137.6 (C-7), 134.2 (C-12), 133.7 (C-16), 133.1 (C-6), 132.8 (C-15), 128.9 (C-4), 121.3 (C-11), 120.3 (C-2), 75.5 (C-17), 63.2 (C-19), 62.5 (C-18), 59.2 (C-8), 51.6 (1-O*C*H_3_), 38.6 (C-14), 38.1 (C-9), 37.3 (C-13), 34.6 (C-20), 31.9 (C-10), 27.9 (C-21), 23.5 (C-26), 18.3 (C-25), 15.6 (C-24), 15.5 (C-23), 11.4 (C-22); HRESIMS *m*/*z* 449.2666 [M + Na]^+^ (calcd for C_27_H_38_O_4_Na, 449.2668).

### Treatment of Either Cladobotric Acid A (**4**) or Pyrenulic Acid A (**10**) with Acid

Concentrated HCl (2 drops) was added to a solution of **4** (50 mg) in MeOH (2.5 mL) and H_2_O (0.5 mL). The resulting solution was stirred at reflux for 48 h. The reaction mixture was poured into cold H_2_O (5 mL) and extracted with CH_2_Cl_2_ (3 × 8 mL). The combined organic extracts were washed with brine (20 mL), dried over anhydrous MgSO_4_, filtered, and concentrated under reduced pressure. The residue was purified by HPLC (0–15 min: 80% MeCN, 15–20 min: 80–100% MeCN) to yield compounds **16** (*t*_R_ = 11.0 min, 16.5 mg, >98% purity), **17** (*t*_R_ = 12.0 min, 7.5 mg, >97% purity), and **18** (*t*_R_ = 16.0 min, 15.0 mg, >98% purity).

The above procedure was repeated starting with pyrenulic acid A (**10**), giving **19** (*t*_R_ = 6.5 min, 13.5 mg, >98% purity) and **20** (*t*_R_ = 17.5 min, 7.5 mg, >98% purity).

#### **16**:

pale yellow solid; [α]_D_ −7.4 (*c* 0.1, CHCl_3_); UV (MeOH) λ_max_ (log ε) 302 (4.36) nm; IR (KBr) ν_max_ 2891, 1714, 1614, 1433, 1245, 1004 cm^–1^; ^1^H NMR (500 MHz, CDCl_3_) δ 7.28 (1H, dd, *J* = 15.5, 11.5 Hz, H-3), 6.50 (1H, dd, *J* = 15.0, 11.0 Hz, H-5), 6.24 (1H, dd, *J* = 15.0, 11.0 Hz, H-4), 6.09 (1H, dd, *J* = 15.0, 10.0 Hz, H-6), 5.89 (1H, dd, *J* = 15.0, 11.0 Hz, H-7), 5.86 (1H, d, *J* = 15.0 Hz, H-2), 5.33 (1H, br s, H-11), 3.74 (3H, s, 1-OC*H*_*3*_), 3.11 (1H, d, *J* = 11.0 Hz, H-8), 2.33 (2H, m, H-15), 2.04 (2H, m, H-10), 1.89 (3H, s, H-20), 1.84 (2H, m, H-13), 1.79 (3H, s, H-19), 1.65 (2H, m, H-9 and H-14), 1.62 (3H, s, H-21); ^13^C NMR (125 MHz, CDCl_3_) δ 205.3 (C-18), 167.7 (C-1), 145.9 (C-16), 144.7 (C-3), 140.4 (C-5), 138.2 (C-7), 133.7 (C-12), 132.5 (C-17), 131.5 (C-6), 129.4 (C-4), 121.0 (C-11), 120.5 (C-2), 63.9 (C-8), 51.7 (1-O*C*H_3_), 43.5 (C-14), 42.7 (C-15), 39.8 (C-9), 39.3 (C-13), 33.3 (C-10), 23.3 (C-20), 23.2 (C-21), 16.0 (C-19); HRESIMS *m*/*z* 363.1940 [M + Na]^+^ (calcd for C_22_H_28_O_3_Na, 363.1936).

#### **17**:

pale yellow solid; [α]_D_ −13.0 (*c* 0.1, CHCl_3_); UV (MeOH) λ_max_ (log ε) 300 (4.05) nm; IR (KBr) ν_max_ 3440, 2961, 1691, 1614, 1382, 1243, 1005 cm^–1^; ^1^H NMR (500 MHz, CDCl_3_) δ 7.37 (1H, dd, *J* = 15.5, 11.5 Hz, H-3), 6.55 (1H, dd, *J* = 15.0, 11.0 Hz, H-5), 6.28 (1H, dd, *J* = 15.0, 11.0 Hz, H-4), 6.02 (1H, dd, *J* = 15.0, 10.0 Hz, H-6), 5.87 (1H, d, *J* = 15.0 Hz, H-2), 5.51 (1H, dd, *J* = 15.0, 11.0 Hz, H-7), 5.28 (1H, br s, H-11), 3.78 (1H, s, H-15), 3.66 (1H, d, *J* = 3.5 Hz, H-19), 2.51 (1H, m, H-8), 2.39 (1H, m, H-16), 2.07 (1H, m, H-14), 2.02 (1H, m, H-10a), 1.93 (1H, m, H-13a), 1.75 (1H, m, H-21a), 1.71 (1H, m, H-20), 1.68 (1H, m, H-13b), 1.62 (3H, s, H-26), 1.54 (1H, m, H-10b), 1.41 (1H, m, H-21b), 1.36 (3H, d, *J* = 7.5 Hz, H-25), 1.05 (1H, m, H-9), 1.01 (3H, d, *J* = 7.0 Hz, H-23), 0.97 (3H, t, *J* = 7.0 Hz, H-22), 0.93 (3H, s, H-24); ^13^C NMR (125 MHz, CDCl_3_) δ 213.4 (C-18), 170.9 (C-1), 146.9 (C-3), 141.3 (C-5), 140.4 (C-7), 133.1 (C-12), 131.2 (C-6), 128.8 (C-4), 120.8 (C-11), 119.5 (C-2), 82.1 (C-19), 79.3 (C-15), 55.3 (C-17), 50.3 (C-8), 46.6 (C-14), 42.9 (C-16), 37.7 (C-9), 36.7 (C-20), 35.9 (C-13), 33.8 (C-10), 25.6 (C-21), 23.1 (C-26), 19.9 (C-23), 18.8 (C-24), 18.4 (C-25), 12.4 (C-22); HRESIMS *m*/*z* 411.2524 [M – H]^−^ (calcd for C_26_H_35_O_4_, 411.2535).

#### **18**:

yellowish solid; [α]_D_ −17.5 (*c* 0.1, CHCl_3_); UV (MeOH) λ_max_ (log ε) 305 (4.32) nm; IR (KBr) ν_max_ 3486, 2959, 1715, 1616, 1434, 1241, 1005 cm^–1^; ^1^H NMR (500 MHz, CDCl_3_) δ 7.28 (1H, dd, *J* = 15.5, 11.5 Hz, H-3), 6.47 (1H, dd, *J* = 15.0, 11.0 Hz, H-5), 6.24 (1H, dd, *J* = 15.0, 11.0 Hz, H-4), 6.04 (1H, dd, *J* = 15.0, 10.0 Hz, H-6), 5.84 (1H, d, *J* = 15.0 Hz, H-2), 5.48 (1H, dd, *J* = 15.0, 11.0 Hz, H-7), 5.34 (1H, br s, H-11), 5.33 (1H, s, H-24a), 4.89 (1H, s, H-24b), 3.91 (1H, s, H-19), 3.74 (3H, s, 1-OC*H*_*3*_), 3.70 (1H, m, H-15), 3.27 (3H, s, 15-OC*H*_*3*_), 2.57 (1H, m, H-8), 2.38 (1H, m, H-13a), 2.16 (1H, m, H-10a), 1.84 (1H, m, H-14), 1.81 (1H, m, H-13b), 1.76 (3H, s, H-25), 1.66 (3H, s, H-26), 1.60 (1H, m, H-10b), 1.51 (1H, m, H-21a), 1.43 (1H, m, H-20), 1.38 (1H, m, H-9), 1.06 (1H, m, H-21b), 0.98 (3H, d, *J* = 7.0 Hz, H-23), 0.85 (3H, t, *J* = 7.5 Hz, H-22); ^13^C NMR (125 MHz, CDCl_3_) δ 167.6 (C-1), 150.9 (C-18), 144.8 (C-3), 142.1 (C-7), 140.3 (C-5), 136.8 (C-17), 132.9 (C-12), 132.1 (C-16), 130.6 (C-6), 128.9 (C-4), 120.3 (C-2), 119.7 (C-11), 114.1 (C-24), 85.1 (C-15), 76.4 (C-19), 54.7 (15-O*C*H_3_), 51.7 (1-O*C*H_3_), 50.1 (C-8), 38.2 (C-20), 37.0 (C-14), 36.8 (C-9), 35.5 (C-13), 31.5 (C-10), 23.5 (C-26), 22.6 (C-21), 16.9 (C-23), 15.5 (C-25), 12.2 (C-22); HRESIMS *m*/*z* 463.2825 [M + Na]^+^ (calcd for C_28_H_40_O_4_Na, 463.2824).

#### **19**:

pale yellow solid; [α]_D_ −6.5 (*c* 0.1, CHCl_3_); UV (MeOH) λ_max_ (log ε) 306 (4.16) nm; IR (KBr) ν_max_ 3365, 2962, 1687, 1614, 1377, 1249, 1009 cm^–1^; ^1^H NMR (500 MHz, CDCl_3_) δ 7.37 (1H, dd, *J* = 15.5, 11.5 Hz, H-3), 6.60 (1H, dd, *J* = 15.0, 11.0 Hz, H-5), 6.26 (1H, dd, *J* = 15.0, 11.0 Hz, H-4), 6.18 (1H, dd, *J* = 15.0, 10.0 Hz, H-6), 5.85 (1H, d, *J* = 15.0 Hz, H-2), 5.76 (1H, dd, *J* = 15.0, 11.0 Hz, H-7), 5.36 (1H, s, H-24a), 5.30 (1H, br s, H-15), 5.04 (1H, s, H-24b), 3.89 (1H, s, H-19), 2.58 (1H, d, *J* = 6.5 Hz, H-17), 2.36 (1H, m, H-8), 2.11 (1H, m, H-14), 1.70 (1H, m, H-13a), 1.65 (1H, m, H-11a), 1.53 (3H, s, H-25), 1.50 (1H, m, H-21a), 1.48 (1H, m, H-20), 1.39 (1H, m, H-11b), 1.36 (1H, m, H-10a), 1.28 (1H, m, H-9), 1.24 (3H, s, H-26), 1.19 (1H, m, H-13b), 1.16 (1H, m, H-10b), 1.02 (1H, m, H-21b), 1.01 (3H, d, *J* = 7.0 Hz, H-23), 0.85 (3H, t, *J* = 7.5 Hz, H-22); ^13^C NMR (125 MHz, CDCl_3_) δ 170.6 (C-1), 150.7 (C-18), 147.0 (C-3), 142.9 (C-7), 141.7 (C-5), 133.9 (C-16), 130.9 (C-6), 128.8 (C-15), 128.3 (C-4), 119.3 (C-2), 113.6 (C-24), 80.1 (C-19), 70.2 (C-12), 49.3 (C-17), 49.0 (C-8), 45.8 (C-13), 39.4 (C-11), 37.7 (C-20), 37.6 (C-14), 36.7 (C-9), 31.7 (C-26), 26.2 (C-10), 22.2 (C-25), 21.2 (C-21), 12.1 (C-22); HRESIMS *m*/*z* 413.2685 [M – H]^−^ (calcd for C_26_H_37_O_4_, 413.2692).

#### **20**:

pale yellow solid; [α]_D_ −24.8 (*c* 0.1, CHCl_3_); UV (MeOH) λ_max_ (log ε) 304 (4.05) nm; IR (KBr) ν_max_ 3359, 2963, 1687, 1613, 1377, 1245, 1008 cm^–1^; ^1^H NMR (500 MHz, CDCl_3_) δ 7.37 (1H, dd, *J* = 15.5, 11.5 Hz, H-3), 6.59 (1H, dd, *J* = 15.0, 11.0 Hz, H-5), 6.26 (1H, dd, *J* = 15.0, 11.0 Hz, H-4), 6.19 (1H, dd, *J* = 15.0, 10.0 Hz, H-6), 5.85 (1H, d, *J* = 15.0 Hz, H-2), 5.77 (1H, dd, *J* = 15.0, 11.0 Hz, H-7), 5.43 (1H, br s, H-15), 5.34 (2H, br s, H-11 and H-24a), 5.05 (1H, s, H-24b), 3.88 (1H, s, H-19), 2.56 (1H, d, *J* = 6.5 Hz, H-17), 2.31 (1H, m, H-8), 2.03 (1H, m, H-13a), 1.98 (1H, m, H-14), 1.84 (1H, m, H-10a), 1.78 (1H, m, H-13b), 1.66 (3H, s, H-26), 1.59 (1H, m, H-9), 1.56 (3H, s, H-25), 1.53–1.47 (3H, m, H-10b, H-20 and H-21a), 1.04 (1H, m, H-21b), 1.01 (3H, d, *J* = 7.0 Hz, H-23), 0.85 (3H, t, *J* = 7.5 Hz, H-22); ^13^C NMR (125 MHz, CDCl_3_) δ 170.2 (C-1), 150.6 (C-18), 147.1 (C-3), 142.7 (C-7), 141.7 (C-5), 134.2 (C-12), 133.8 (C-16), 130.7 (C-6), 128.3 (C-4), 128.1 (C-15), 121.4 (C-11), 119.1 (C-2), 113.5 (C-24), 80.0 (C-19), 49.2 (C-17), 49.1 (C-8), 38.6 (C-14), 38.0 (C-13), 37.7 (C-20), 32.9 (C-9), 31.2 (C-10), 23.6 (C-26), 22.3 (C-25), 21.2 (C-21), 17.2 (C-23), 12.1 (C-22); HRESIMS *m*/*z* 395.2580 [M – H]^−^ (calcd for C_26_H_35_O_3_, 395.2586).

### Determination of the Minimum Inhibitory Concentration against Gram-Positive Bacterial Pathogens

Three *Staphylococcus aureus* strains, ATCC 29213 (MSSA),^20^*S. aureus* Mu50 (MRSA/VISA),^21^ and *S. aureus* 21773 (hVISA), provided by Alastair P. MacGowan, Bristol, UK, were used for the antibacterial assays. The MIC values were determined using the broth dilution method as proposed by the European Committee on Antimicrobial Susceptibility Testing (EUCAST),^24^ with pseudomonic acid A^22^ and vancomycin^23^ used as positive controls. Briefly, tested compounds were serially diluted in cation-adjusted Mueller-Hinton (M-H) broth. A 100 μL amount of the 2× stock containing the relevant compound was added to each well of a 96-well microtiter plate and subsequently diluted with 80 μL of M-H broth and 20 μL of bacterial suspension to give a final concentration of approximately 5 × 10^5^ cfu/mL. The microtiter plates were incubated at 37 °C for 18–24 h, and the OD_600_ was measured using a microplate reader (Polarstar Omega). The MIC values were taken as the lowest compound concentration resulting in the complete inhibition of bacterial growth. All assays were performed in three independent experiments, with triplicates per experiment.

## Supporting Information Available

Spectra of compounds **1**–**3** and **12**–**20** (PDF)

## Dedication

Dedicated to Dr. William H. Gerwick, University of California at San Diego, for his pioneering work on bioactive natural products.
