# Supplementary material for: Cladobotric Acids: Metabolites from Cultures of Cladobotryum sp., Semisynthetic Analogues and Antibacterial Activity
Source: J Nat Prod. 2022 Feb 16;85(3):572–80. doi: 10.1021/acs.jnatprod.1c01063 (PMC9097583; doi:10.1021/acs.jnatprod.1c01063)
Supplement: Supplementary file 1 — np1c01063_si_001.pdf [file np1c01063_si_001.pdf]

SUPPORTING INFORMATION

Cladobotric acids: Metabolites from cultures of *Cladobotryum* sp., semi-synthetic analogues and antibacterial activity

Trong-Tuan Dao,[†] Katherine Williams,[‡] Kate M. J. de Mattos-Shipley,[‡] Zhongshu Song,[†] Yuiko Takebayashi,[§] Thomas J. Simpson,[†] James Spencer,[§] Andrew M. Bailey,[‡] and Christine L. Willis*[†]*

[†]School of Chemistry, University of Bristol, Cantock's Close, Bristol, BS8 1TS, UK

[‡]School of Biological Sciences, Life Sciences Building, University of Bristol, 24 Tyndall Ave, Bristol, BS8 1TQ, UK

[§]School of Cellular and Molecular Medicine, University of Bristol, University Walk, Bristol, BS8 1TD, UK

[*] To whom correspondence should be addressed. E-mail: chris.willis@bristol.ac.uk

Table of Contents

Figure S1: HPLC profiles (ELSD) for cladobotric acids **1-5**, **8-11**, which were isolated from a crude extract of *Cladobotryum* sp. (bottom trace). 6

Figure S2: (+)ESI-MS spectrum of **1**. 7

Figure S3: HRESIMS spectrum of **1**. 7

Figure S4: IR spectrum of **1** (film, KBr disc) 8

Figure S5: UV/vis (DAD) spectrum of **1** (in MeOH) 8

Figure S6: ^{1}H NMR spectrum (500 MHz, $CDCl_3$) of **1**. 9

Figure S7: ^{13}C NMR spectrum (125 MHz, $CDCl_3$) of **1**. 9

Figure S8: COSY spectrum of **1**. 10

Figure S9: HSQC spectrum of **1**. 10

Figure S10: HMBC spectrum of **1**. 11

Figure S11: NOESY spectrum **1**. 11

Figure S12: (+)ESI-MS spectrum of **2**. 12

Figure S13: HRESIMS spectrum of **2**. 12

Figure S14: IR spectrum of **2** (film, KBr disc) 13

Figure S15: UV/vis (DAD) spectrum of **2** (in MeOH) 13

Figure S16: ^{1}H NMR spectrum (500 MHz, $CDCl_3$) of **2**. 14

Figure S17: ^{13}C NMR spectrum (125 MHz, $CDCl_3$) of **2**. 14

Figure S18: COSY spectrum of **2**. 15

Figure S19: HSQC spectrum of **2**. 15

Figure S20: HMBC spectrum of **2**. 16

Figure S21: NOESY spectrum **2**. 16

Figure S22: (+)ESI-MS spectrum of **3**. 17

Figure S23: HRESIMS spectrum of **3**. 17

Figure S24: IR spectrum of **3** (film, KBr disc) 18

Figure S25: UV/vis (DAD) spectrum of **3**. (MeOH) 18

Figure S26: ^{1}H NMR spectrum (500 MHz, $CDCl_3$) of **3**. 19

Figure S27: ^{13}C NMR spectrum (125 MHz, $CDCl_3$) of **3**. 19

Figure S28: COSY spectrum of **3**. 20

Figure S29: HSQC spectrum of **3**. 20

Figure S30: HMBC spectrum of **3**. 21

Figure S31: NOESY spectrum **3**. 21

Figure S32: Key NOESY correlations (indicated by blue arrows) for compounds **1–3** 22
Figure S33: (+)ESI-MS spectrum of **12**. 23
Figure S34: HRESIMS spectrum of **12**. 23
Figure S35: IR spectrum of **12** (film, KBr disc) 24
Figure S36: UV/vis (DAD) spectrum of **12** (MeOH) 24
Figure S37: ^{1}H NMR spectrum (500 MHz, $CDCl_3$) of **12**. 25
Figure S38: ^{13}C NMR spectrum (125 MHz, $CDCl_3$) of **12**. 25
Figure S39: COSY spectrum of **12**. 26
Figure S40: HSQC spectrum of **12**. 26
Figure S41: HMBC spectrum of **12**. 27
Figure S42: NOESY spectrum **12**. 27
Figure S43: (+)ESI-MS spectrum of **13**. 28
Figure S44: HRESIMS spectrum of **13**. 28
Figure S45: IR spectrum of **13** (film, KBr disc). 29
Figure S46: UV/vis (DAD) spectrum of **13** (MeOH) 29
Figure S47: ^{1}H NMR spectrum (500 MHz, $CDCl_3$) of **13**. 30
Figure S48: ^{13}C NMR spectrum (125 MHz, $CDCl_3$) of **13**. 30
Figure S49: COSY spectrum of **13**. 31
Figure S50: HSQC spectrum of **13**. 31
Figure S51: HMBC spectrum of **13**. 32
Figure S52: NOESY spectrum **13**. 32
Figure S53: (+)ESI-MS spectrum of **14**. 33
Figure S54: HRESIMS spectrum of **14**. 33
Figure S55: IR spectrum of **14** (film, KBr disc). 34
Figure S56: UV/vis (DAD) spectrum of **14** (MeOH). 34
Figure S57: ^{1}H NMR spectrum (500 MHz, $CDCl_3$) of **14**. 35
Figure S58: ^{13}C NMR spectrum (125 MHz, $CDCl_3$) of **14**. 35
Figure S59: HSQC spectrum of **14**. 36
Figure S60: HMBC spectrum of **14**. 36
Figure S61: (+)ESI-MS spectrum of **15**. 37
Figure S62: HRESIMS spectrum of **15**. 37
Figure S63: IR spectrum of **15** (film, KBr disc) 38
Figure S64: UV/vis (DAD) spectrum of **15** (MeOH). 38

Figure S65: ^{1}H NMR spectrum (500 MHz, $CDCl_3$) of **15**. 39
Figure S66: ^{13}C NMR spectrum (125 MHz, $CDCl_3$) of **15**. 39
Figure S67: HSQC spectrum of **15**. 40
Figure S68: HMBC spectrum of **15**. 40
Figure S69: (+)ESI-MS spectrum of **16**. 41
Figure S70: HRESIMS spectrum of **16**. 41
Figure S71: IR spectrum of **16** (film, KBr disc) 42
Figure S72: UV/vis (DAD) spectrum of **16** (MeOH) 42
Figure S73: ^{1}H NMR spectrum (500 MHz, $CDCl_3$) of **16**. 43
Figure S74: ^{13}C NMR spectrum (125 MHz, $CDCl_3$) of **16**. 43
Figure S75: COSY spectrum of **16**. 44
Figure S76: HSQC spectrum of **16**. 44
Figure S77: HMBC spectrum of **16**. 45
Figure S78: NOESY spectrum **16**. 45
Figure S79: (-)ESI-MS spectrum of **17**. 46
Figure S80: HRESIMS spectrum of **17**. 46
Figure S81: IR spectrum of **17** (film, KBr disc). 47
Figure S82: UV/vis (DAD) spectrum of **17** (MeOH) 47
Figure S83: ^{1}H NMR spectrum (500 MHz, $CDCl_3$) of **17**. 48
Figure S84: ^{13}C NMR spectrum (125 MHz, $CDCl_3$) of **17**. 48
Figure S85: COSY spectrum of **17**. 49
Figure S86: HSQC spectrum of **17**. 49
Figure S87: HMBC spectrum of **17**. 50
Figure S88: NOESY spectrum **17**. 50
Figure S89: (+)ESI-MS spectrum of **18**. 51
Figure S90: HRESIMS spectrum of **18**. 51
Figure S91: IR spectrum of **18** (film, KBr disc) 52
Figure S92: UV/vis (DAD) spectrum of **18** (MeOH). 52
Figure S93: ^{1}H NMR spectrum (500 MHz, $CDCl_3$) of **18**. 53
Figure S94: ^{13}C NMR spectrum (125 MHz, $CDCl_3$) of **18**. 53
Figure S95: COSY spectrum of **18**. 54
Figure S96: HSQC spectrum of **18**. 54
Figure S97: HMBC spectrum of **18**. 55

Figure S98: NOESY spectrum **18**....55
Figure S99: (-)ESI-MS spectrum of **19**....56
Figure S100: HRESIMS spectrum of **19**. 56
Figure S101: IR spectrum of **19** (film, KBr disc)....57
Figure S102: UV/vis (DAD) spectrum of **19** (MeOH)....57
Figure S103: ^{1}H NMR spectrum (500 MHz, $CDCl_3$) of **19**....58
Figure S104: ^{13}C NMR spectrum (125 MHz, $CDCl_3$) of **19**....58
Figure S105: COSY spectrum of **19**....59
Figure S106: HSQC spectrum of **19**....59
Figure S107: HMBC spectrum of **19**....60
Figure S108: NOESY spectrum **19**....60
Figure S109: (+)ESI-MS spectrum of **20**....61
Figure S110: HRESIMS spectrum of **20**. 61
Figure S111: IR spectrum of **20** (film, KBr disc)....62
Figure S112: UV/vis (DAD) spectrum of **20** (MeOH)....62
Figure S113: ^{1}H NMR spectrum (500 MHz, $CDCl_3$) of **20**....63
Figure S114: ^{13}C NMR spectrum (125 MHz, $CDCl_3$) of **20**....63
Figure S115: COSY spectrum of **20**....64
Figure S116: HSQC spectrum of **20**....64
Figure S117: HMBC spectrum of **20**....65
Figure S118: NOESY spectrum **20**....65
Figure S119: Key NOESY correlations (indicated by blue arrows) for compounds **13**, **17-20**....66
Table S1: The minimum inhibitory concentration (MIC) of tested compounds against Gram-positive bacterium *Bacillus subtilis*....66

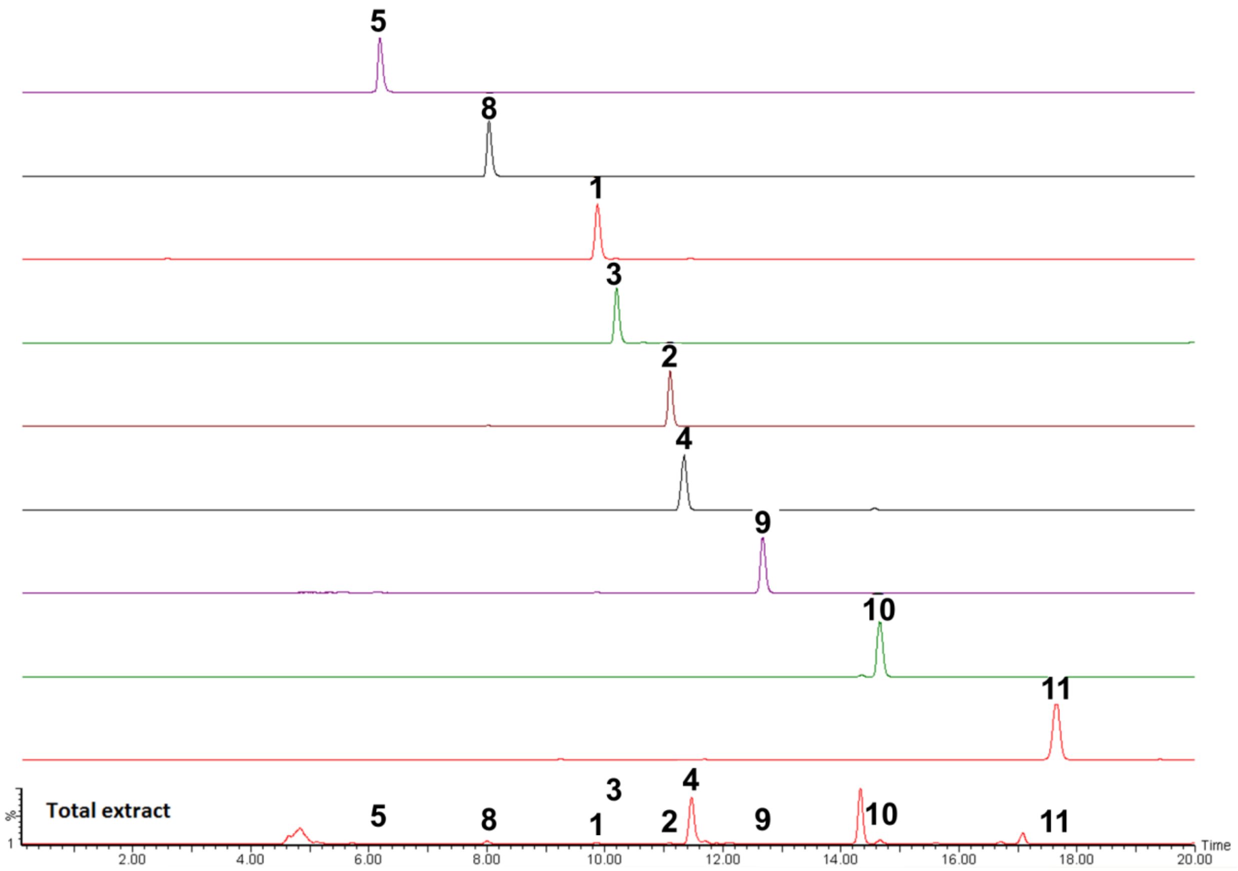

Figure S1: HPLC profiles (ELSD) for cladobotric acids **1-5**, **8-11**, which were isolated from a crude extract of *Cladobotryum* sp. (bottom trace).

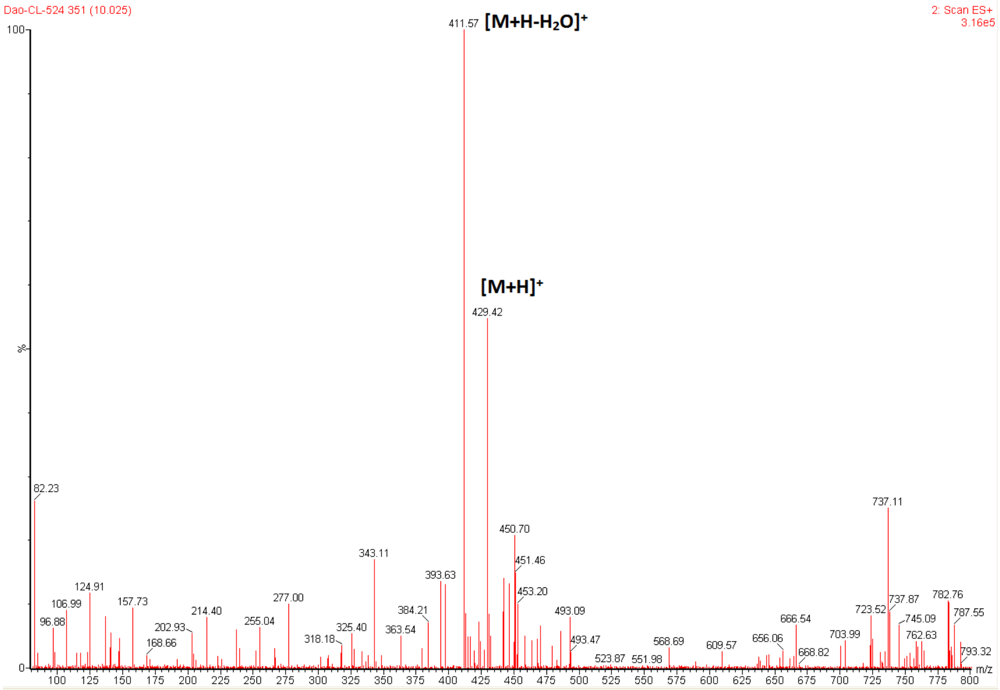

Figure S2: (+)ESI-MS spectrum of **1**.

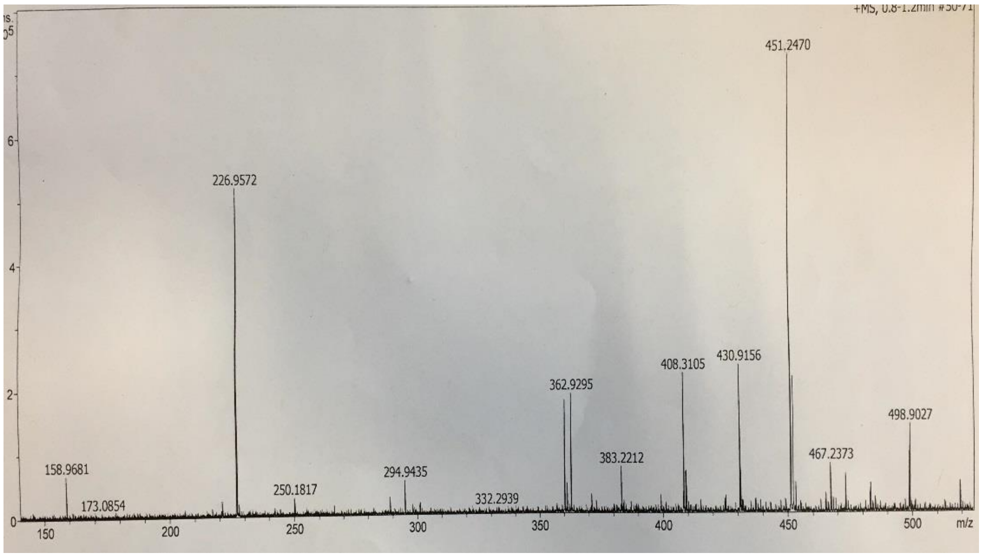

Figure S3: HRESIMS spectrum of **1**.

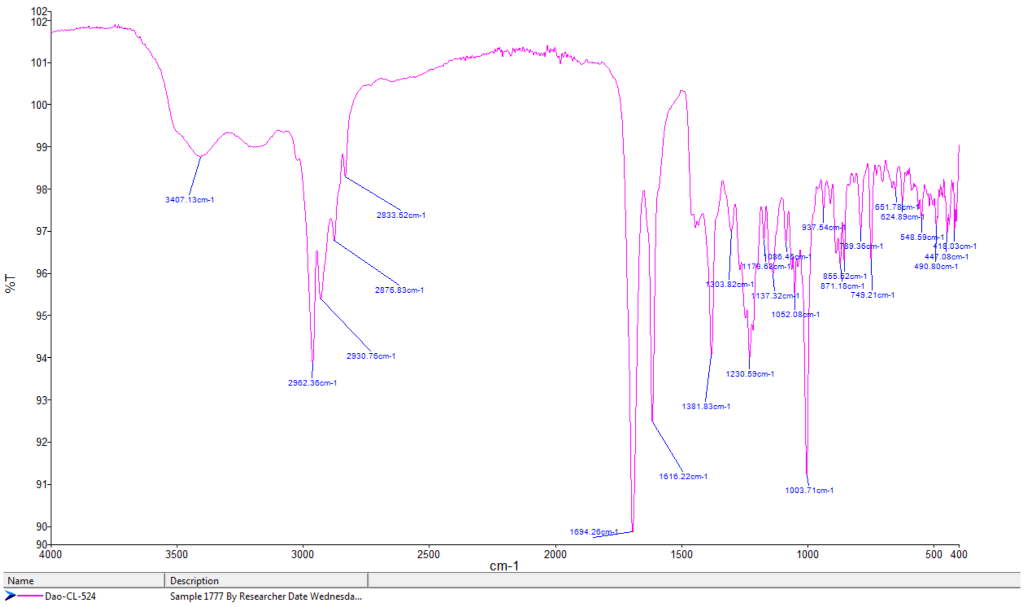

Figure S4: IR spectrum of **1** (film, KBr disc)

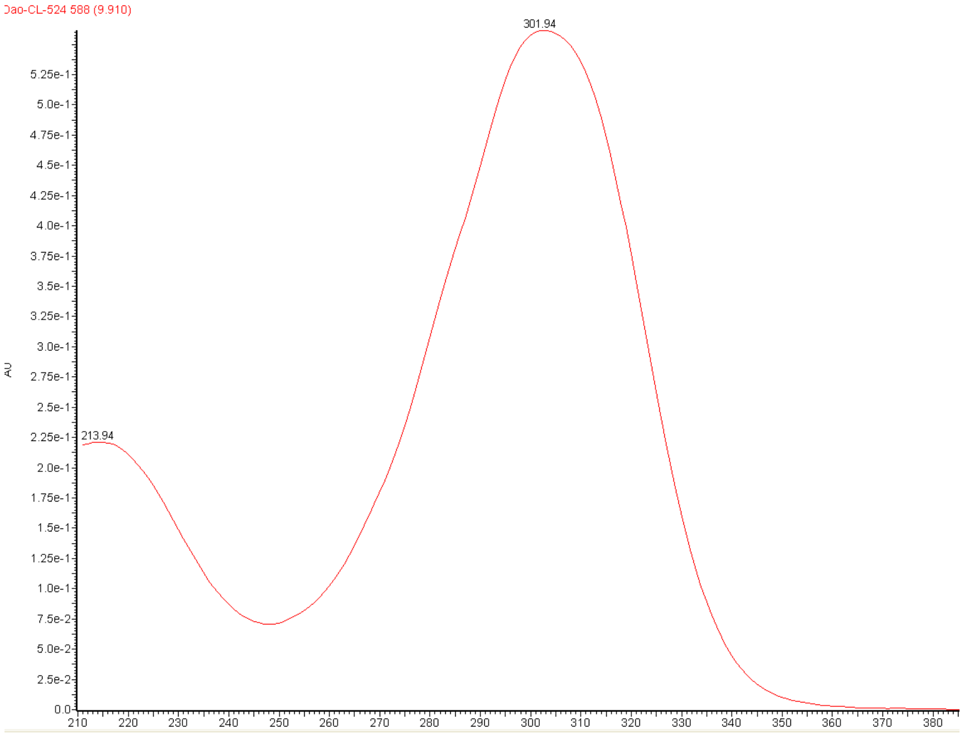

Figure S5: UV/vis (DAD) spectrum of **1** (in MeOH)

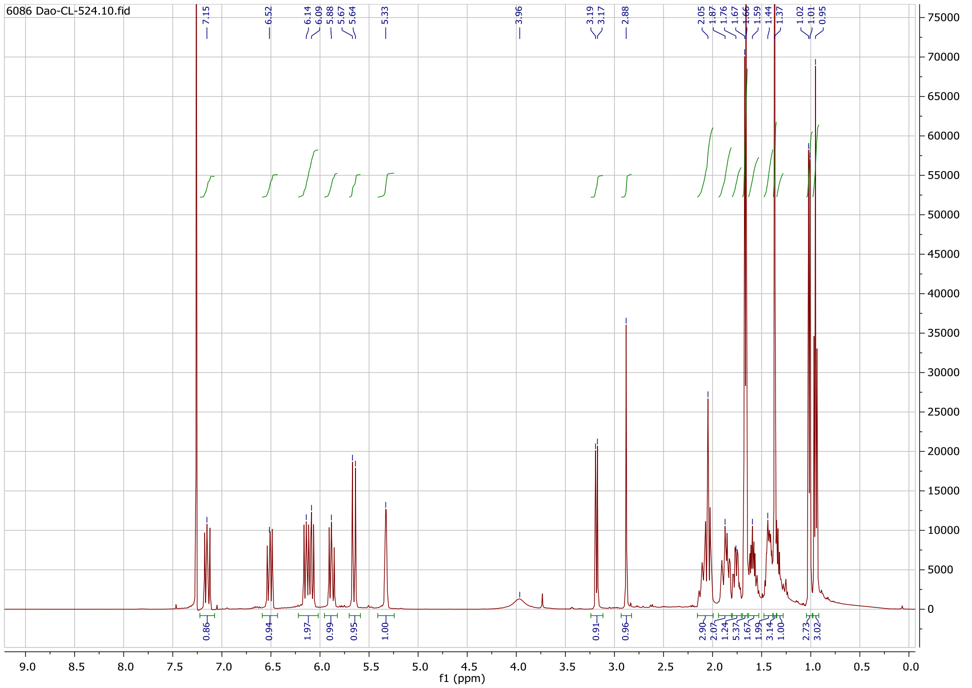

Figure S6: ^{1}H NMR spectrum (500 MHz, $CDCl_3$) of **1**.

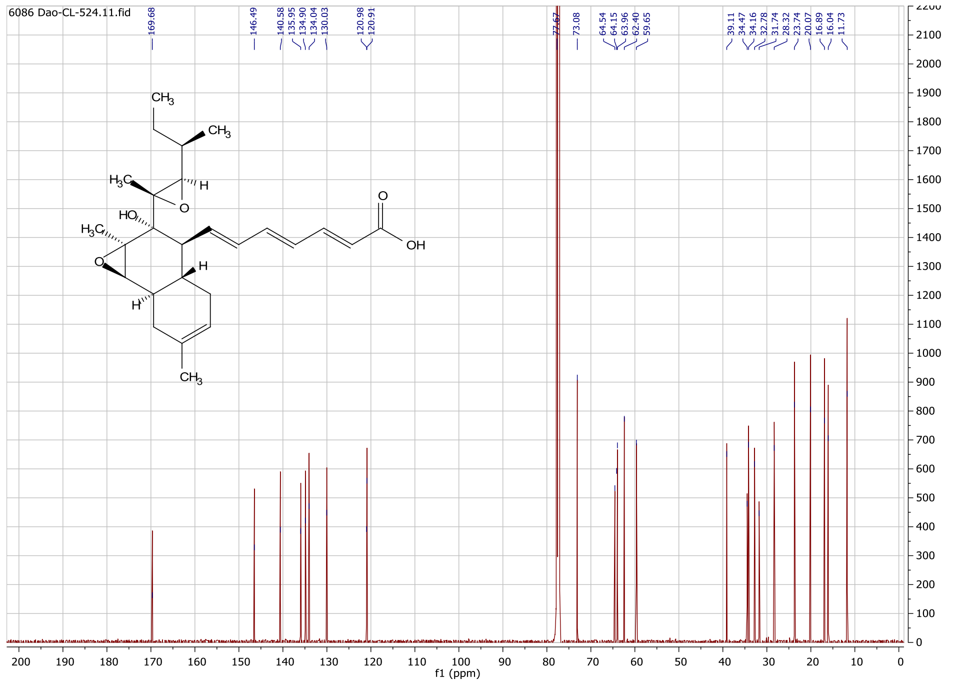

Figure S7: ^{13}C NMR spectrum (125 MHz, $CDCl_3$) of **1**.

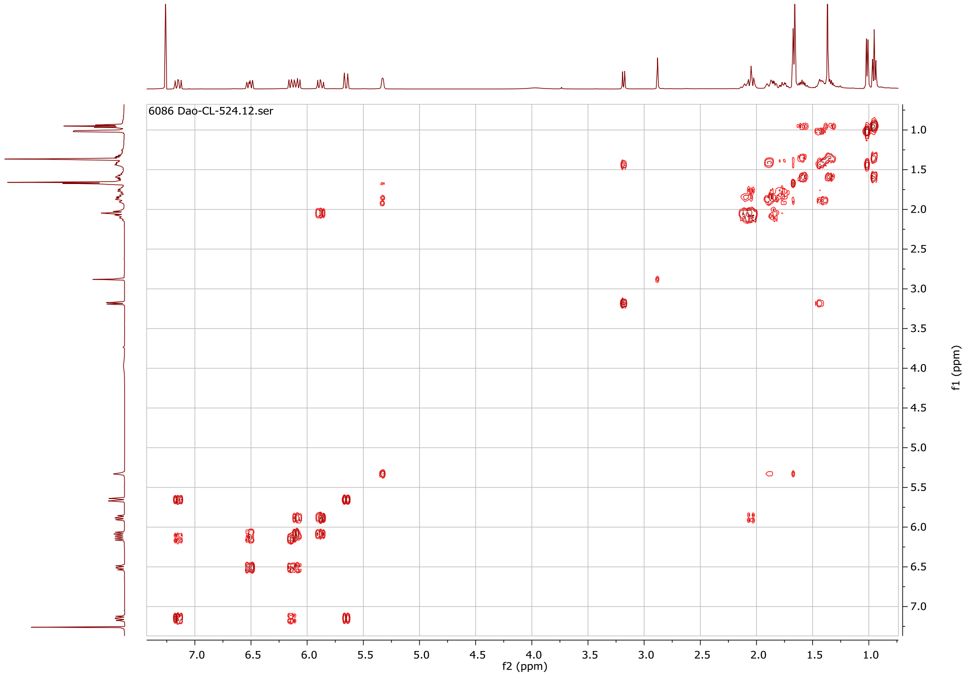

Figure S8: COSY spectrum of **1**.

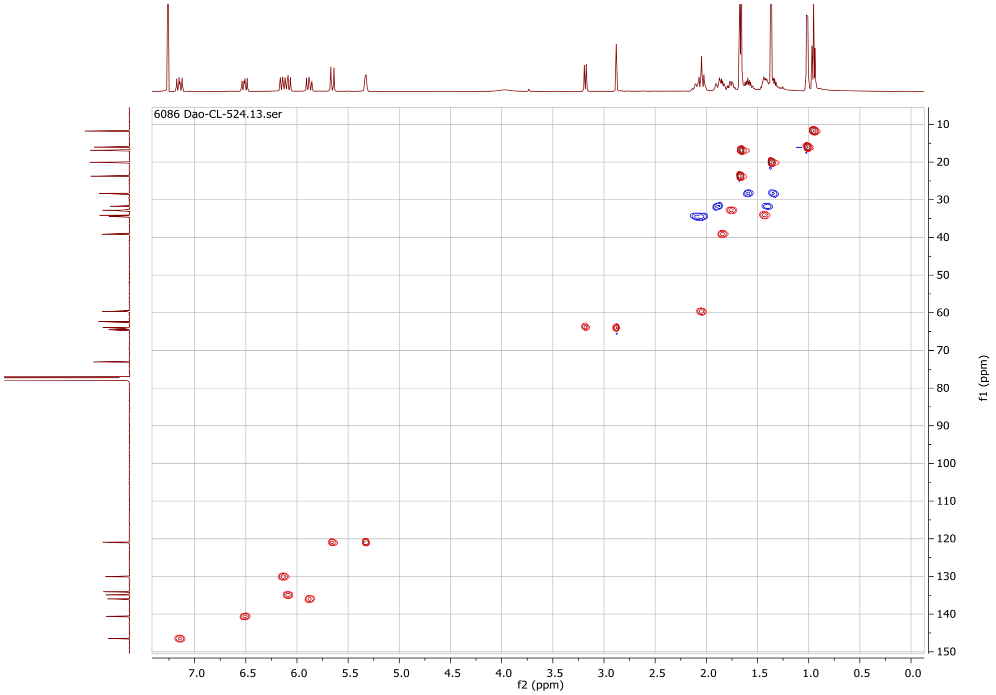

Figure S9: HSQC spectrum of **1**.

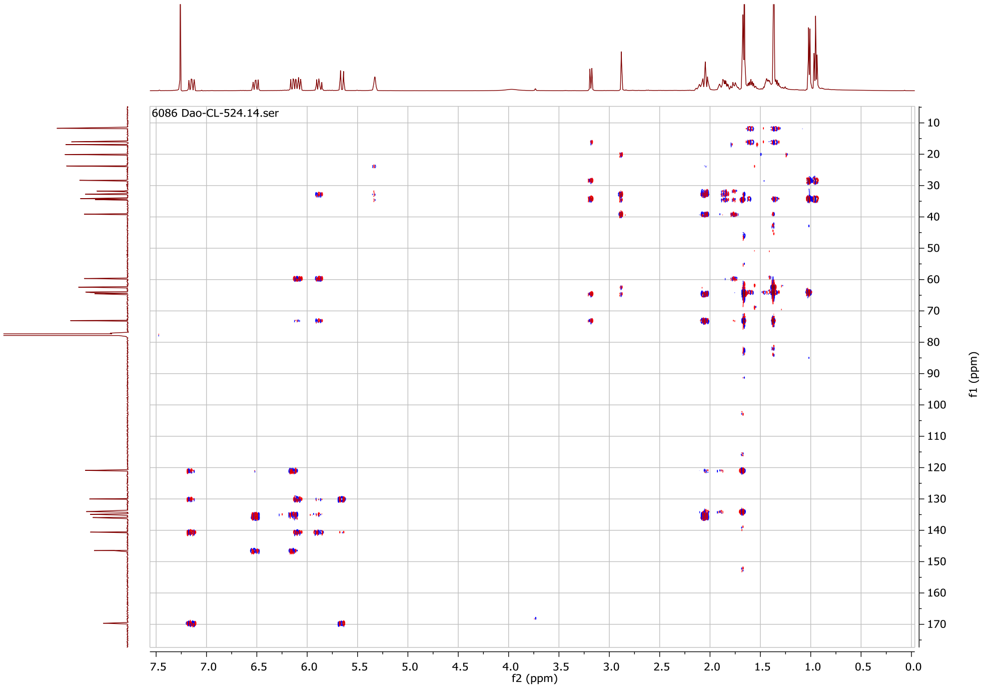

Figure S10: HMBC spectrum of **1**.

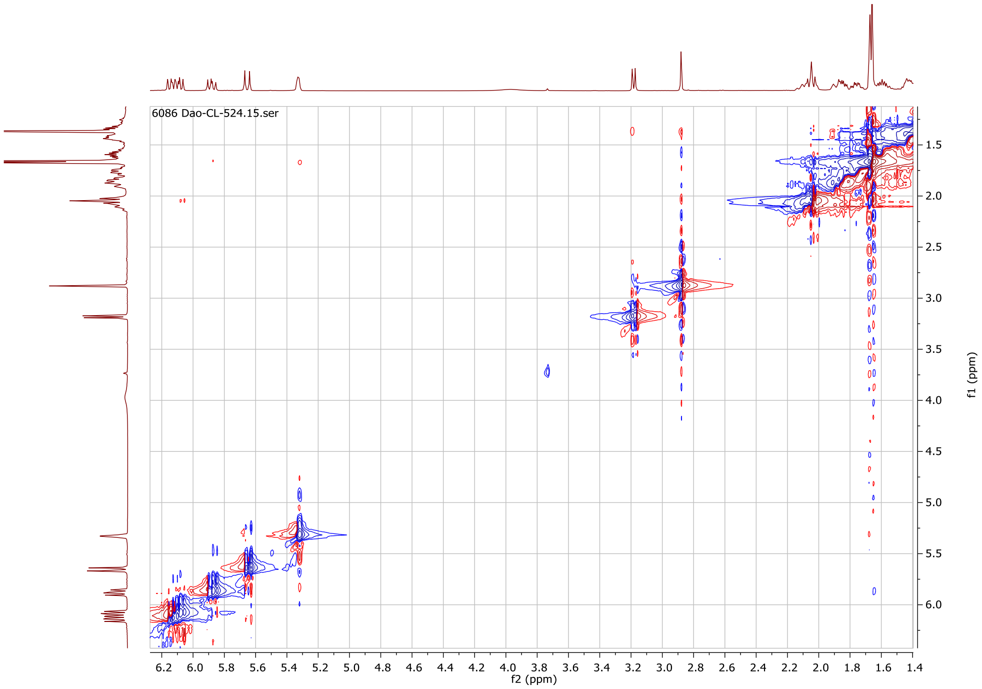

Figure S11: NOESY spectrum **1**.

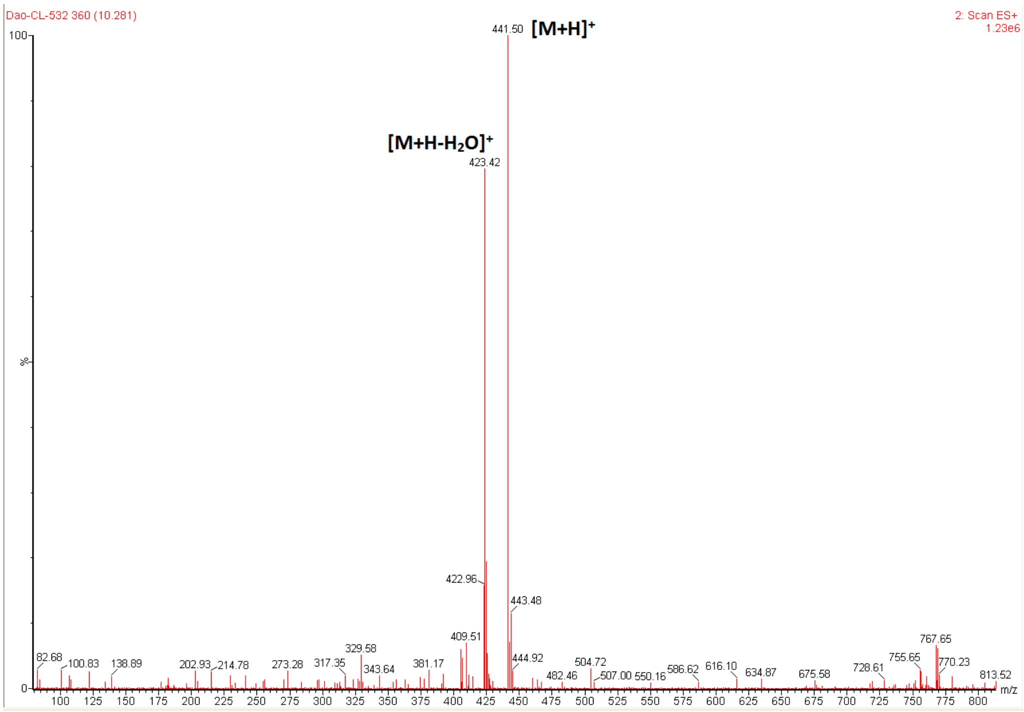

Figure S12: (+)ESI-MS spectrum of **2**.

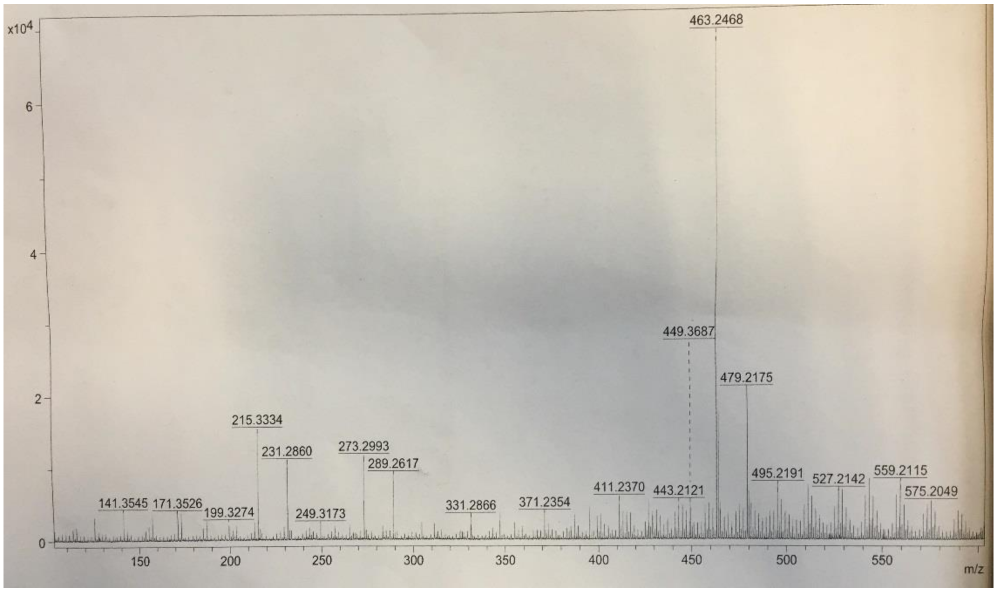

Figure S13: HRESIMS spectrum of **2**.

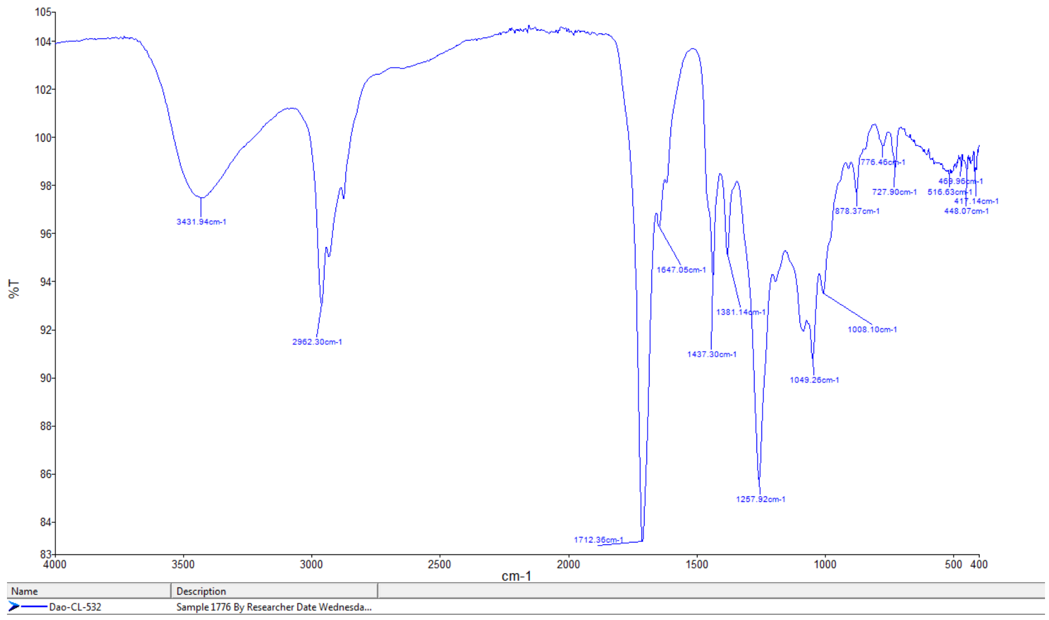

Figure S14: IR spectrum of **2** (film, KBr disc)

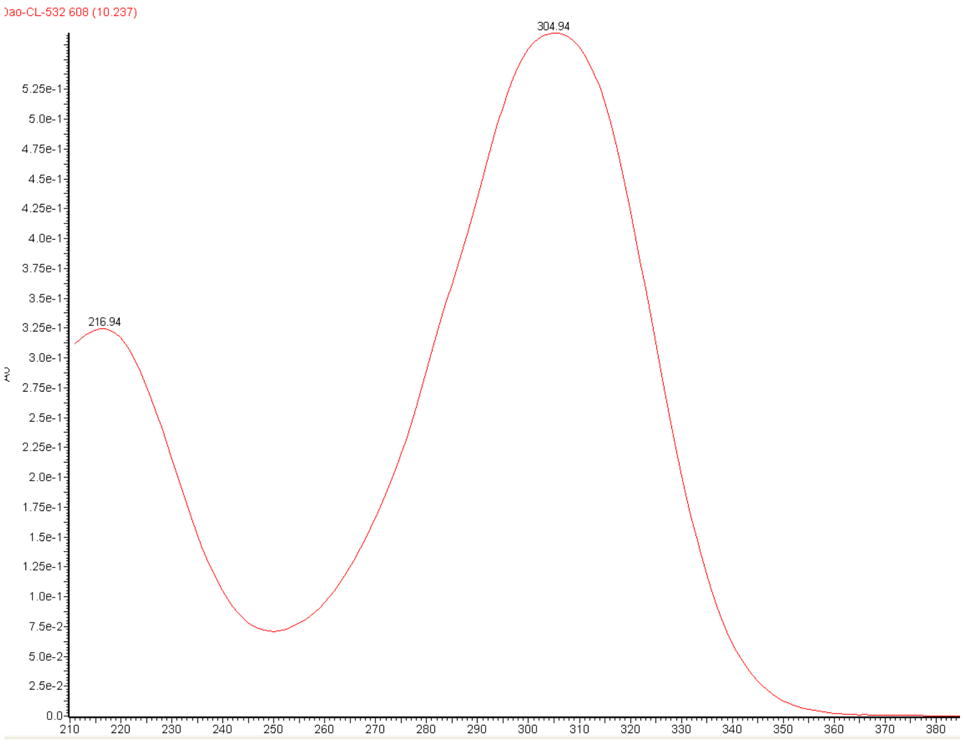

Figure S15: UV/vis (DAD) spectrum of **2** (in MeOH)

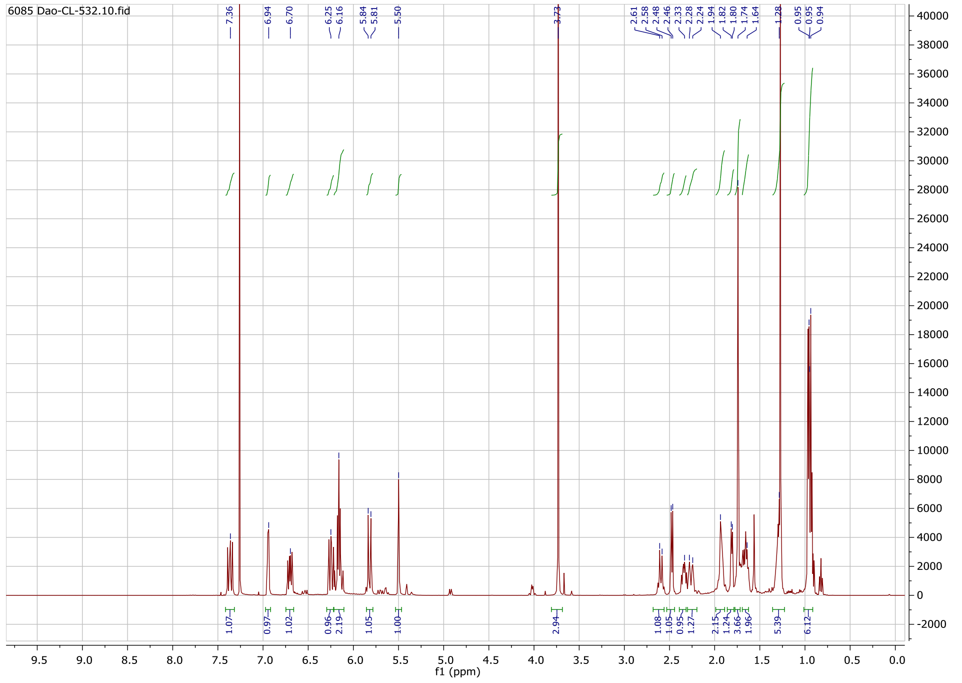

Figure S16: ^{1}H NMR spectrum (500 MHz, $CDCl_3$) of **2**.

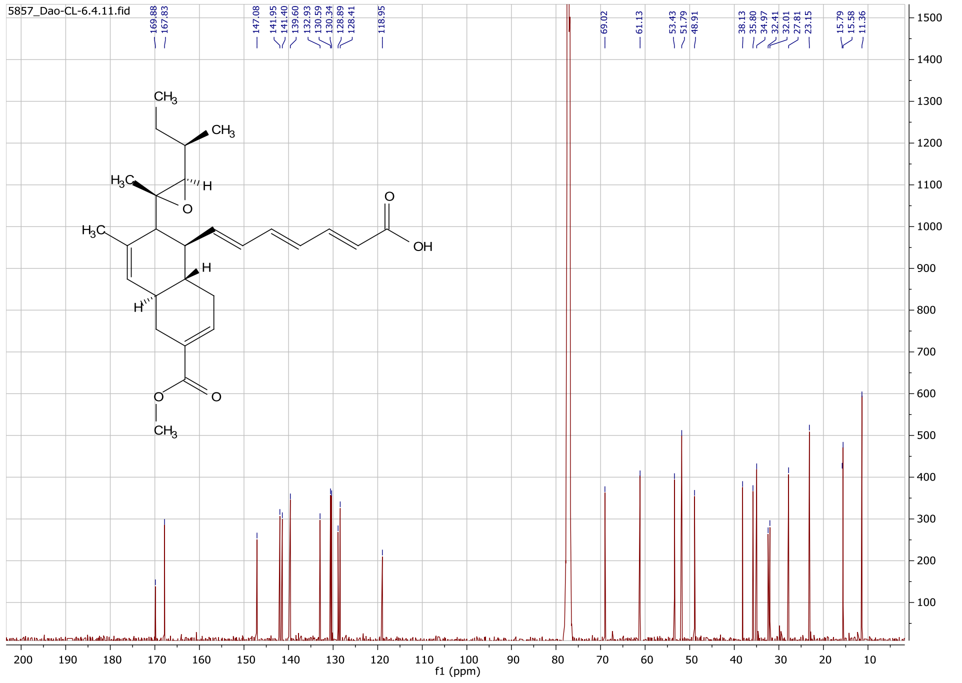

Figure S17: ^{13}C NMR spectrum (125 MHz, $CDCl_3$) of **2**.

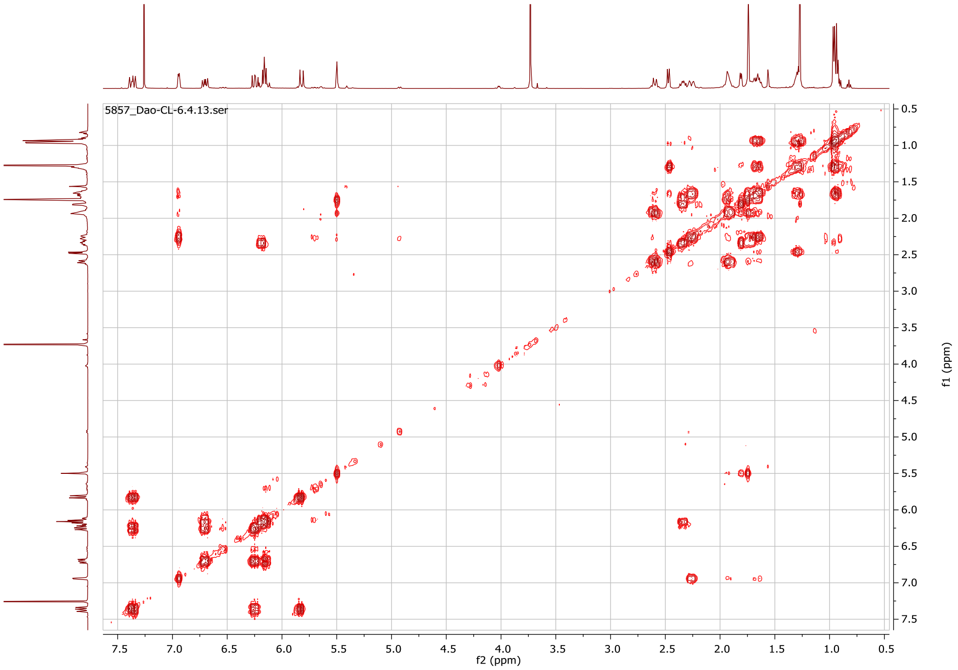

Figure S18: COSY spectrum of **2**.

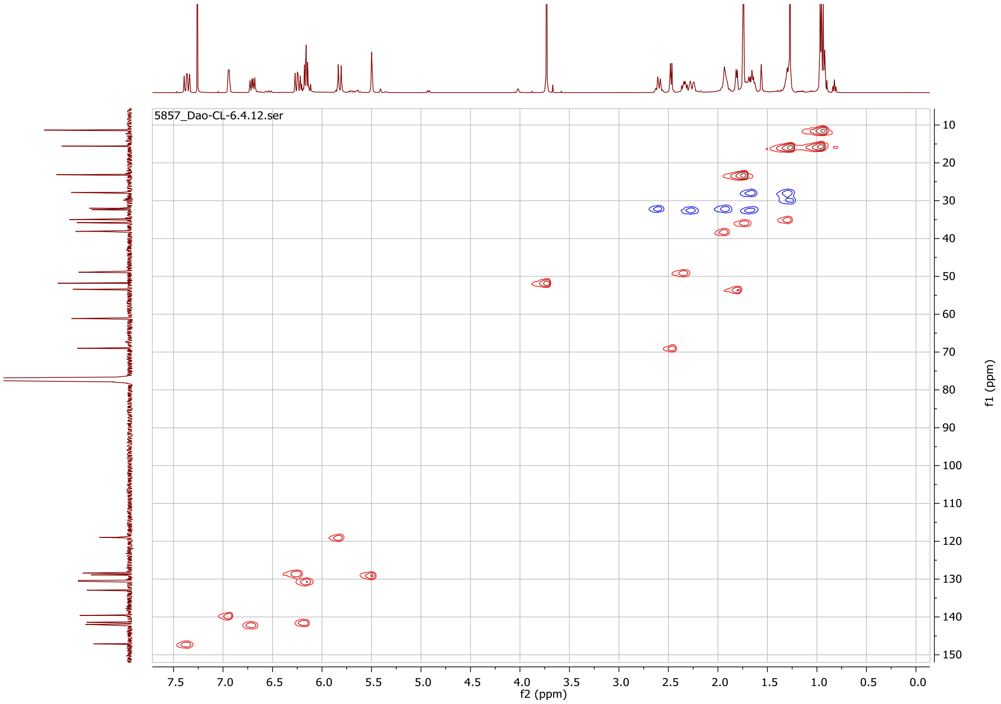

Figure S19: HSQC spectrum of **2**.

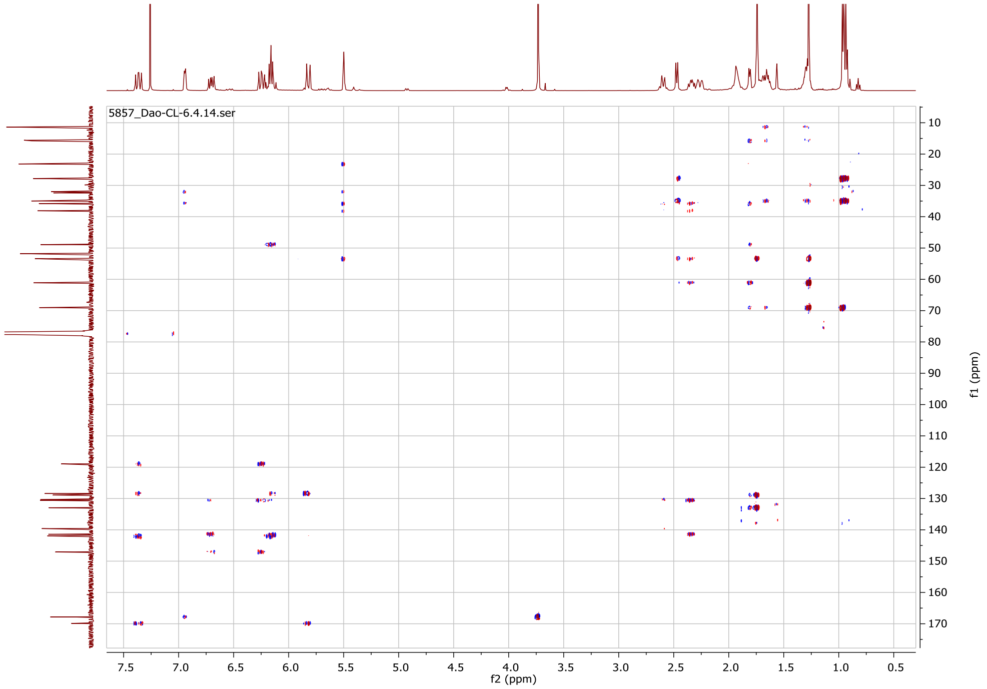

Figure S20: HMBC spectrum of **2**.

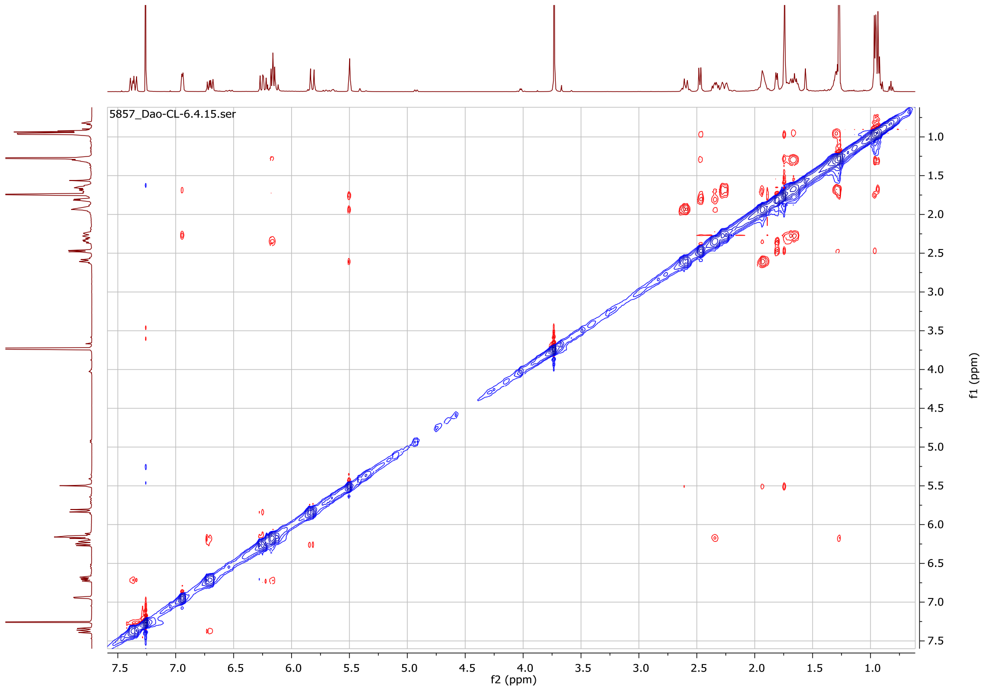

Figure S21: NOESY spectrum **2**.

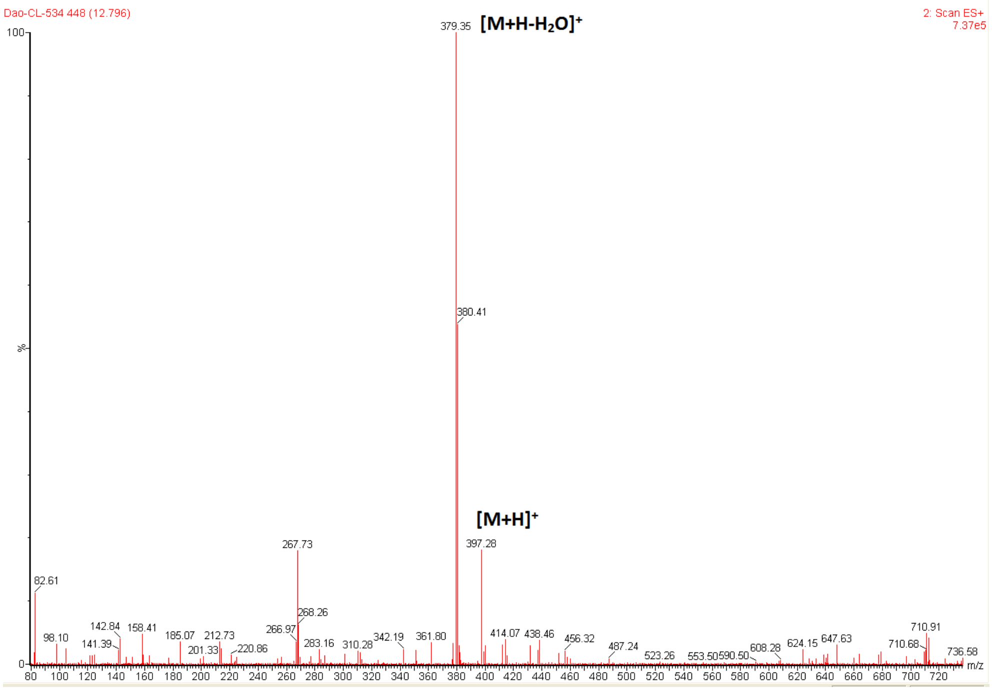

Figure S22: (+)ESI-MS spectrum of **3**.

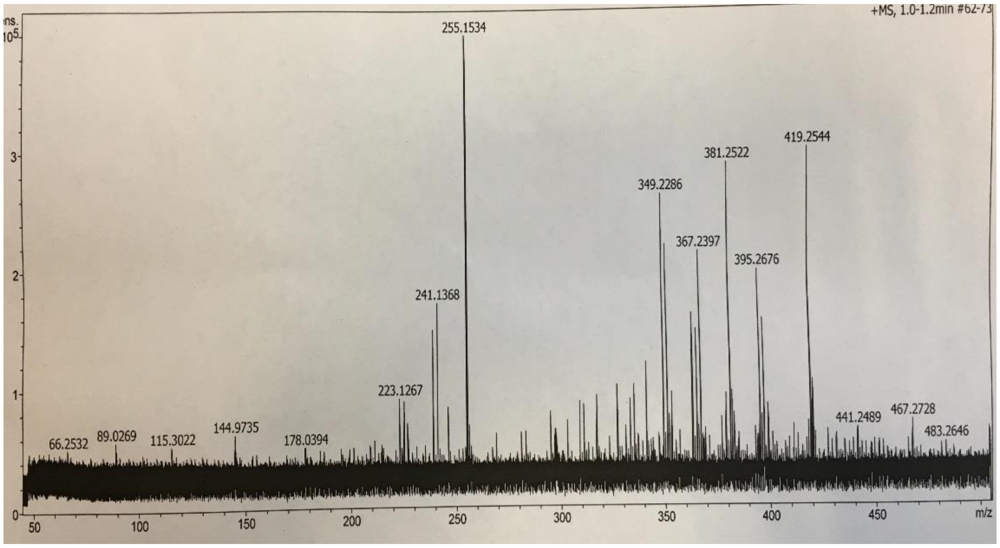

Figure S23: HRESIMS spectrum of **3**.

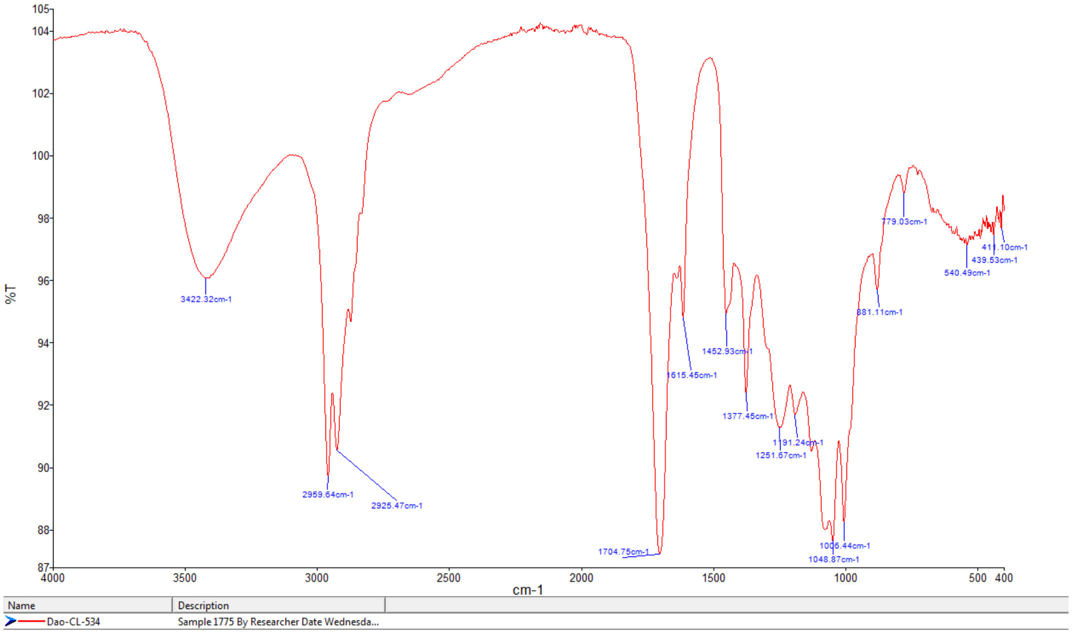

Figure S24: IR spectrum of **3** (film, KBr disc)

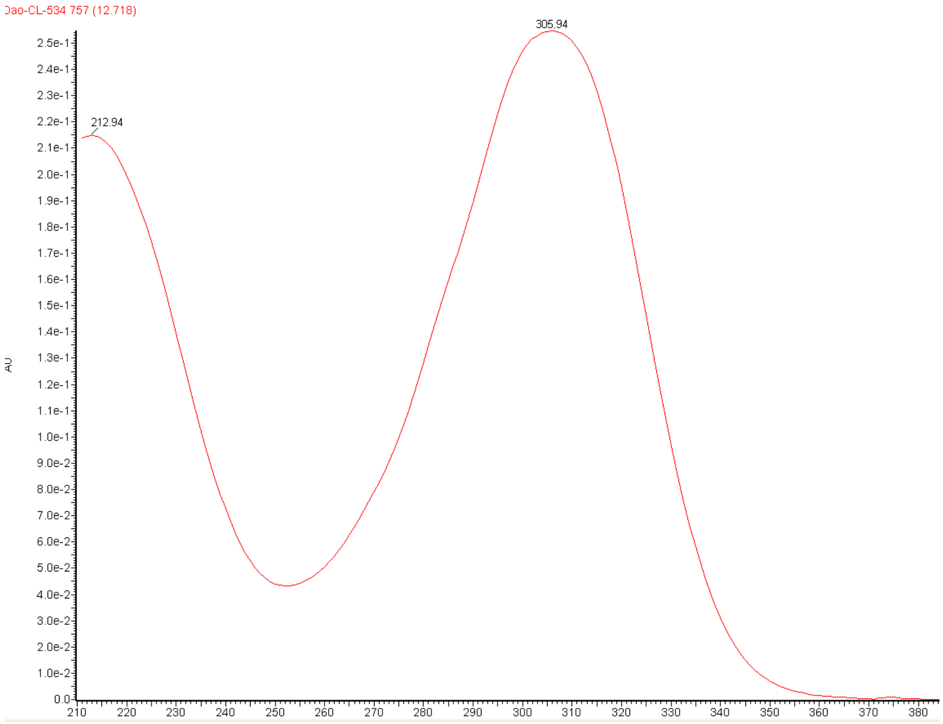

Figure S25: UV/vis (DAD) spectrum of **3**. (MeOH)

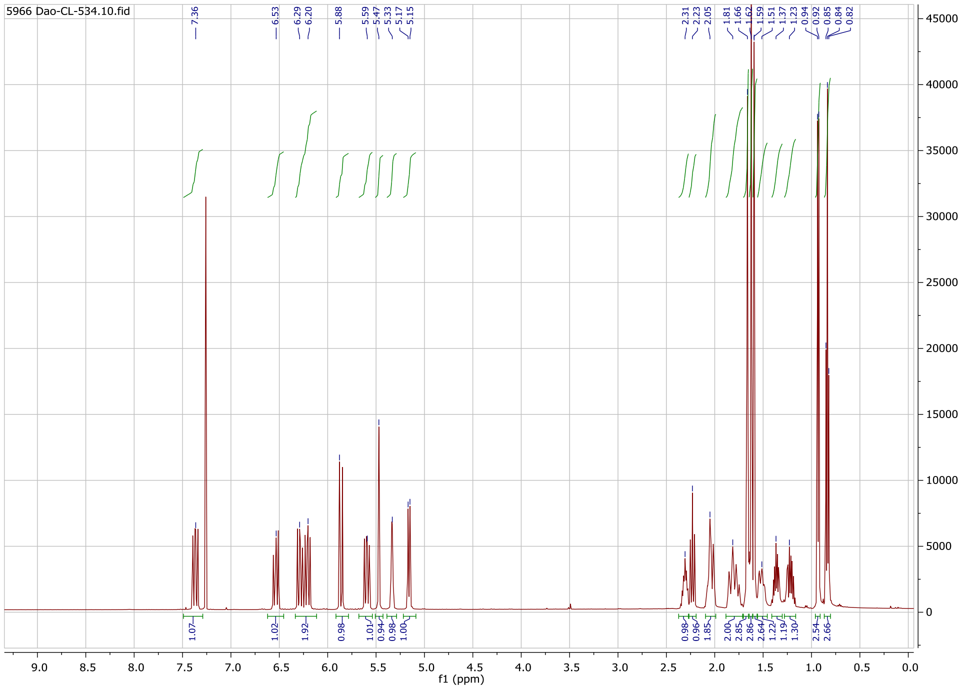

Figure S26: ^{1}H NMR spectrum (500 MHz, $CDCl_3$) of **3**.

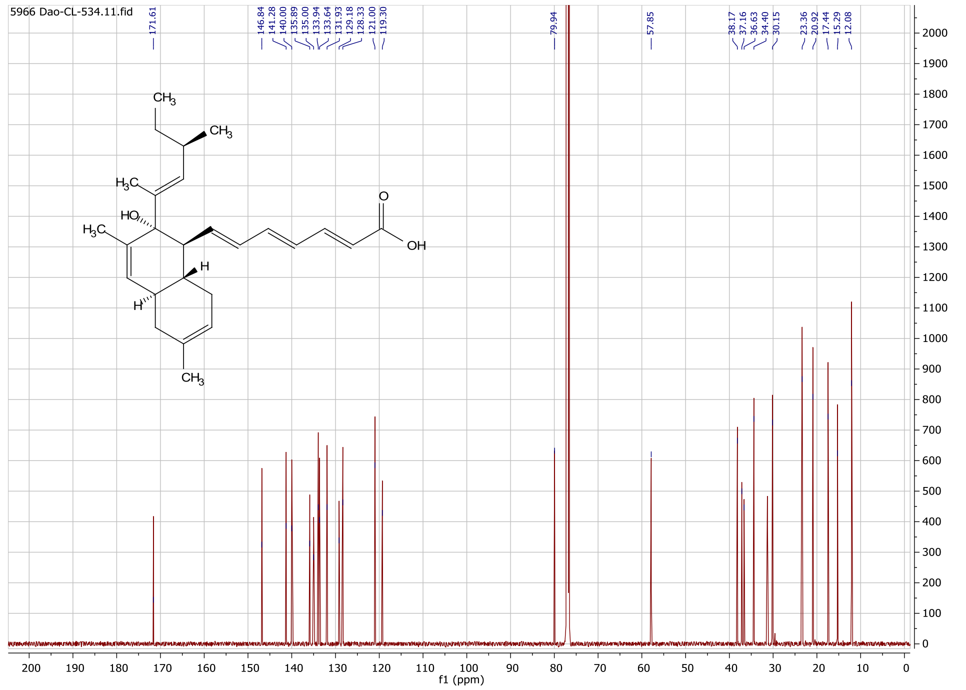

Figure S27: ^{13}C NMR spectrum (125 MHz, $CDCl_3$) of **3**.

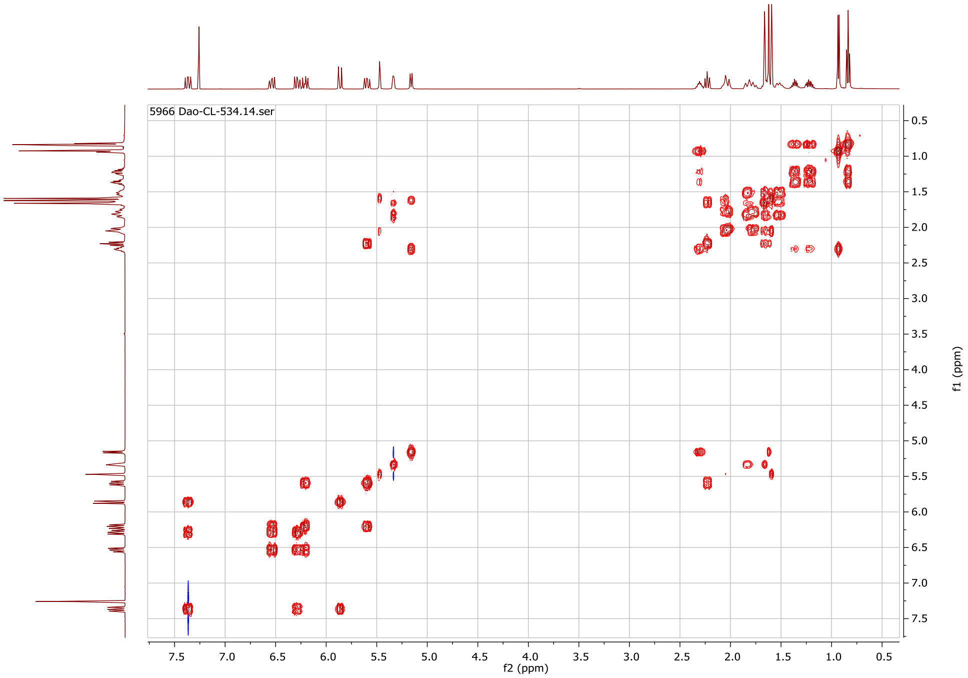

Figure S28: COSY spectrum of **3**.

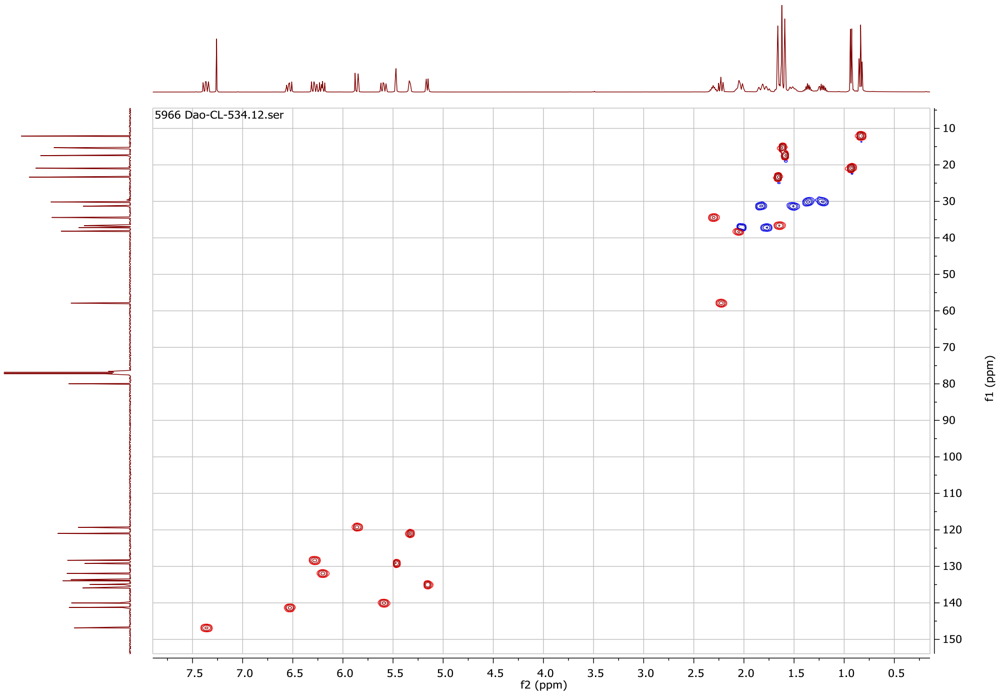

Figure S29: HSQC spectrum of **3**.

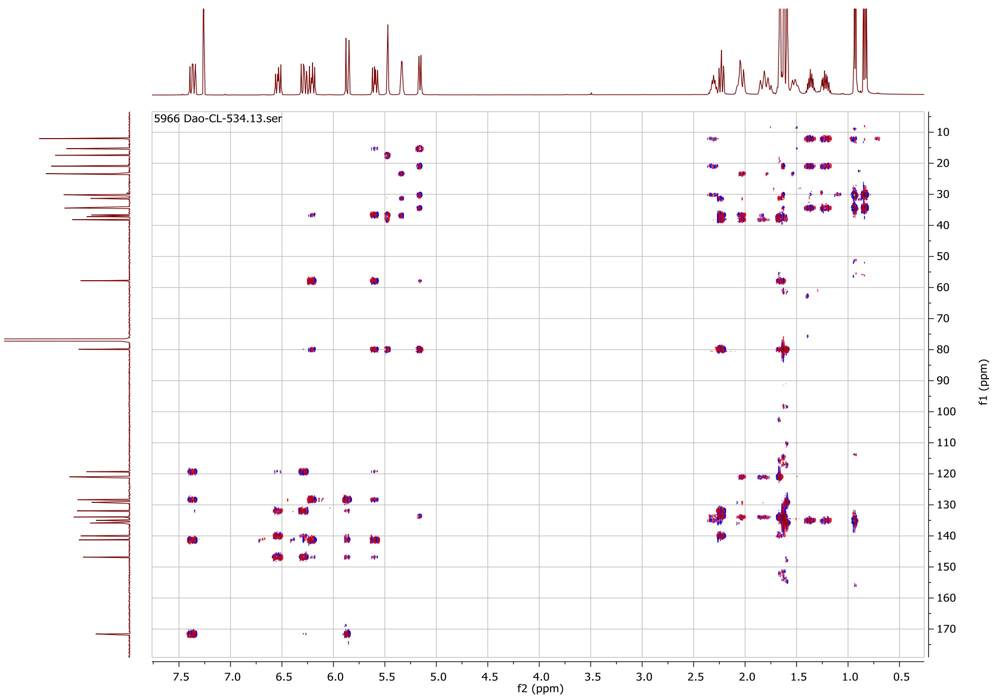

Figure S30: HMBC spectrum of **3**.

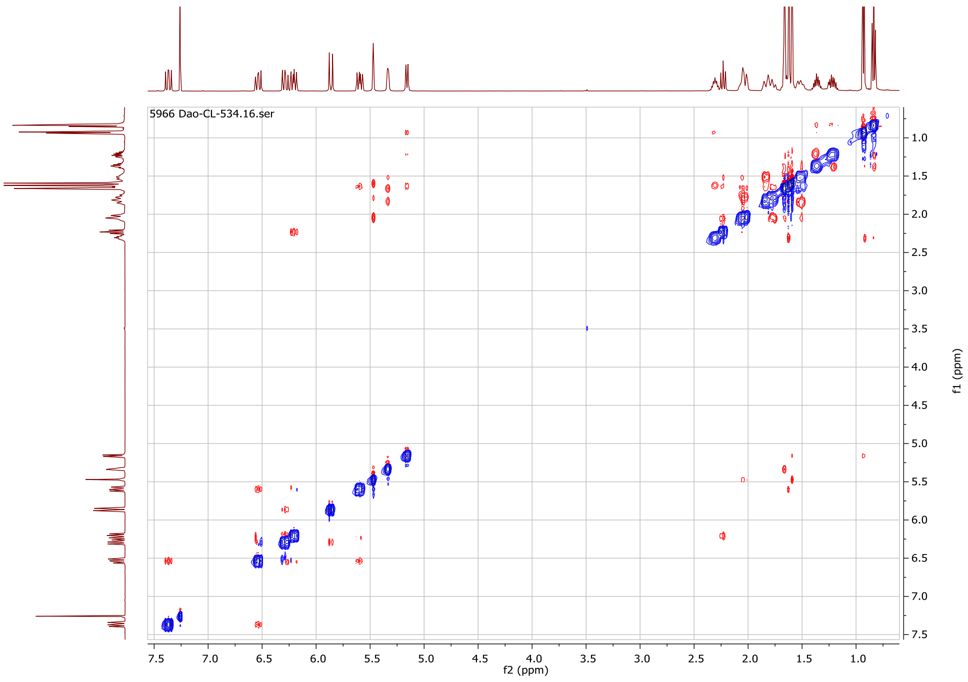

Figure S31: NOESY spectrum **3**.

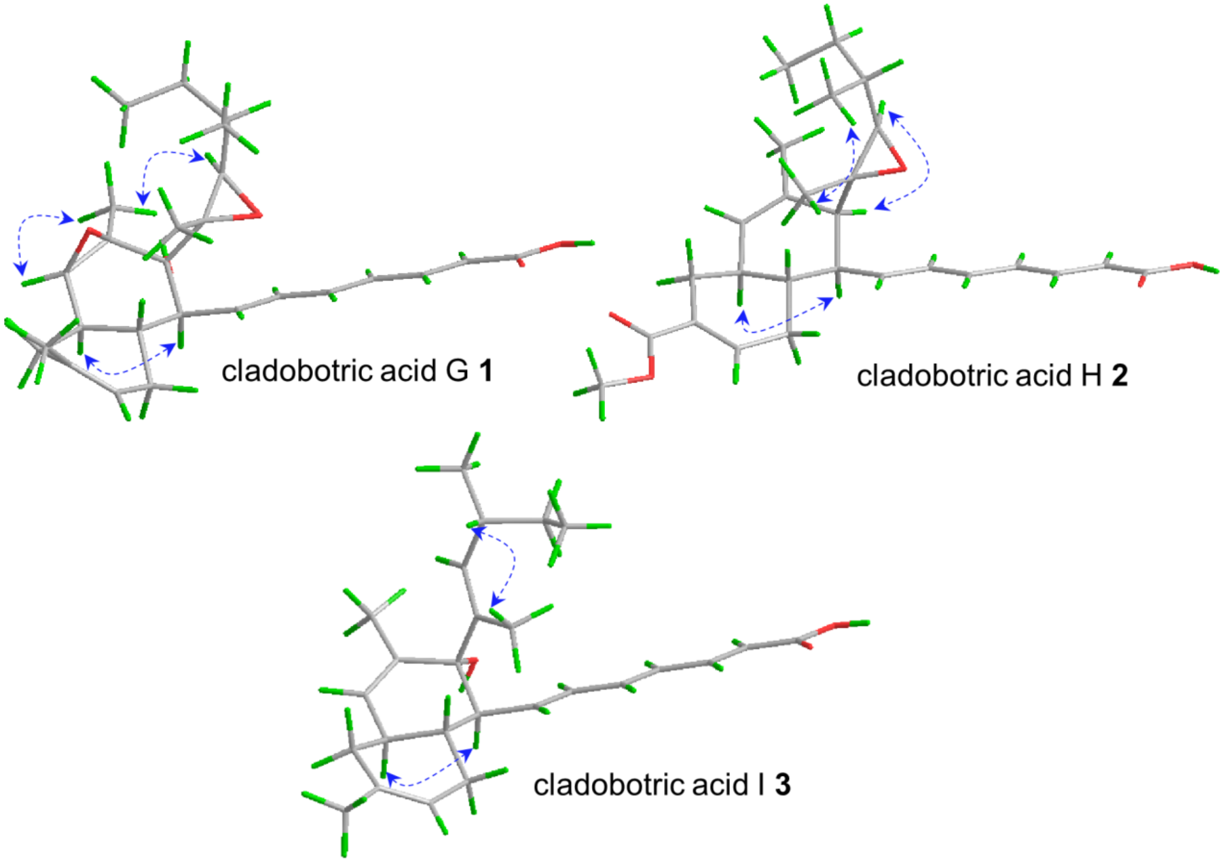

Figure S32: Key NOESY correlations (indicated by blue arrows) for compounds **1**–**3**

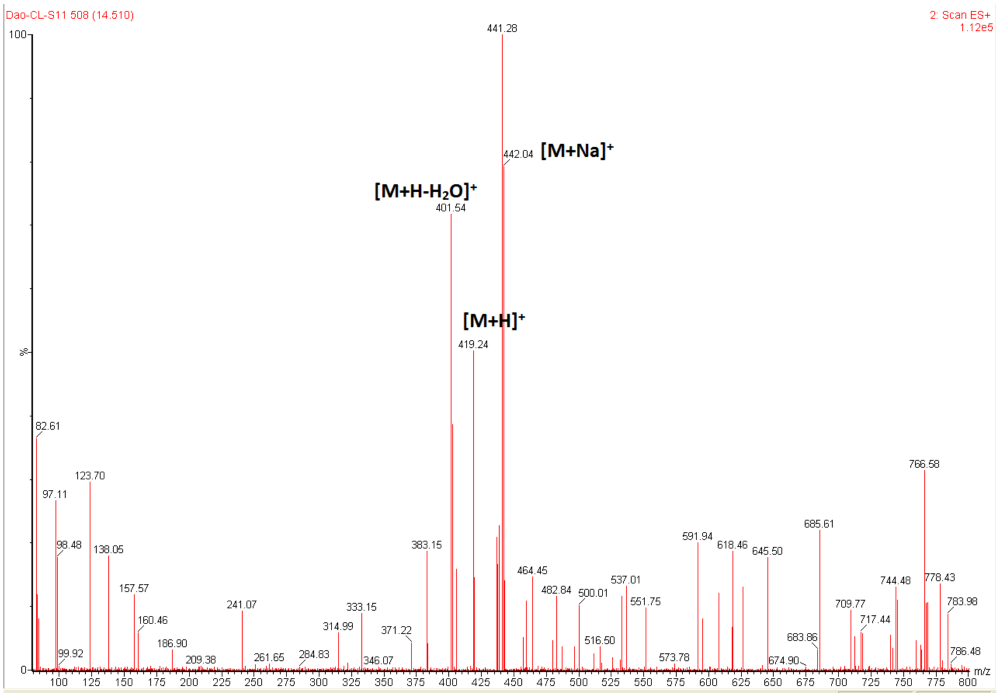

Figure S33: (+)ESI-MS spectrum of **12**.

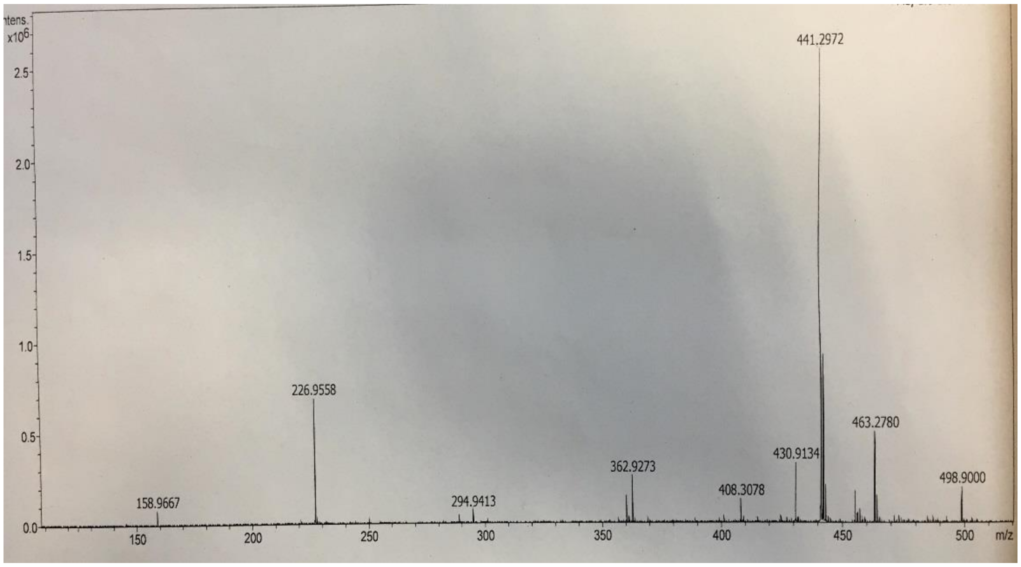

Figure S34: HRESIMS spectrum of **12**.

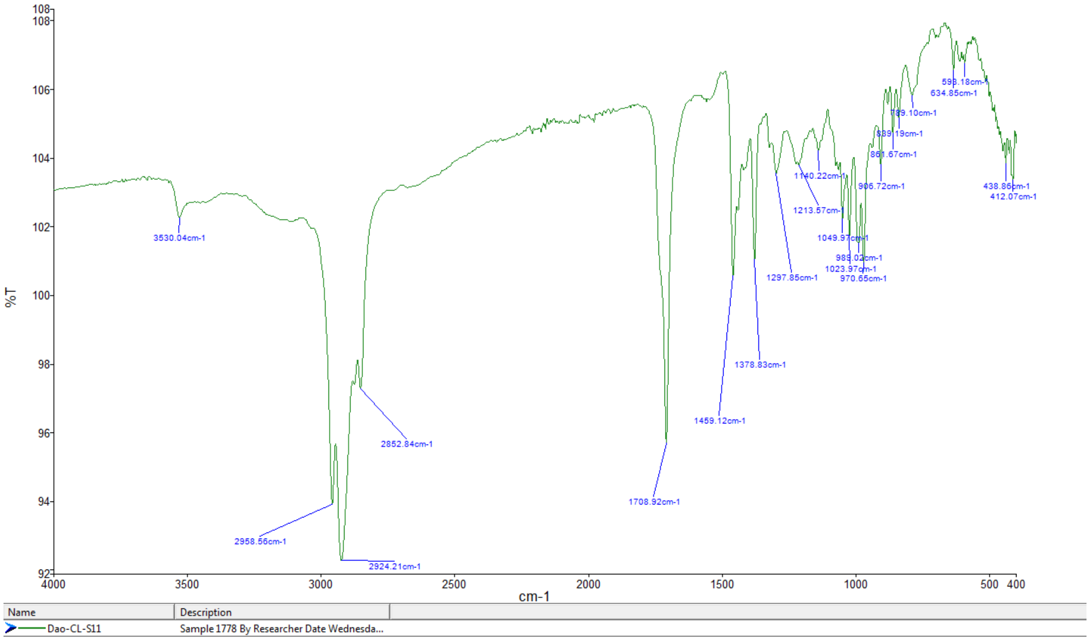

Figure S35: IR spectrum of **12** (film, KBr disc)

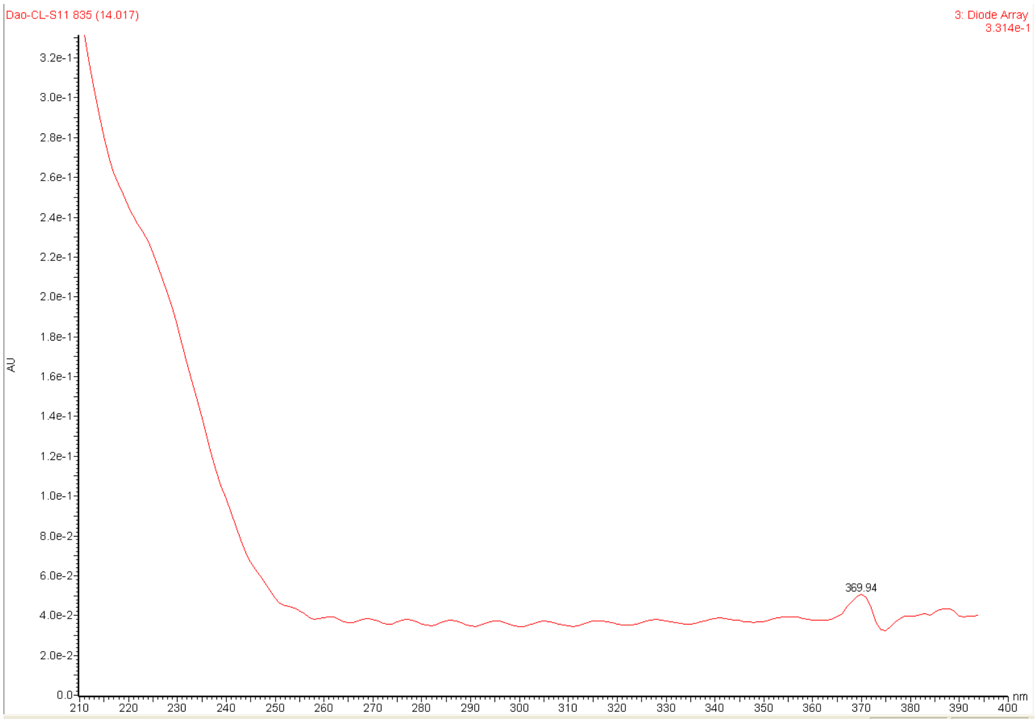

Figure S36: UV/vis (DAD) spectrum of **12** (MeOH)

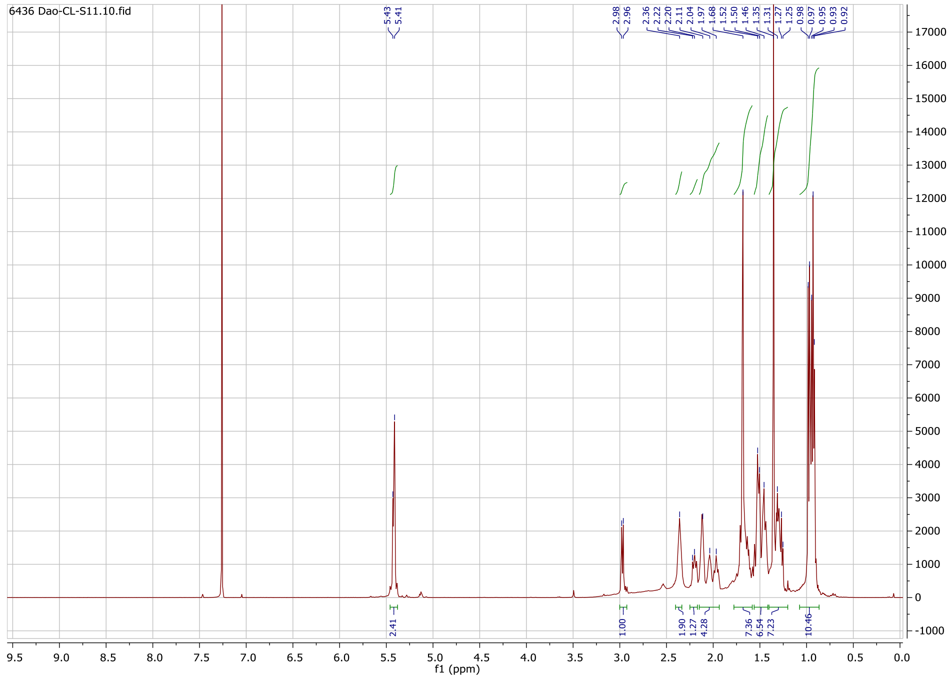

Figure S37: ^{1}H NMR spectrum (500 MHz, $CDCl_3$) of **12**.

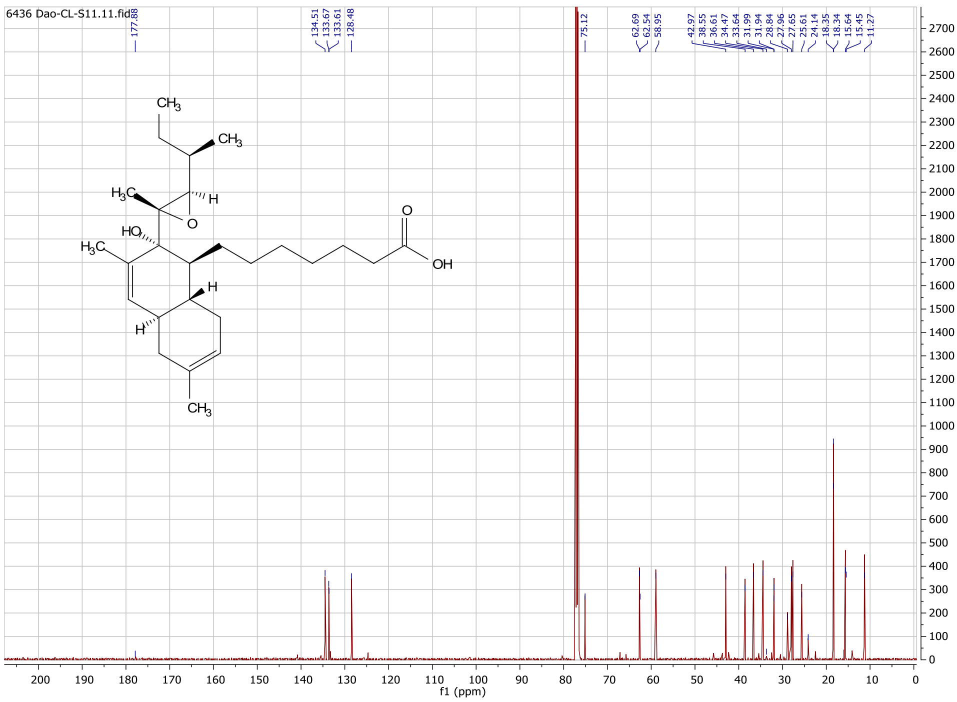

Figure S38: ^{13}C NMR spectrum (125 MHz, $CDCl_3$) of **12**.

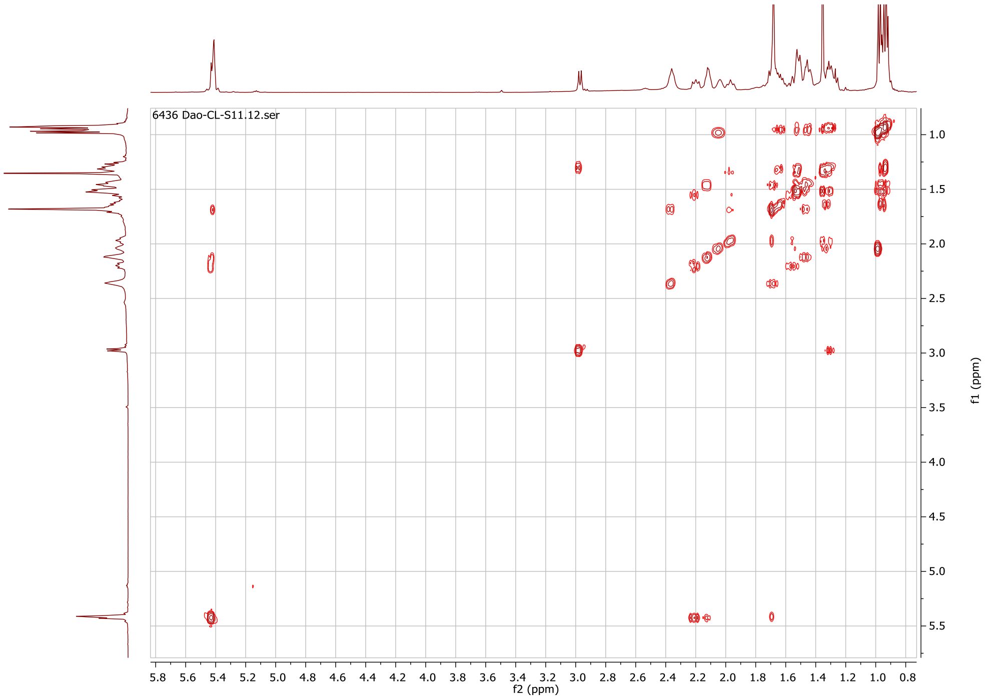

Figure S39: COSY spectrum of **12**.

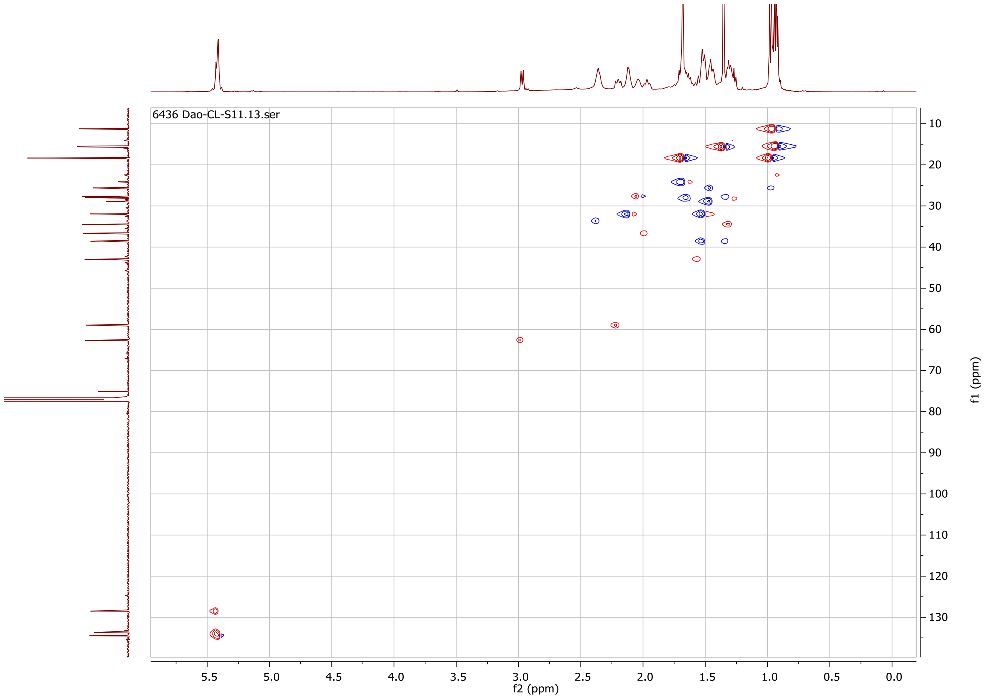

Figure S40: HSQC spectrum of **12**.

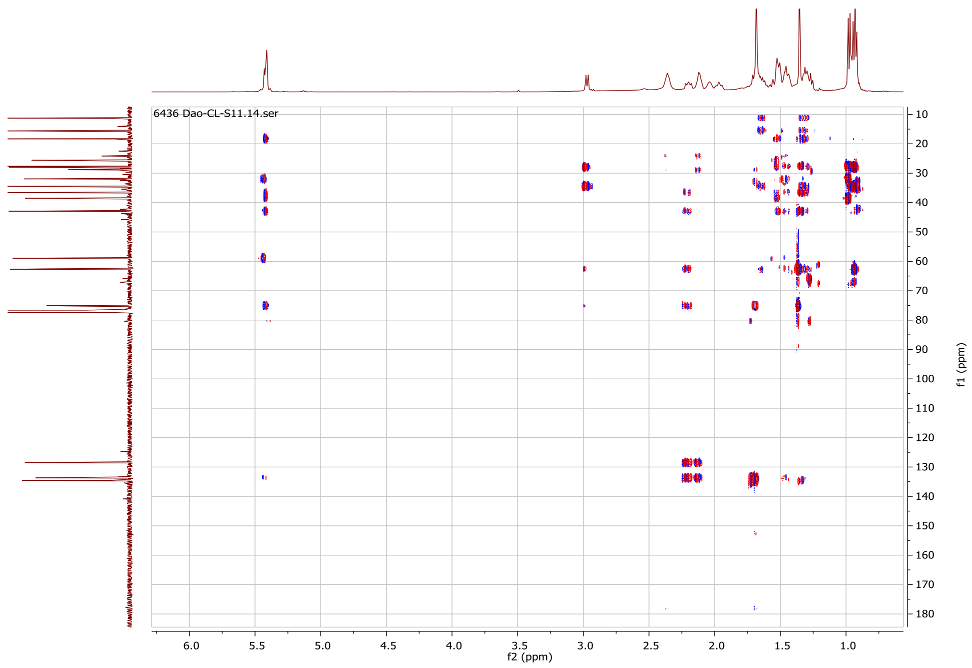

Figure S41: HMBC spectrum of **12**.

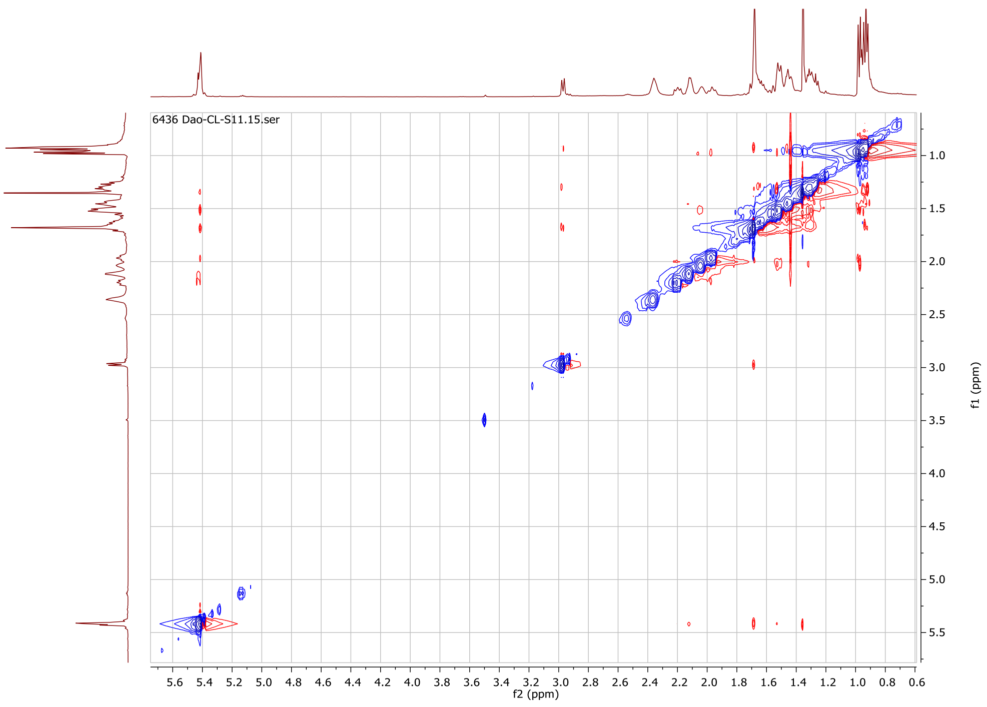

Figure S42: NOESY spectrum **12**.

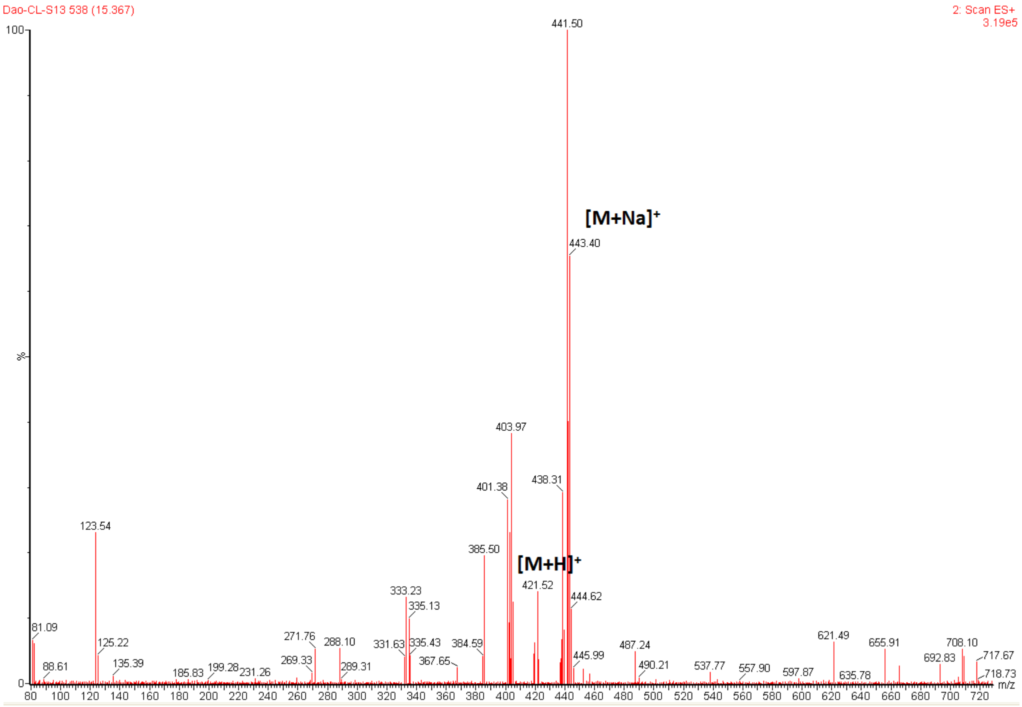

Figure S43: (+)ESI-MS spectrum of **13**.

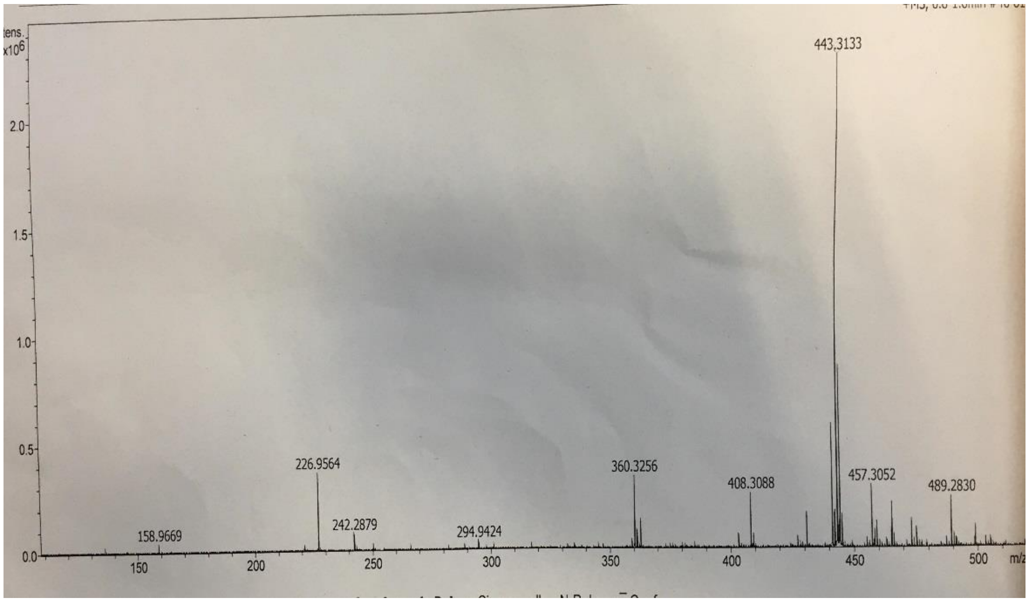

Figure S44: HRESIMS spectrum of **13**.

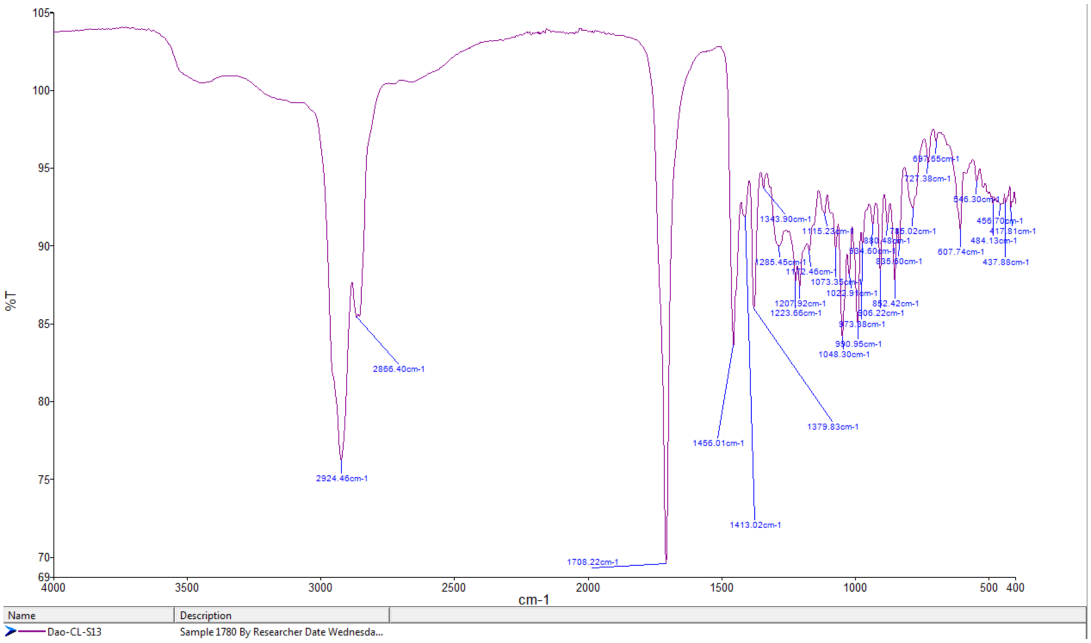

Figure S45: IR spectrum of **13** (film, KBr disc).

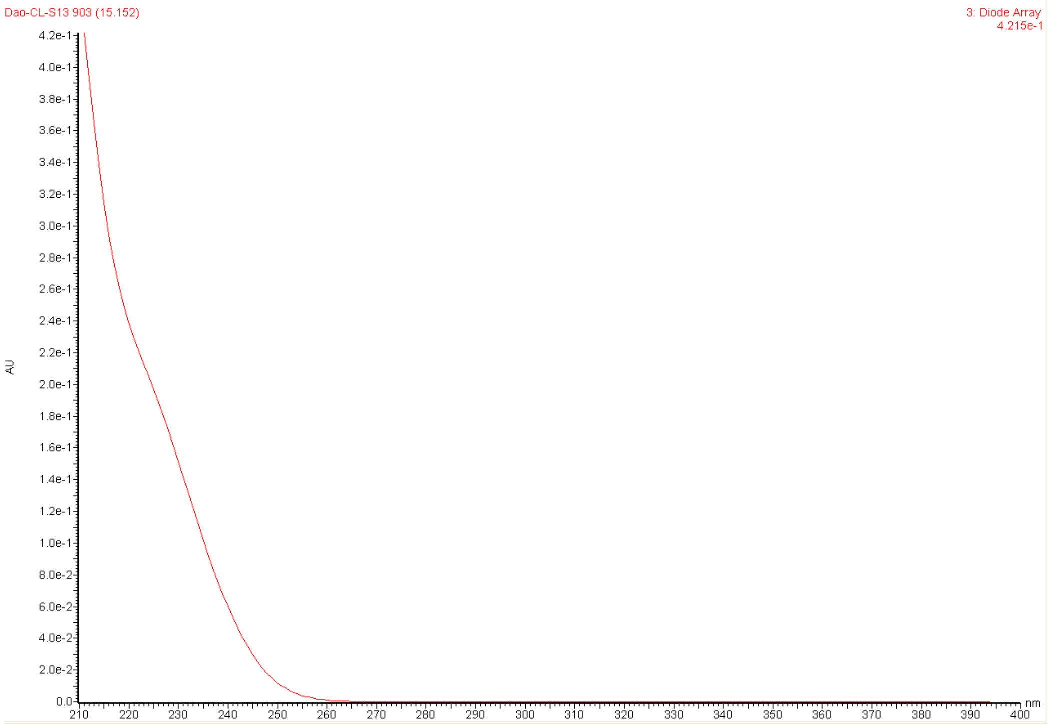

Figure S46: UV/vis (DAD) spectrum of **13** (MeOH)

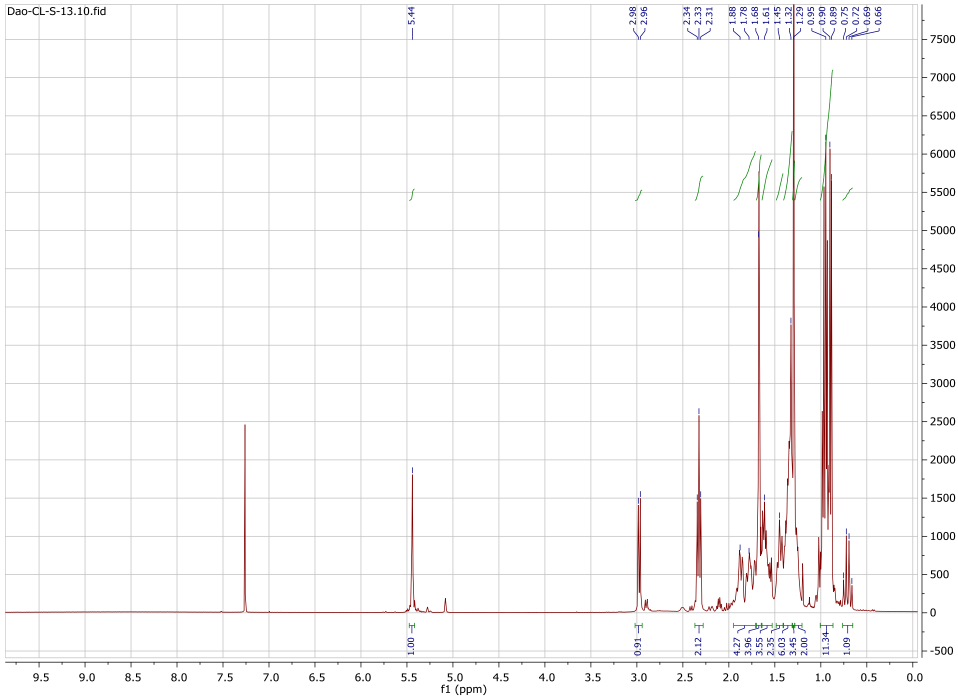

Figure S47: ^{1}H NMR spectrum (500 MHz, $CDCl_3$) of **13**.

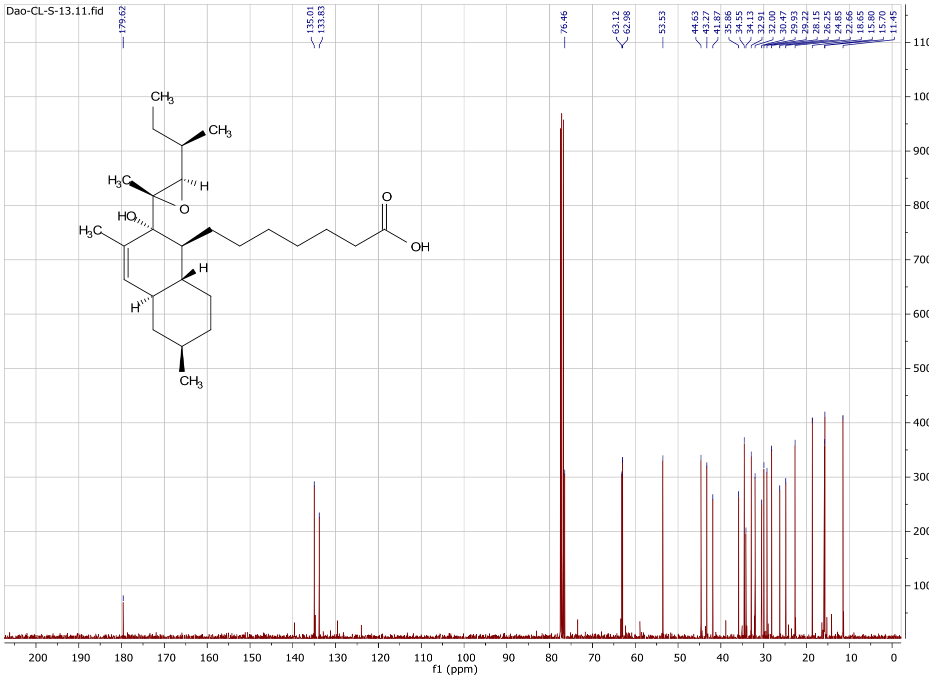

Figure S48: ^{13}C NMR spectrum (125 MHz, $CDCl_3$) of **13**.

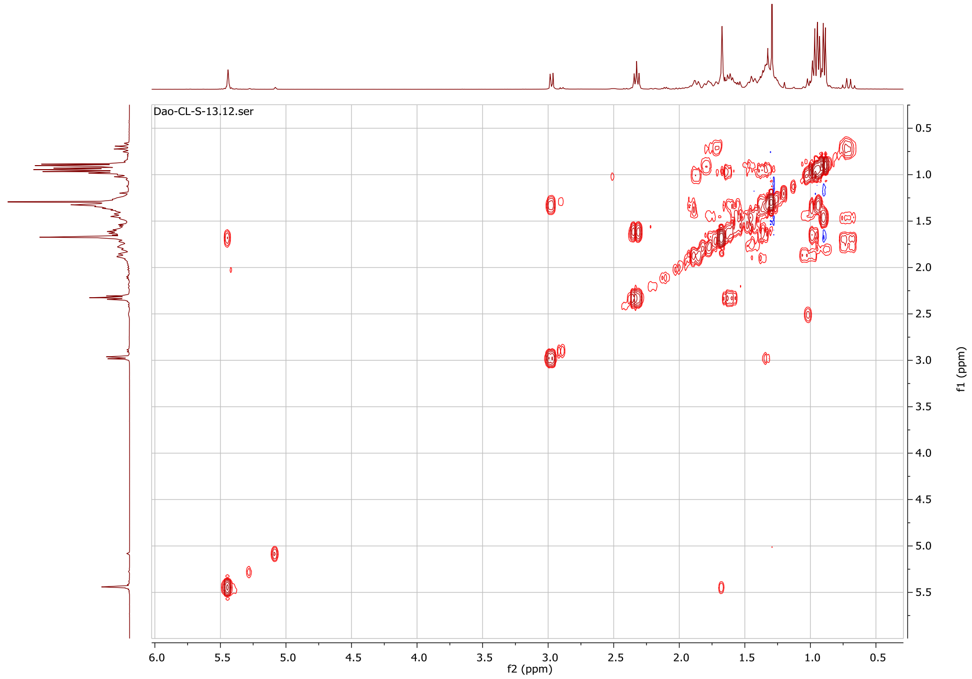

Figure S49: COSY spectrum of **13**.

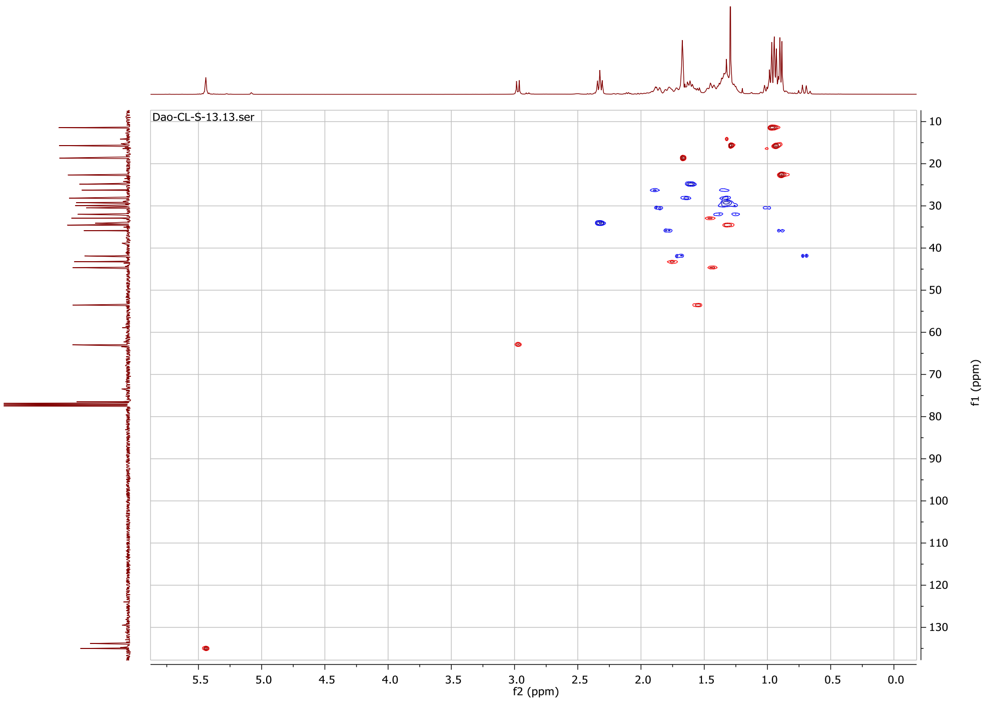

Figure S50: HSQC spectrum of **13**.

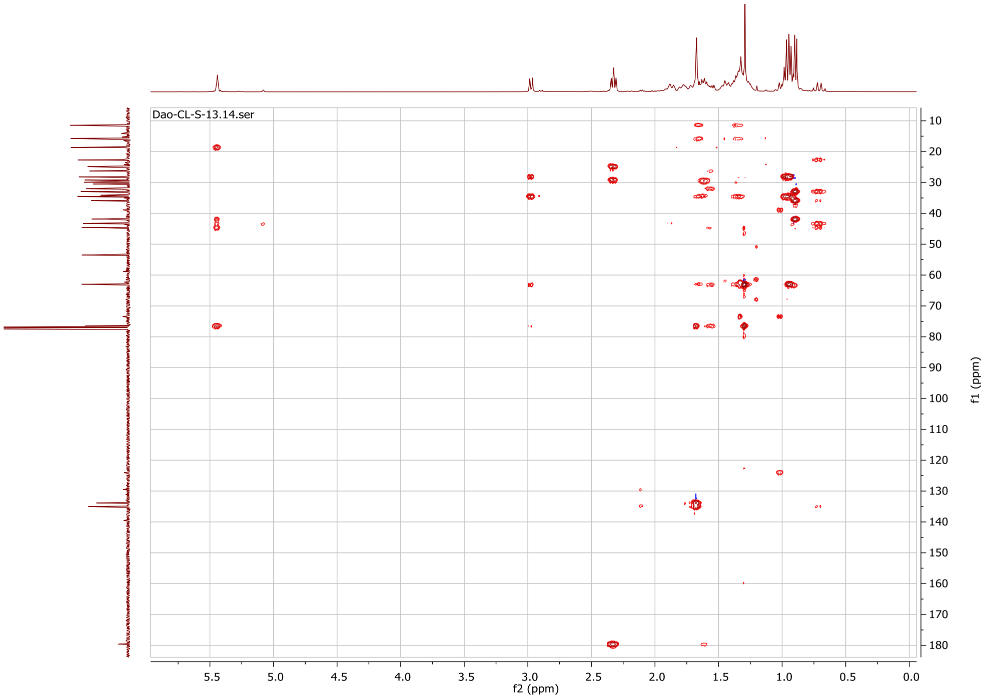

Figure S51: HMBC spectrum of **13**.

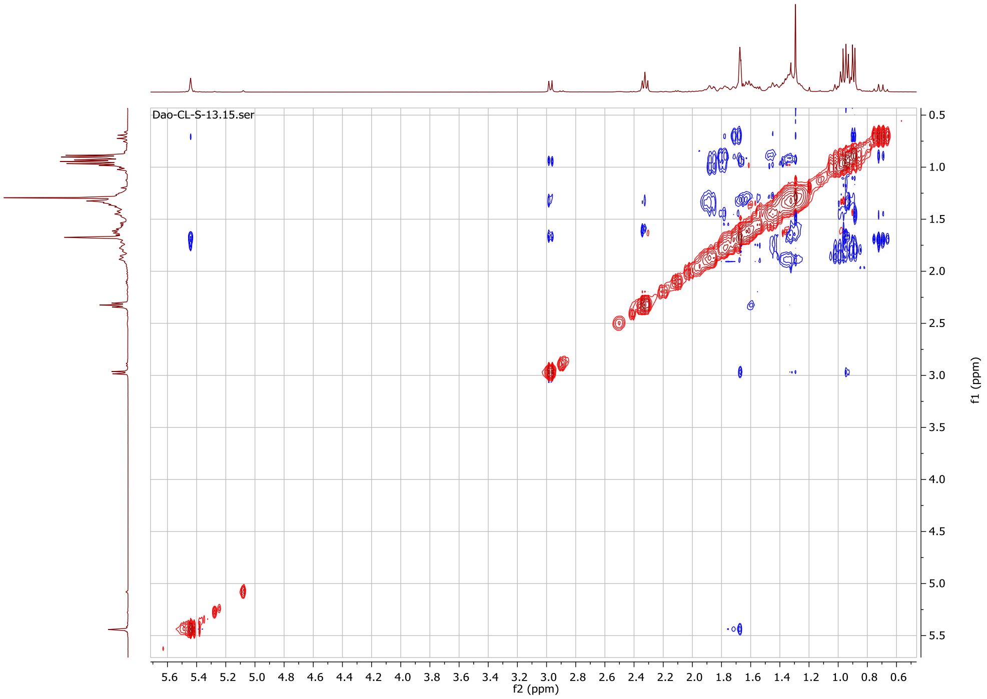

Figure S52: NOESY spectrum **13**.

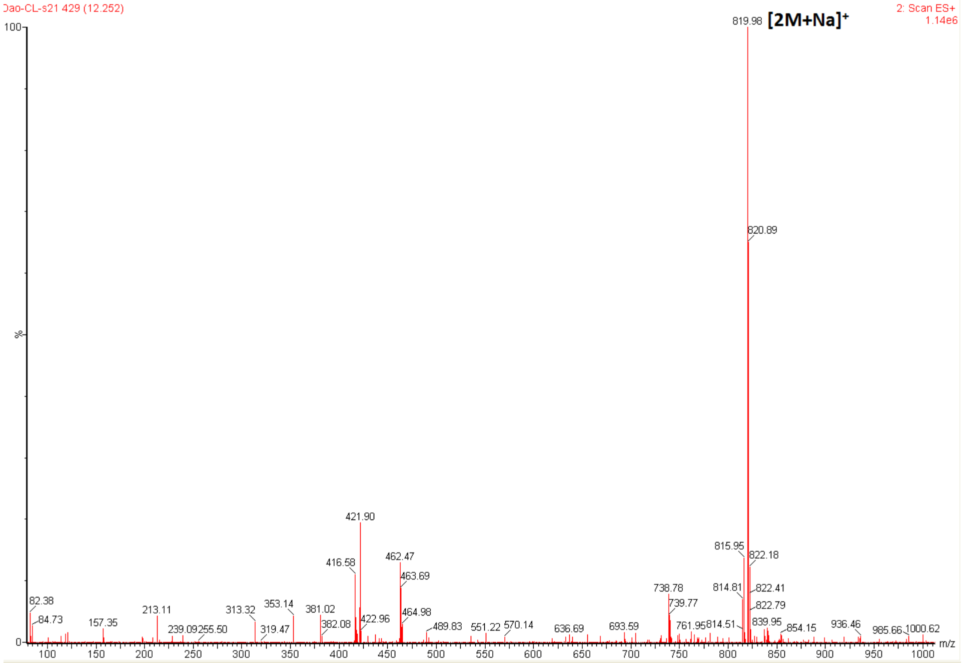

Figure S53: (+)ESI-MS spectrum of **14**.

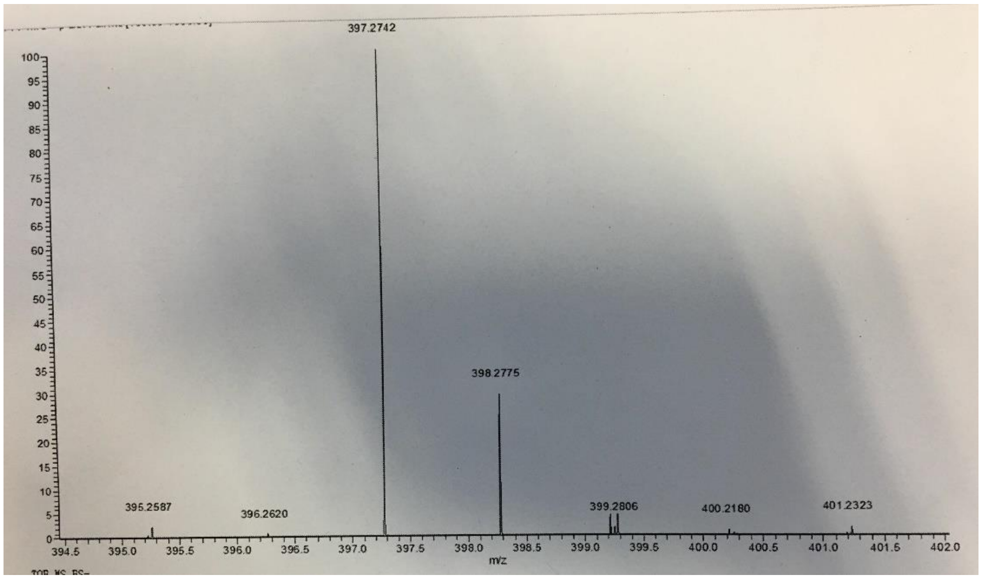

Figure S54: HRESIMS spectrum of **14**.

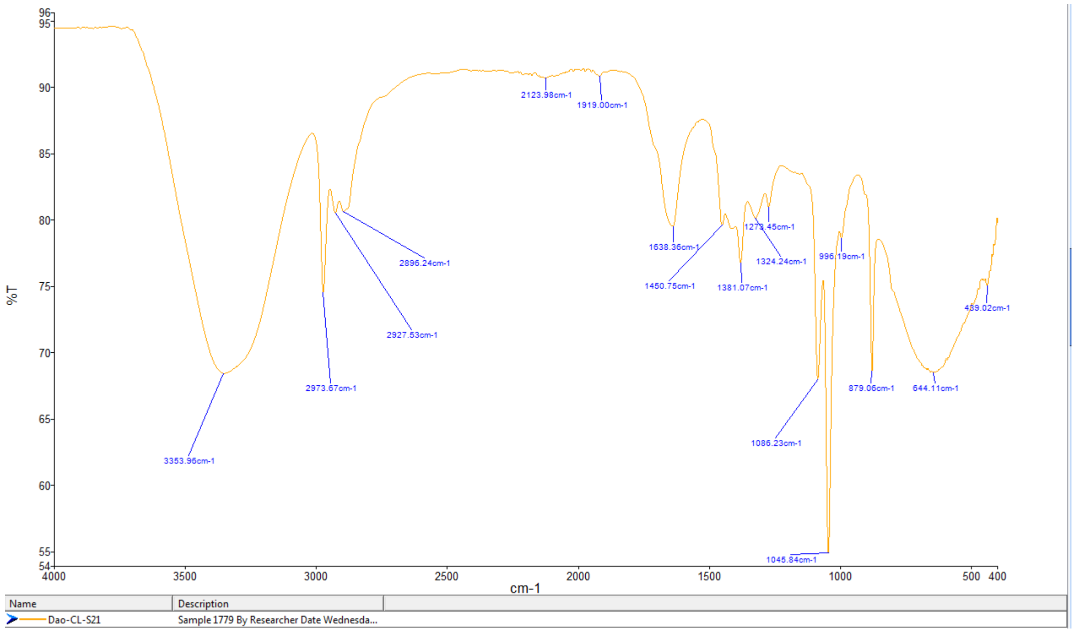

Figure S55: IR spectrum of **14** (film, KBr disc).

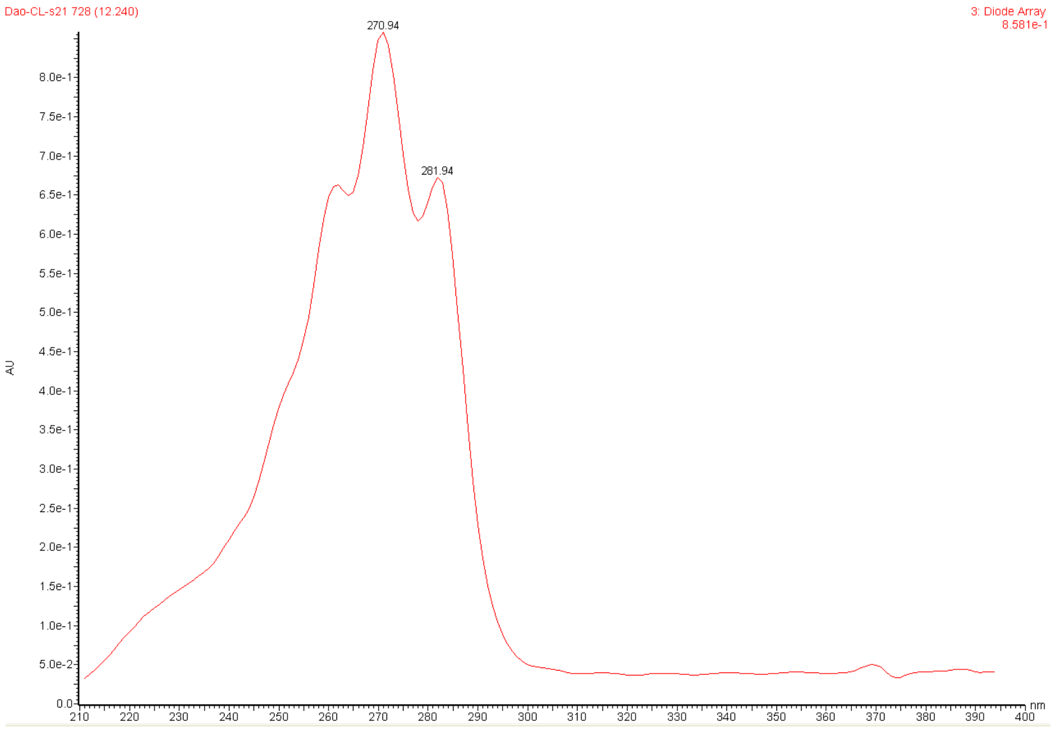

Figure S56: UV/vis (DAD) spectrum of **14** (MeOH).

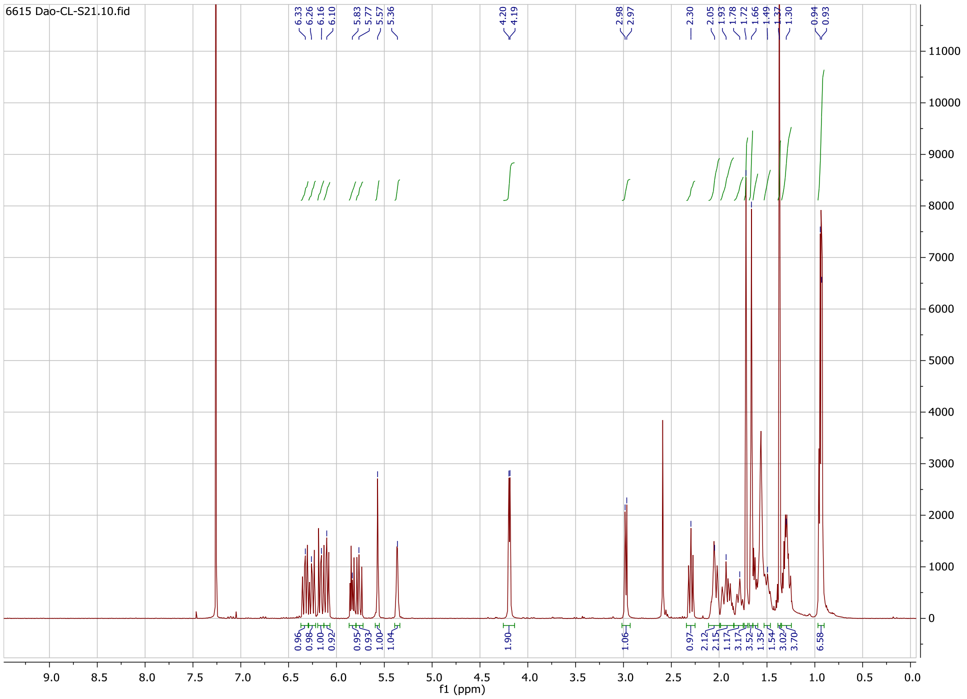

Figure S57: ^{1}H NMR spectrum (500 MHz, $CDCl_3$) of **14**.

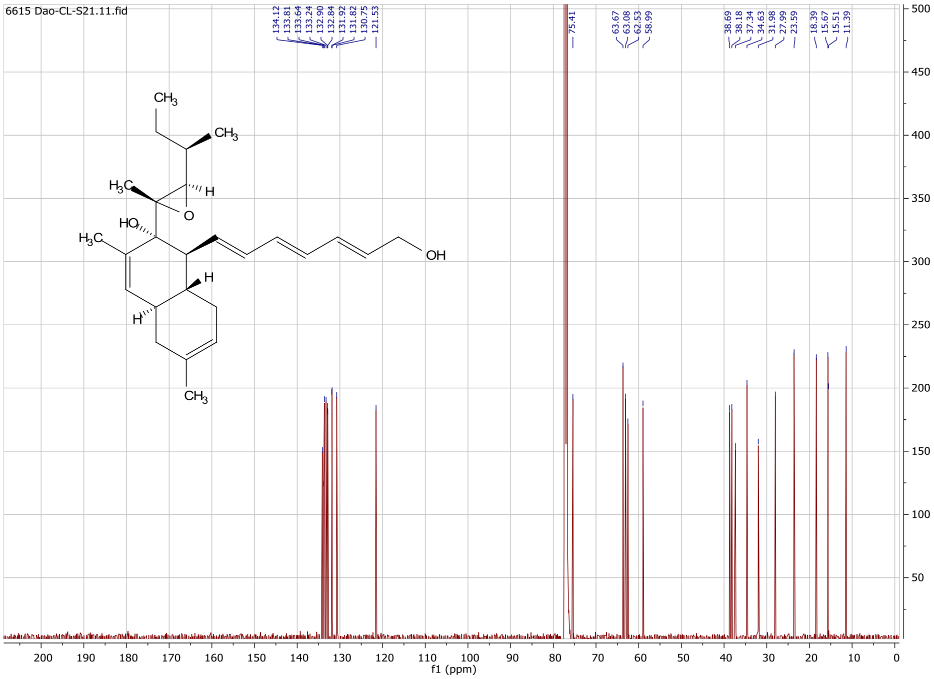

Figure S58: ^{13}C NMR spectrum (125 MHz, $CDCl_3$) of **14**.

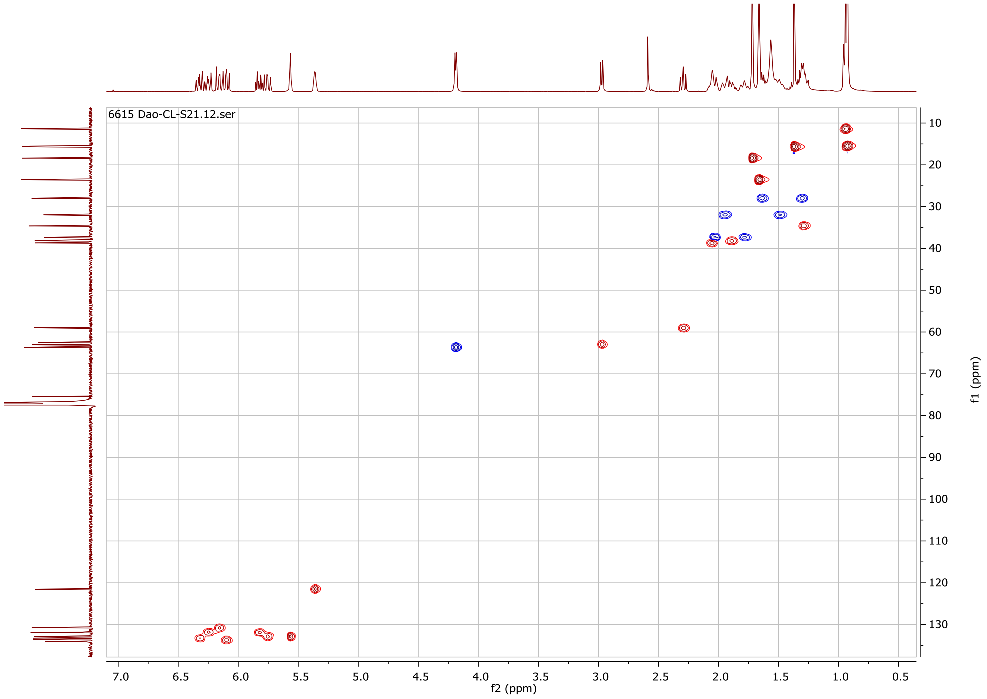

Figure S59: HSQC spectrum of **14**.

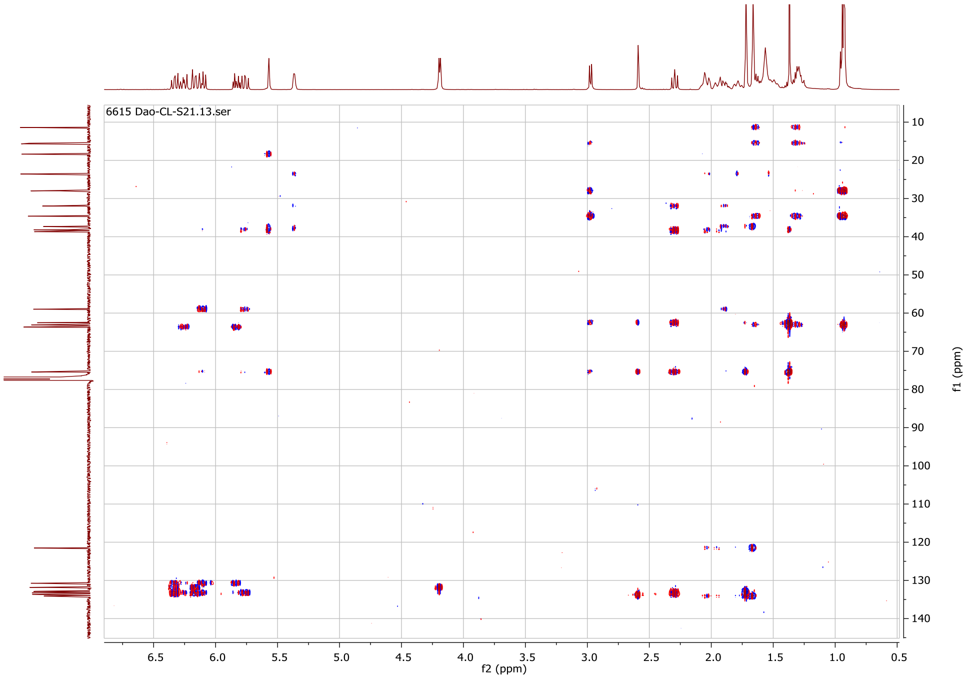

Figure S60: HMBC spectrum of **14**.

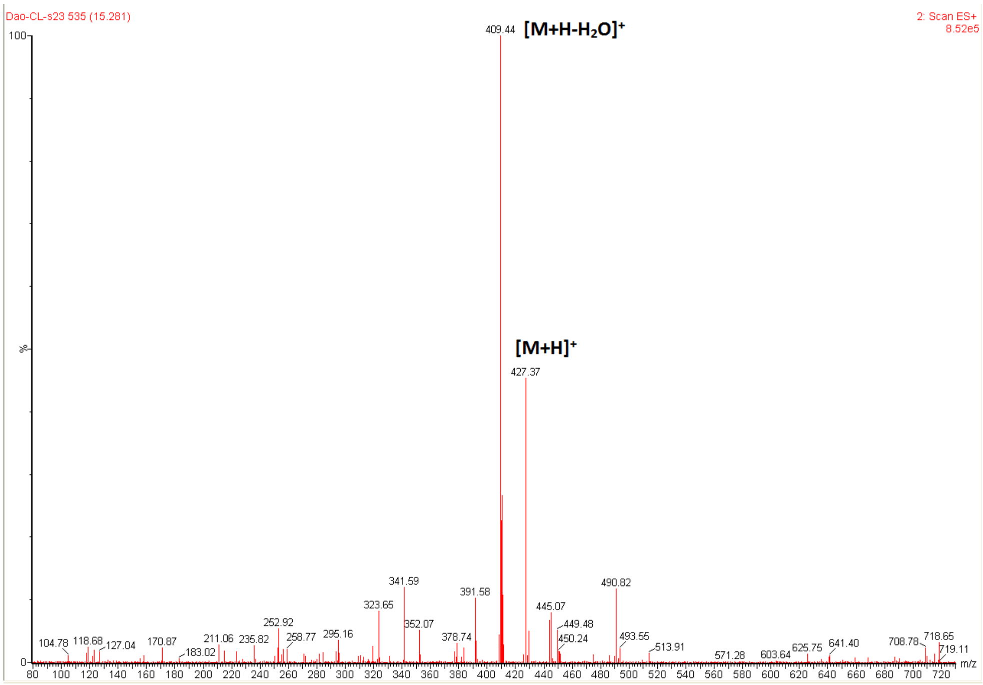

Figure S61: (+)ESI-MS spectrum of **15**.

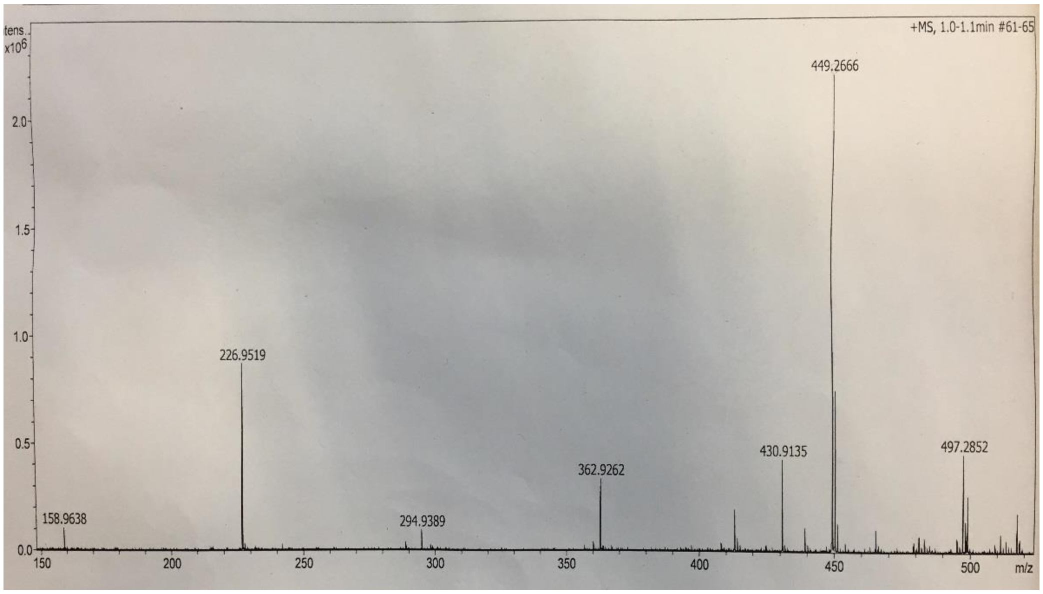

Figure S62: HRESIMS spectrum of **15**.

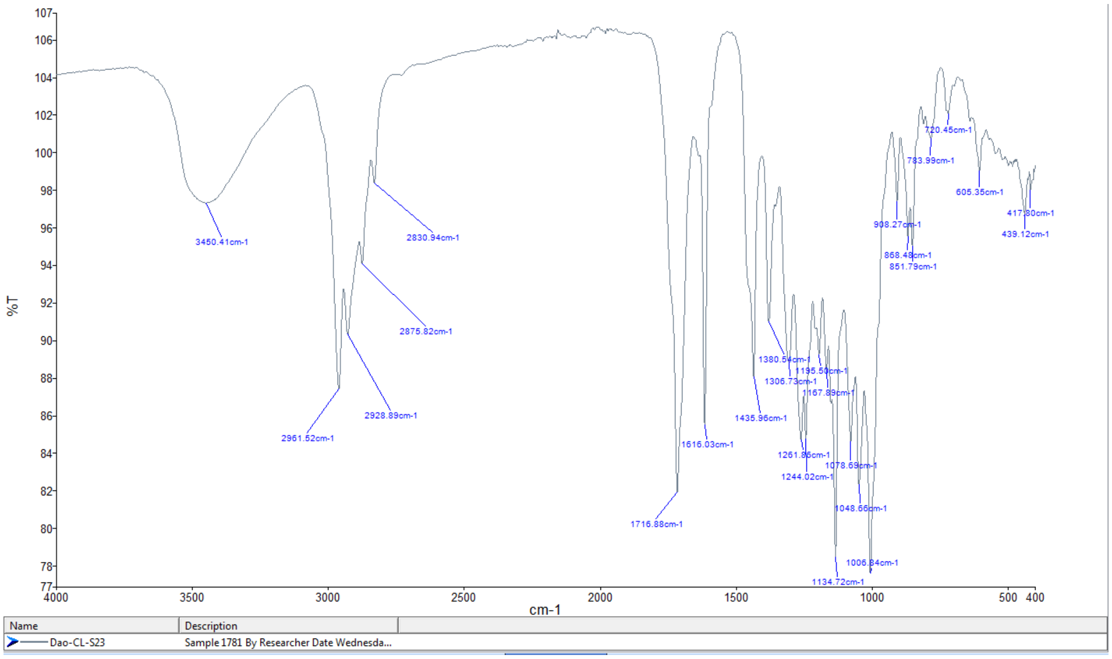

Figure S63: IR spectrum of **15** (film, KBr disc)

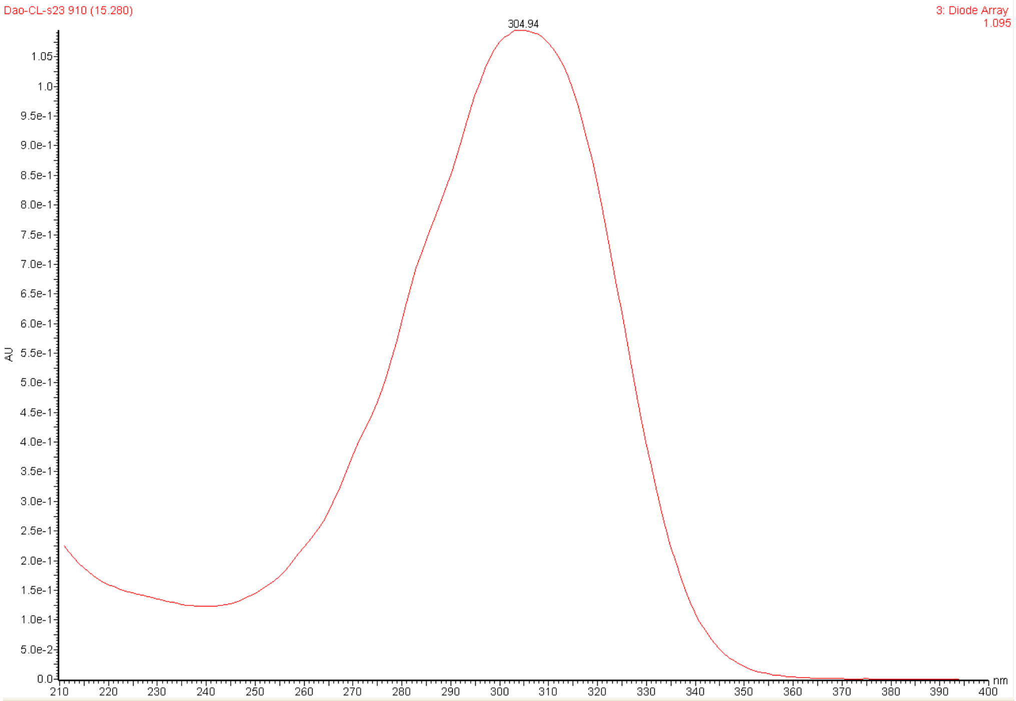

Figure S64: UV/vis (DAD) spectrum of **15** (MeOH).

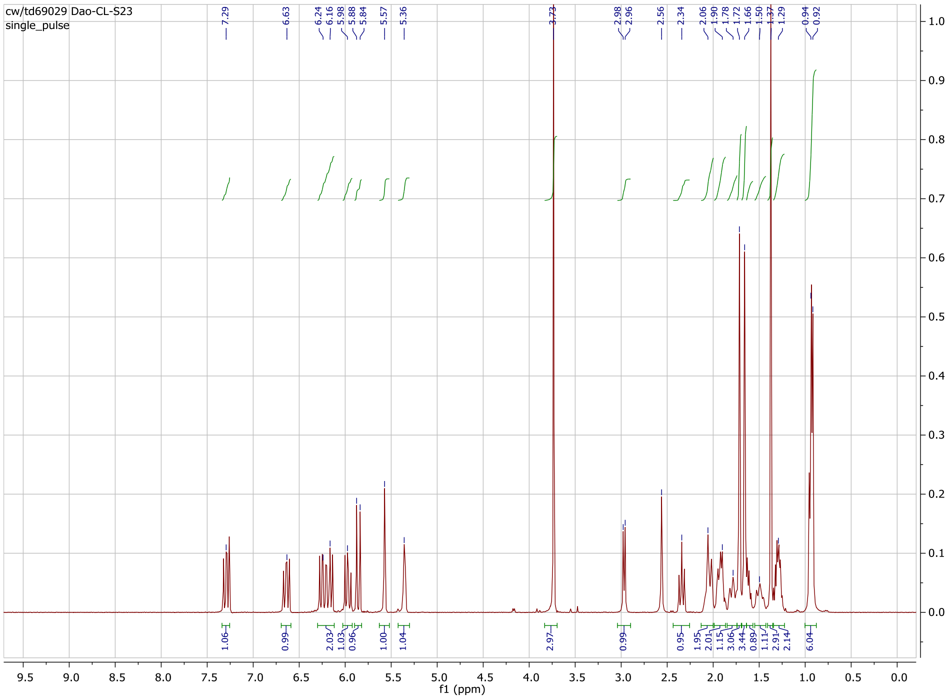

Figure S65: ^{1}H NMR spectrum (500 MHz, $CDCl_3$) of **15**.

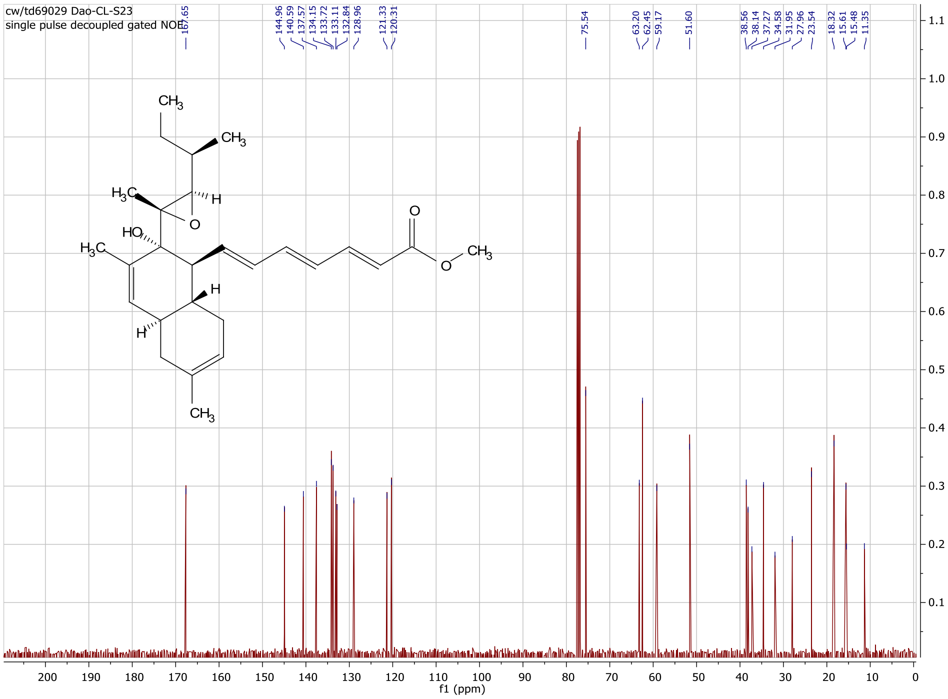

Figure S66: ^{13}C NMR spectrum (125 MHz, $CDCl_3$) of **15**.

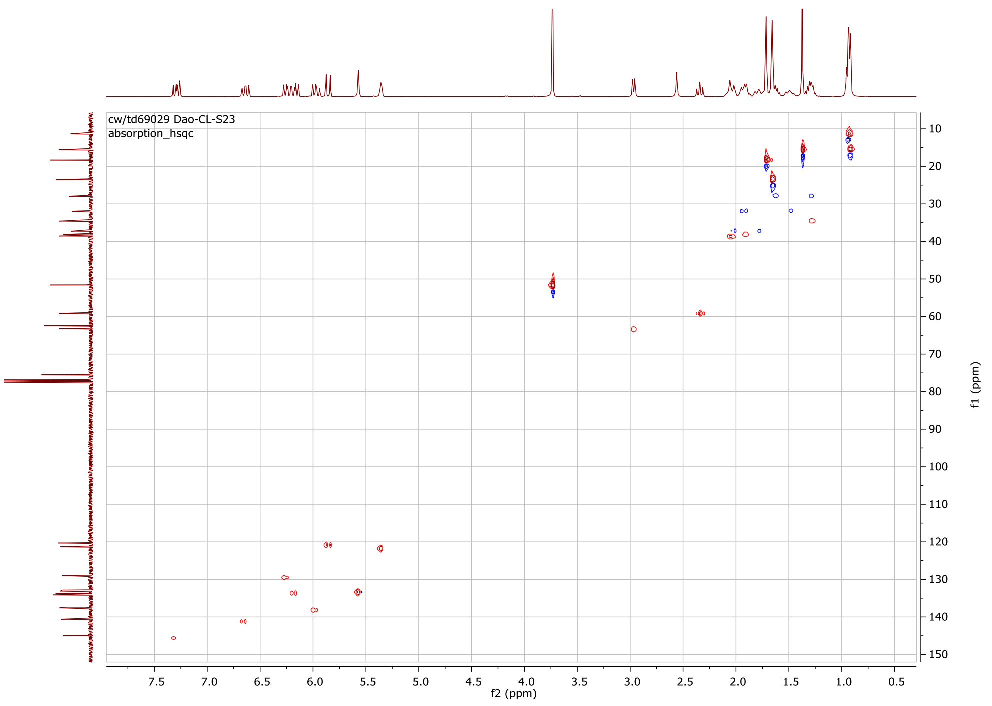

Figure S67: HSQC spectrum of **15**.

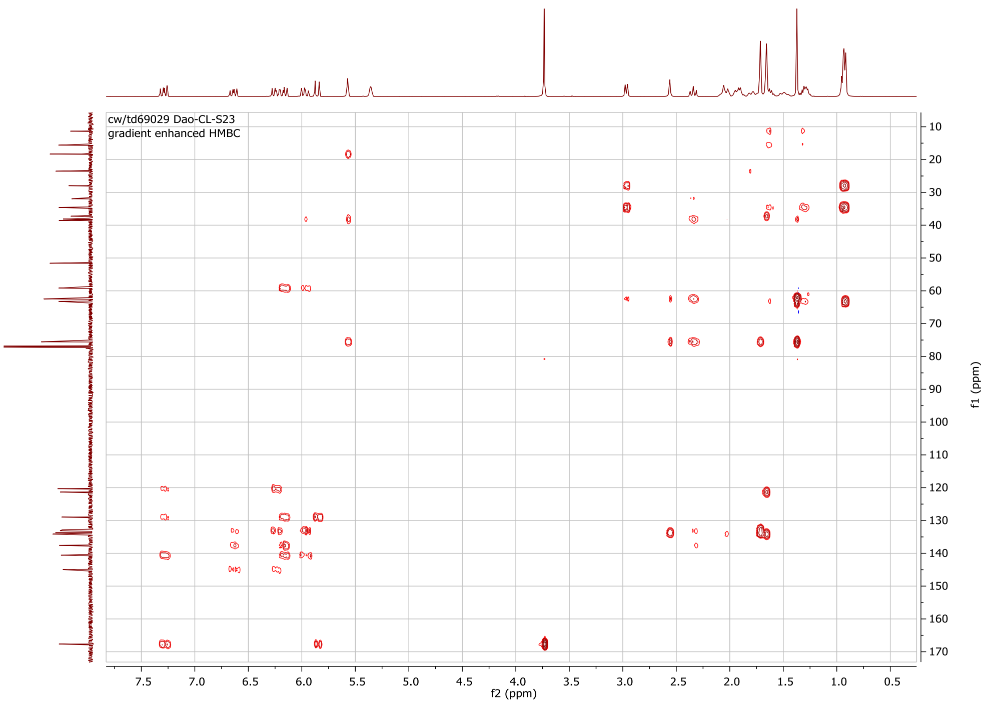

Figure S68: HMBC spectrum of **15**.

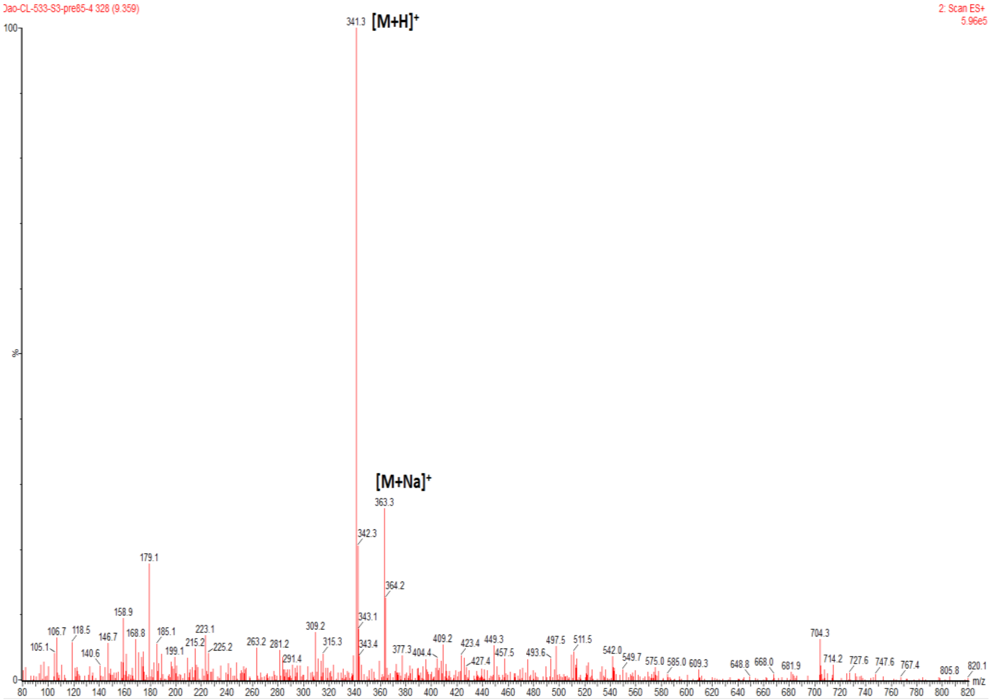

Figure S69: (+)ESI-MS spectrum of **16**.

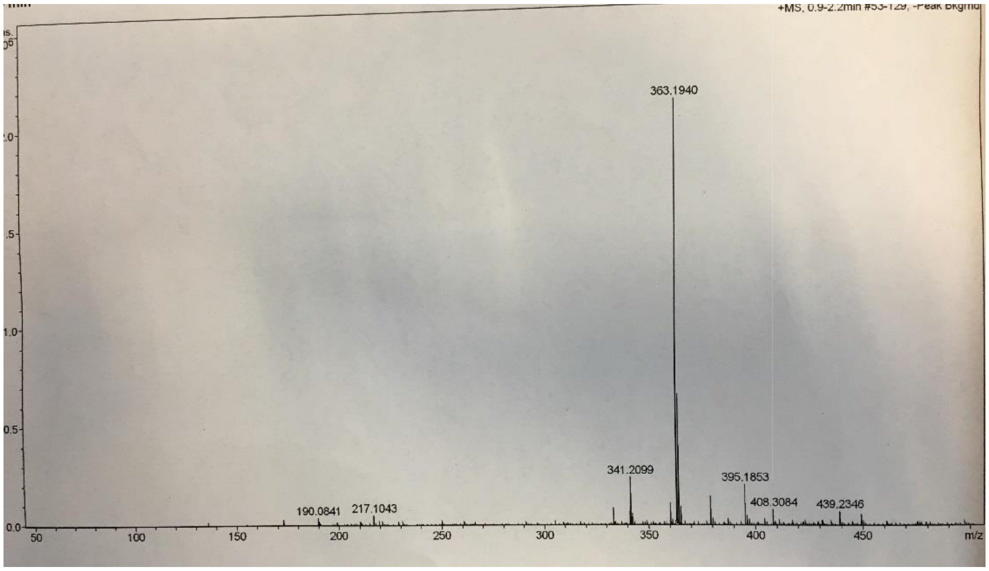

Figure S70: HRESIMS spectrum of **16**.

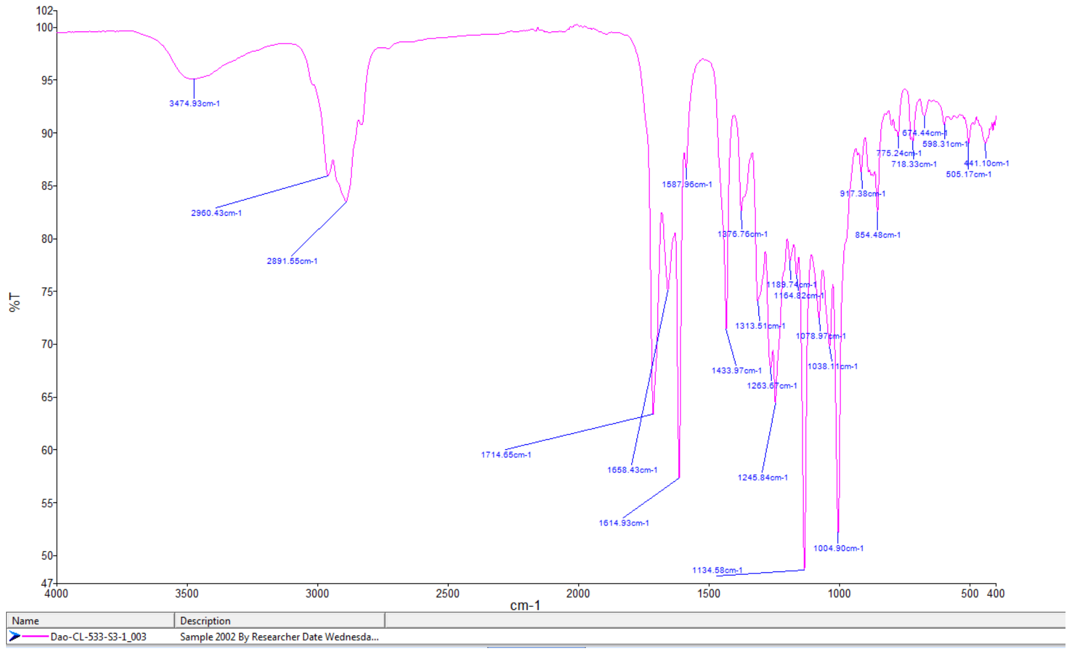

Figure S71: IR spectrum of **16** (film, KBr disc)

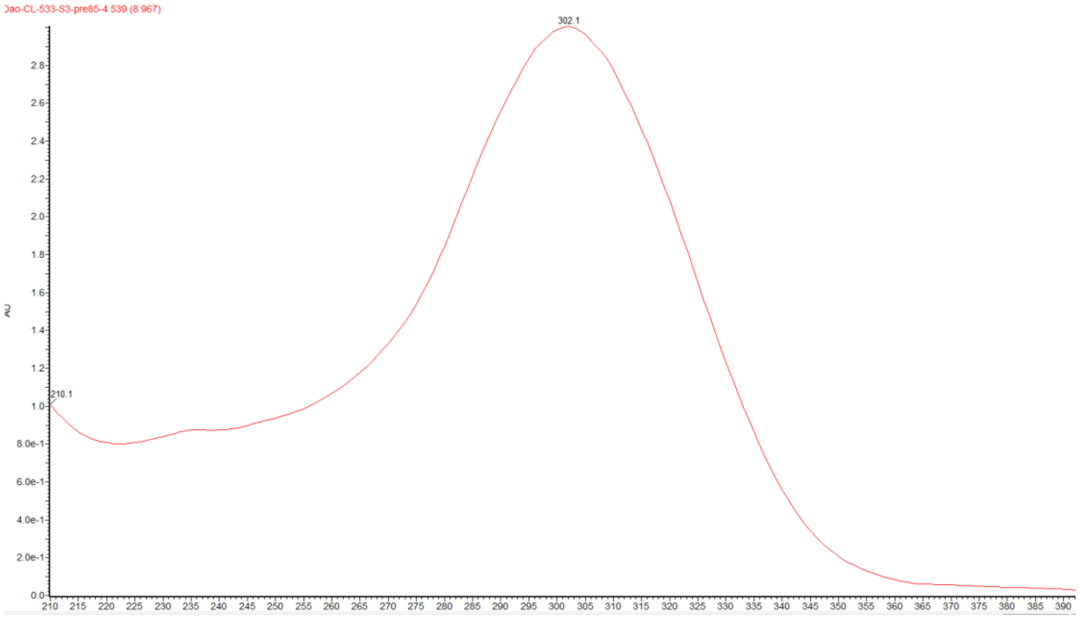

Figure S72: UV/vis (DAD) spectrum of **16** (MeOH)

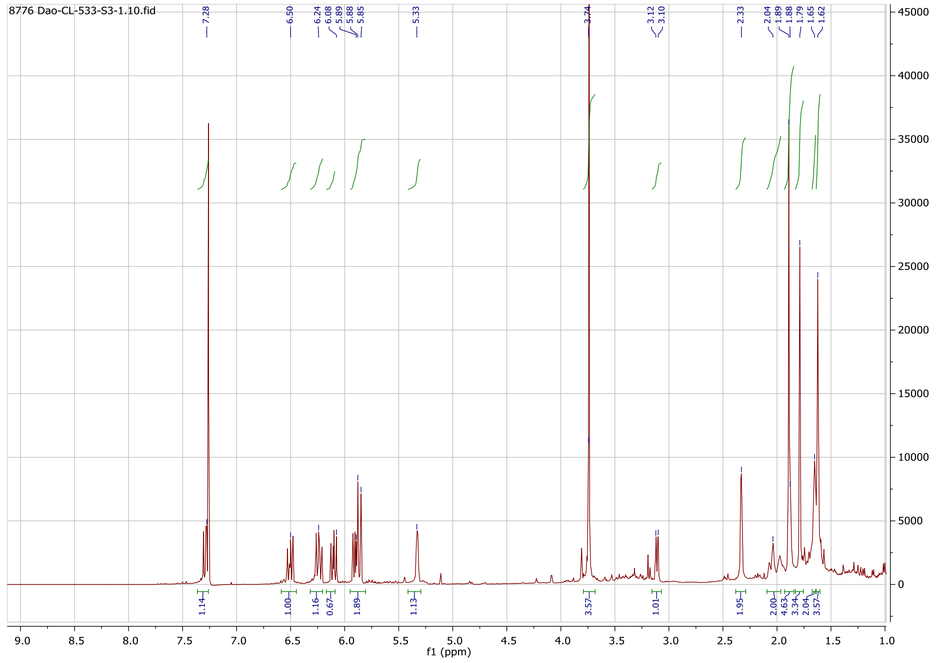

Figure S73: ^{1}H NMR spectrum (500 MHz, $CDCl_3$) of **16**.

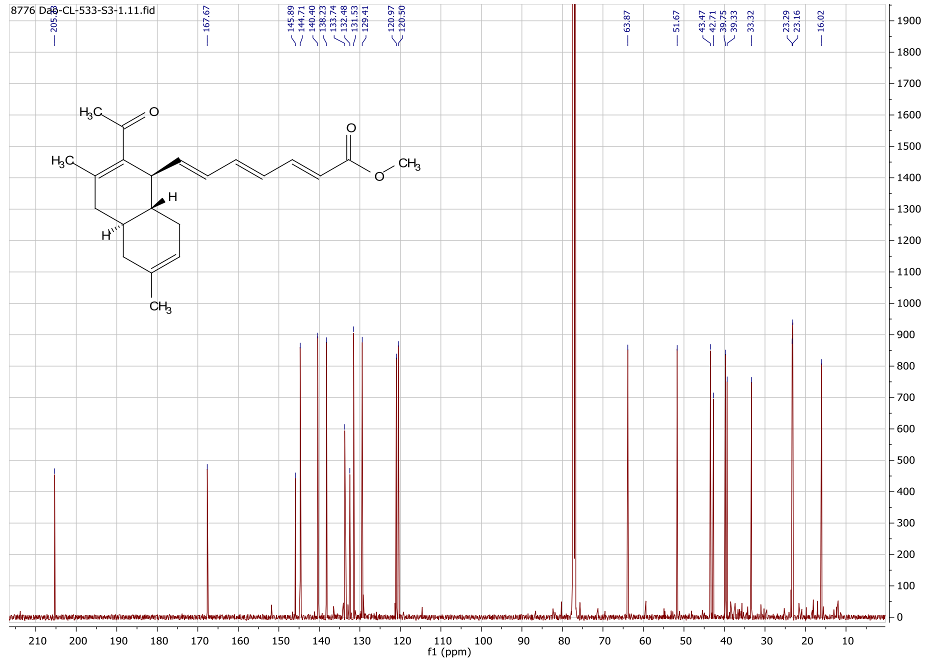

Figure S74: ^{13}C NMR spectrum (125 MHz, $CDCl_3$) of **16**.

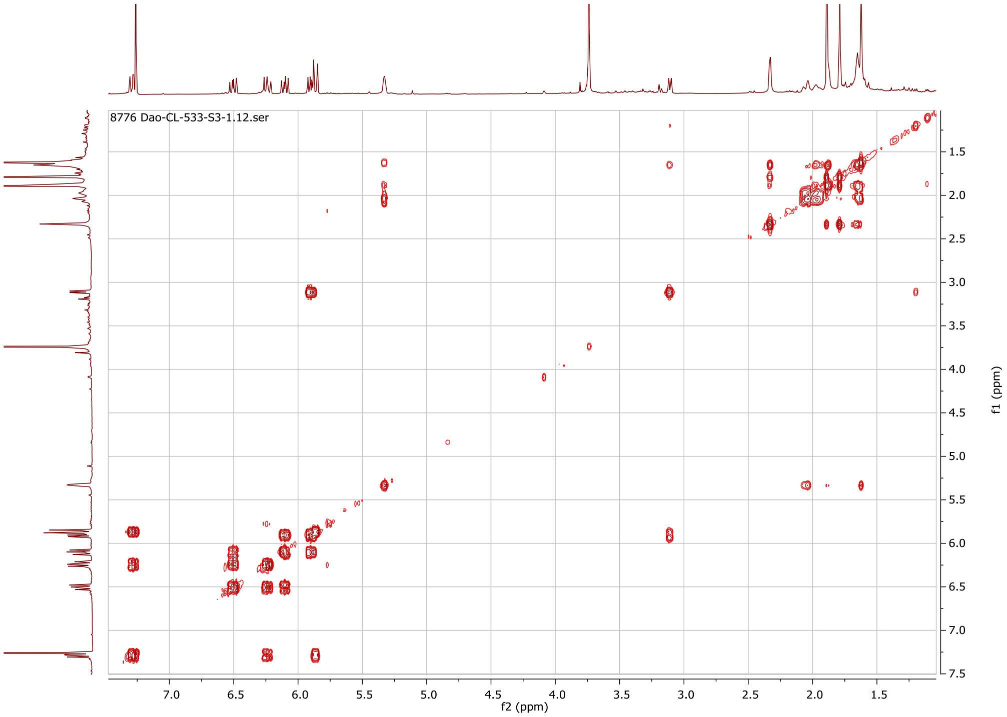

Figure S75: COSY spectrum of **16**.

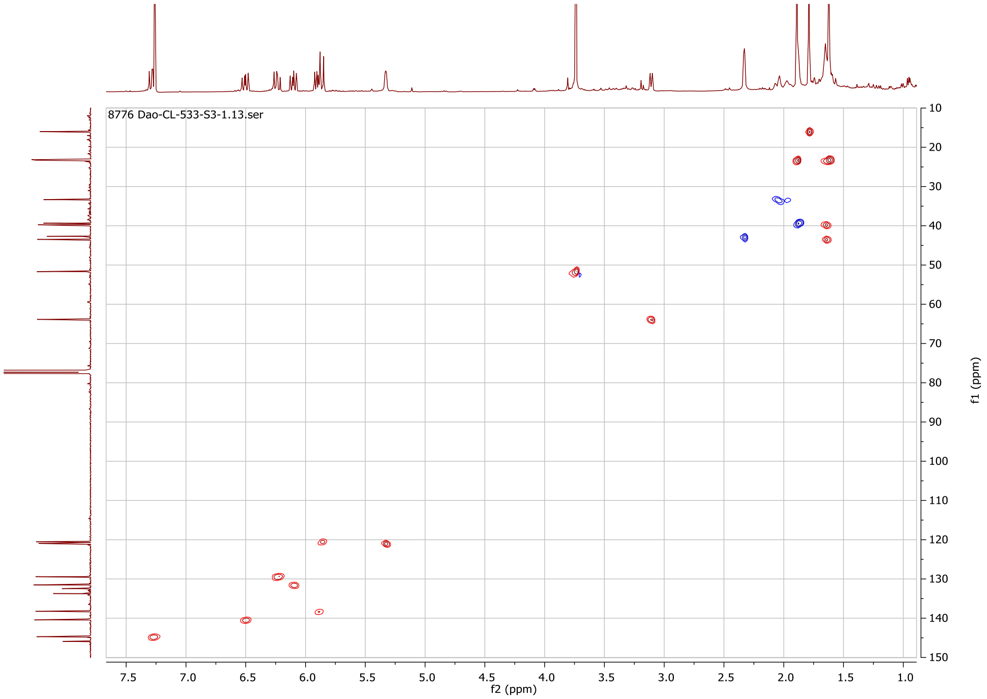

Figure S76: HSQC spectrum of **16**.

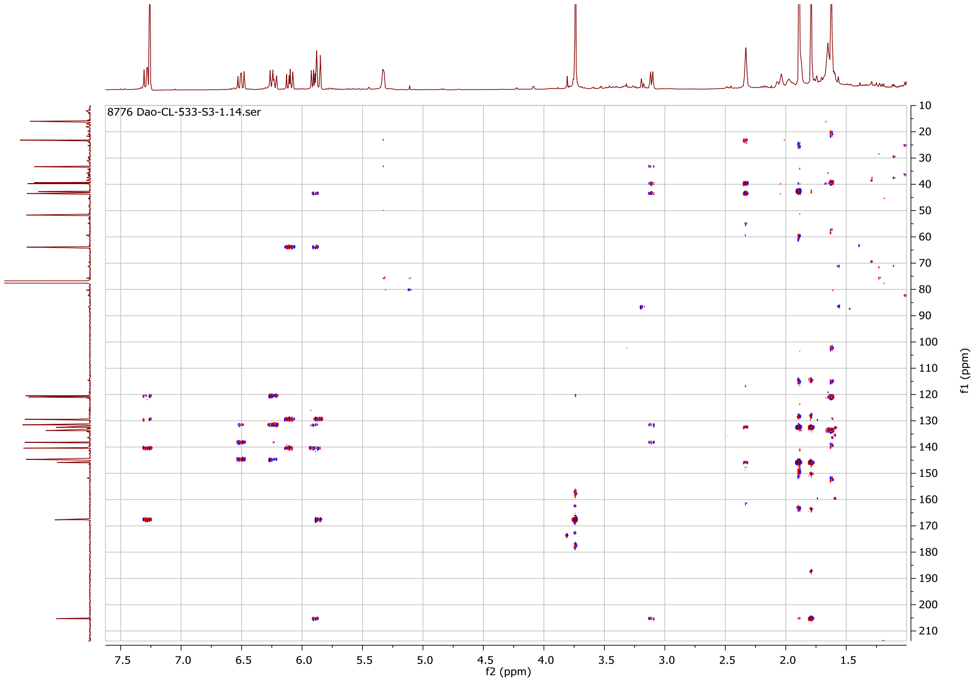

Figure S77: HMBC spectrum of **16**.

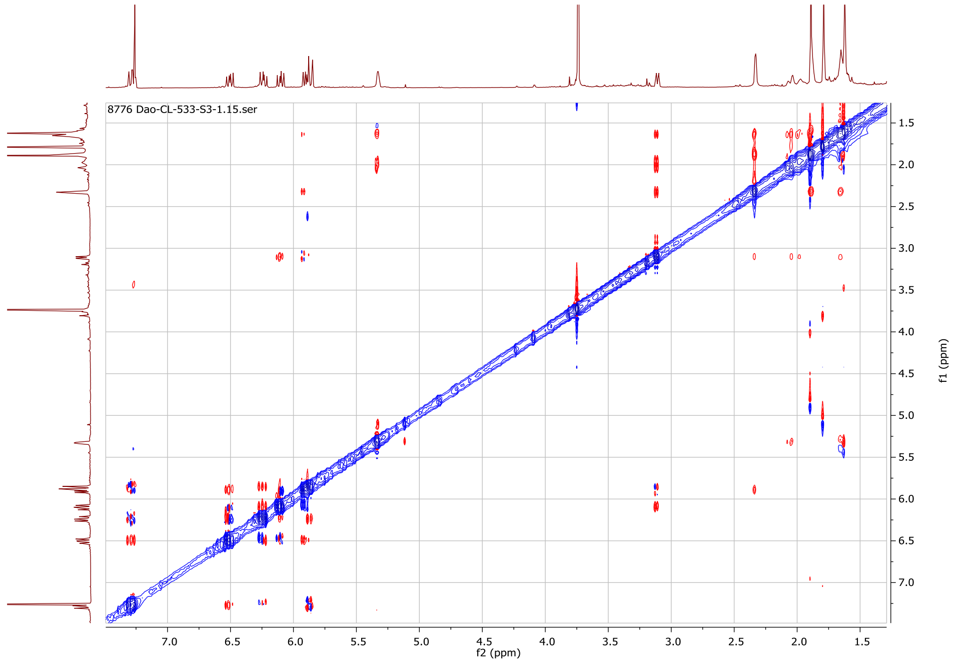

Figure S78: NOESY spectrum **16**.

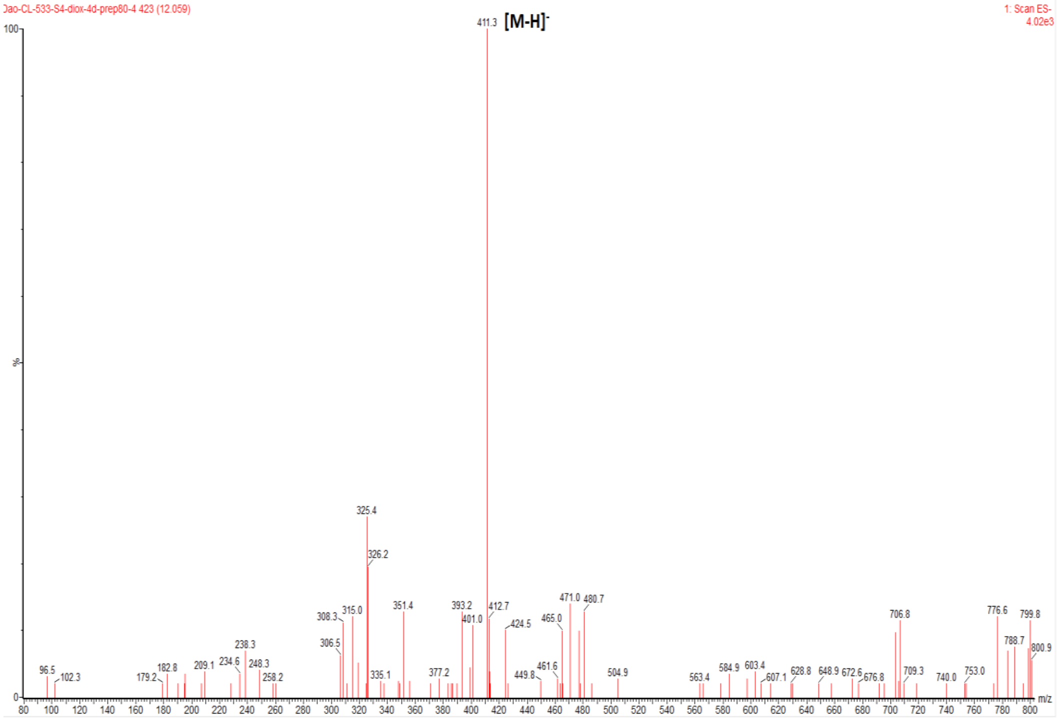

Figure S79: (-)ESI-MS spectrum of **17**.

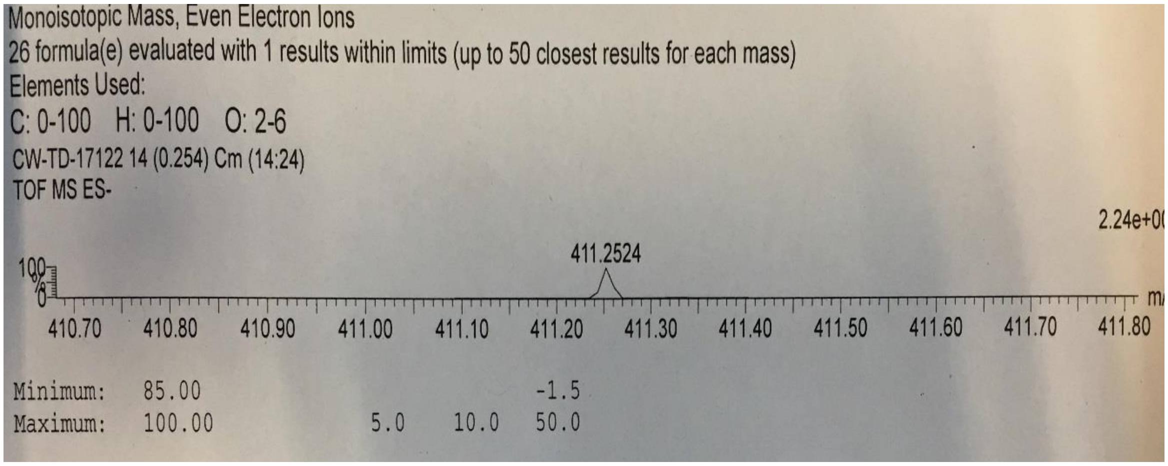
Monoisotopic Mass, Even Electron Ions
26 formula(e) evaluated with 1 results within limits (up to 50 closest results for each mass)
Elements Used:
C: 0-100 H: 0-100 O: 2-6
CW-TD-17122 14 (0.254) Cm (14:24)
TOF MS ES-

Minimum:	85.00			-1.5
Maximum:	100.00	5.0	10.0	50.0

Figure S80: HRESIMS spectrum of **17**.

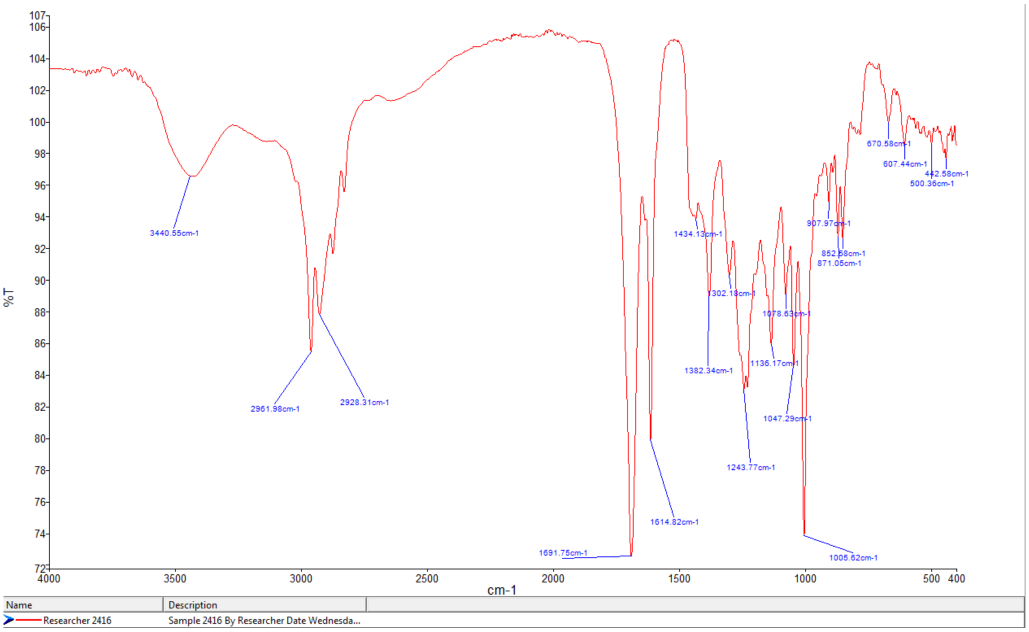

Figure S81: IR spectrum of **17** (film, KBr disc).

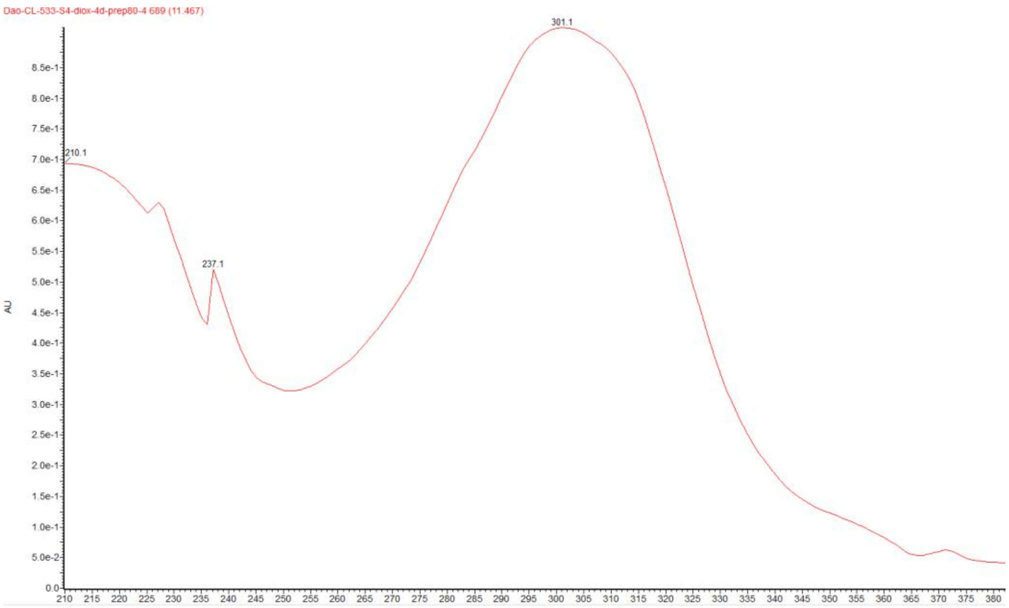

Figure S82: UV/vis (DAD) spectrum of **17** (MeOH)

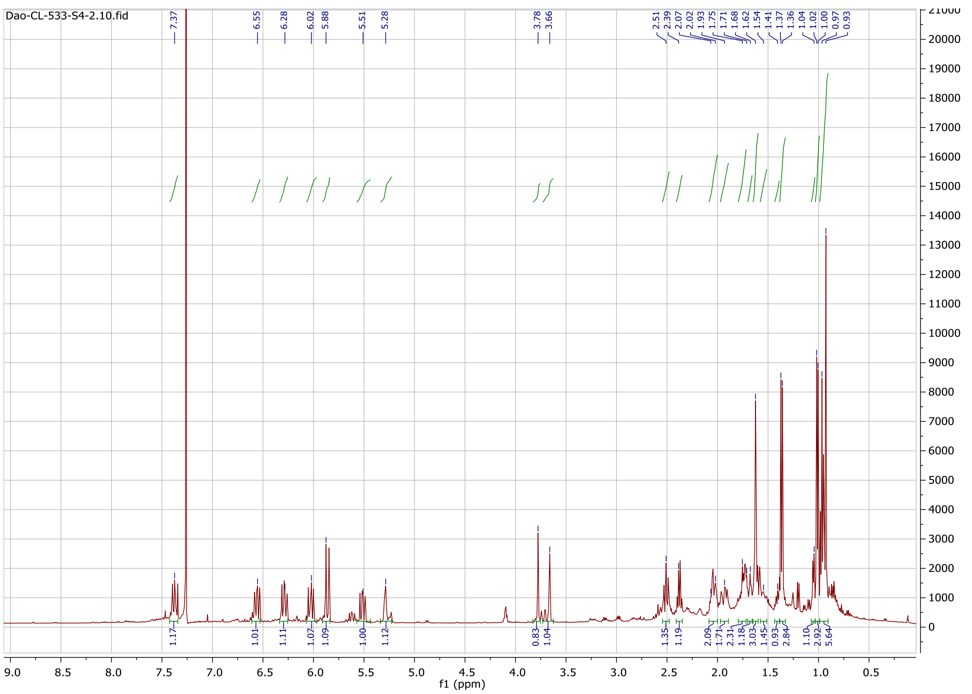

Figure S83: ^{1}H NMR spectrum (500 MHz, $CDCl_3$) of **17**.

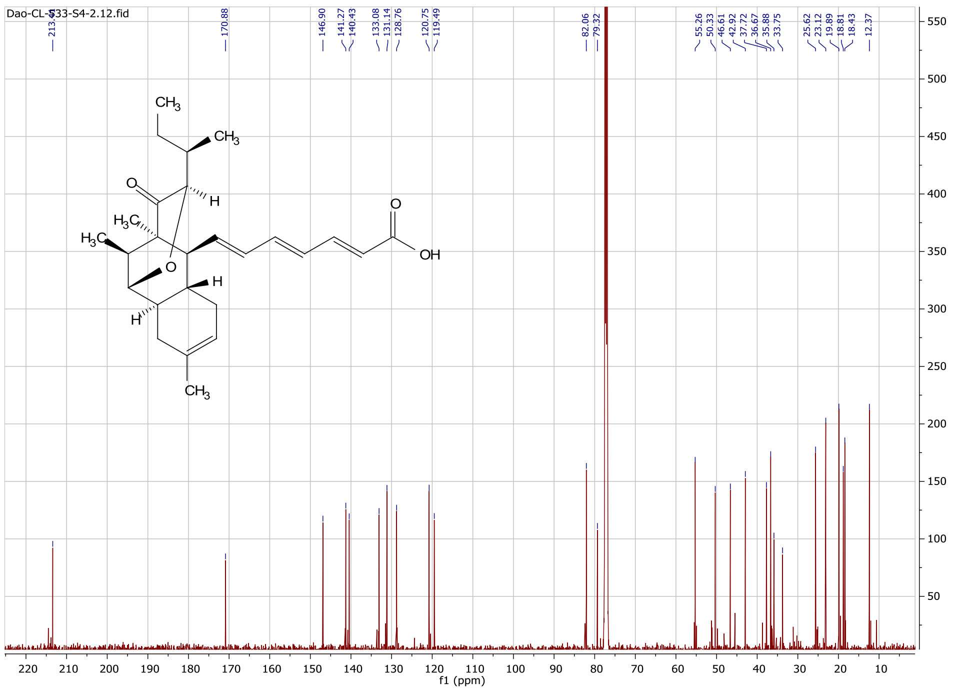

Figure S84: ^{13}C NMR spectrum (125 MHz, $CDCl_3$) of **17**.

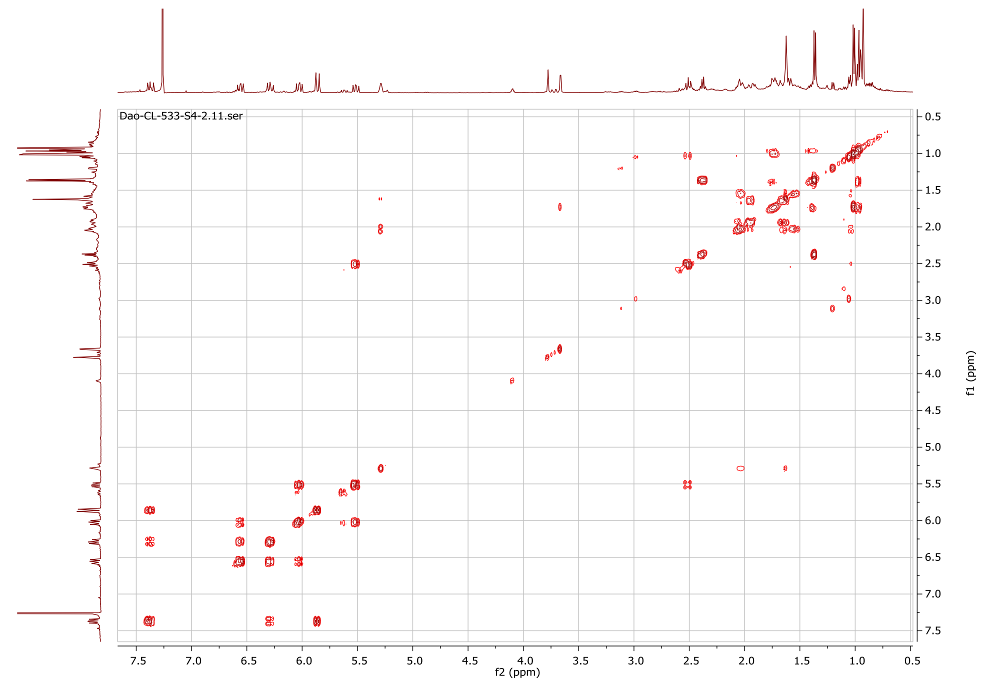

Figure S85: COSY spectrum of **17**.

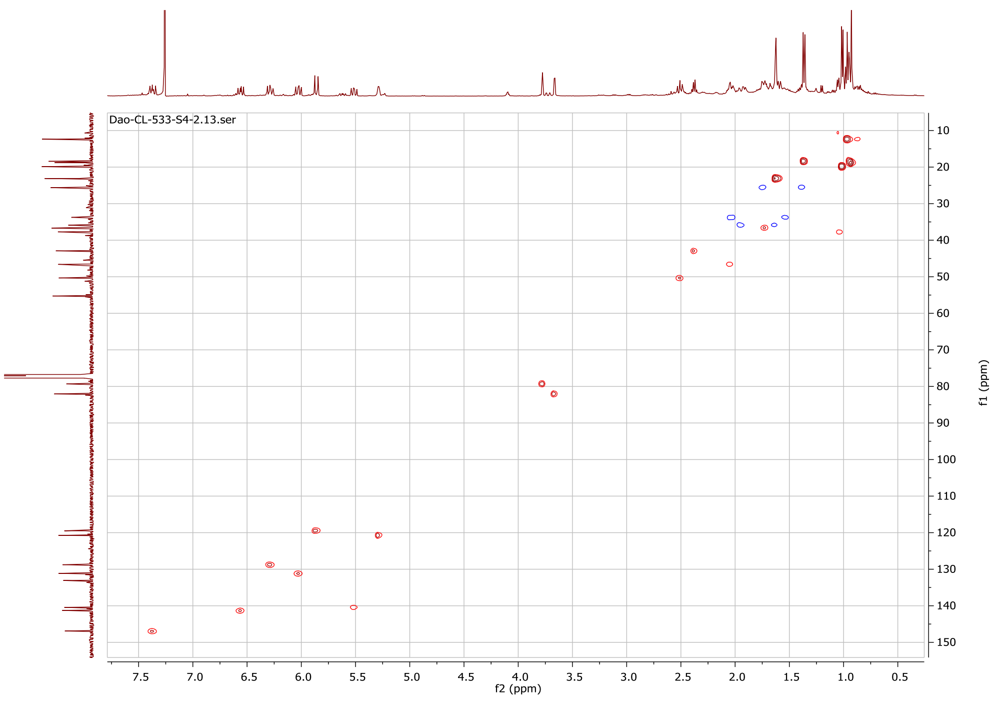

Figure S86: HSQC spectrum of **17**.

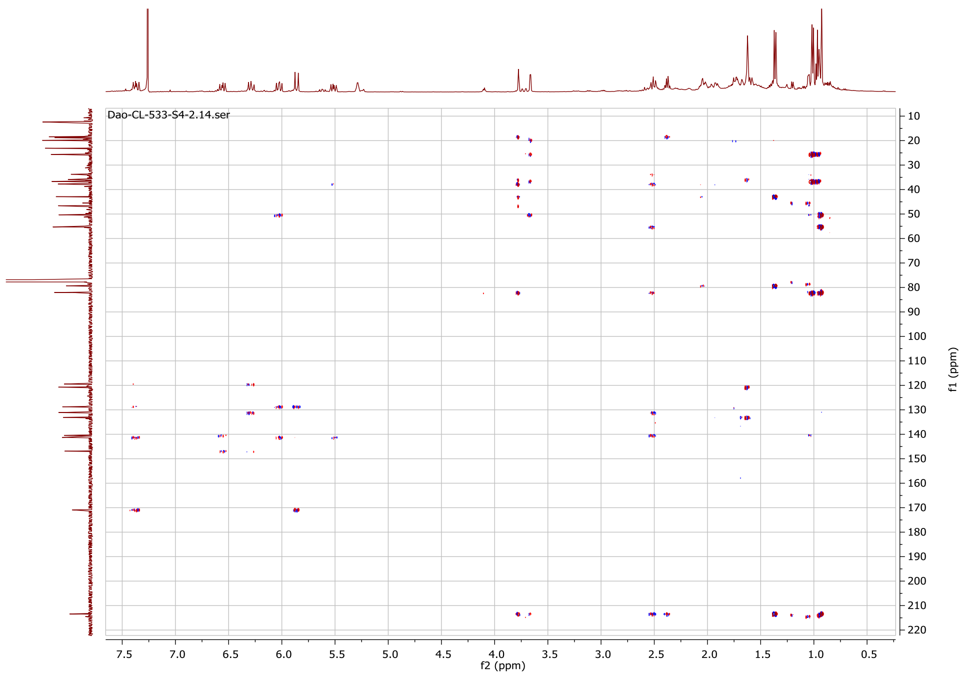

Figure S87: HMBC spectrum of **17**.

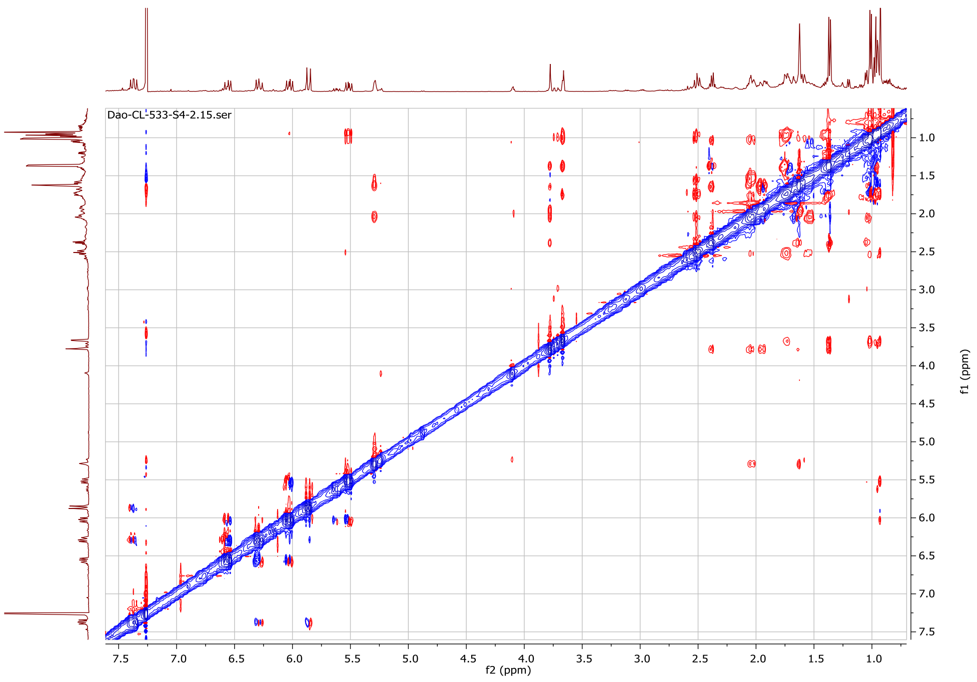

Figure S88: NOESY spectrum **17**.

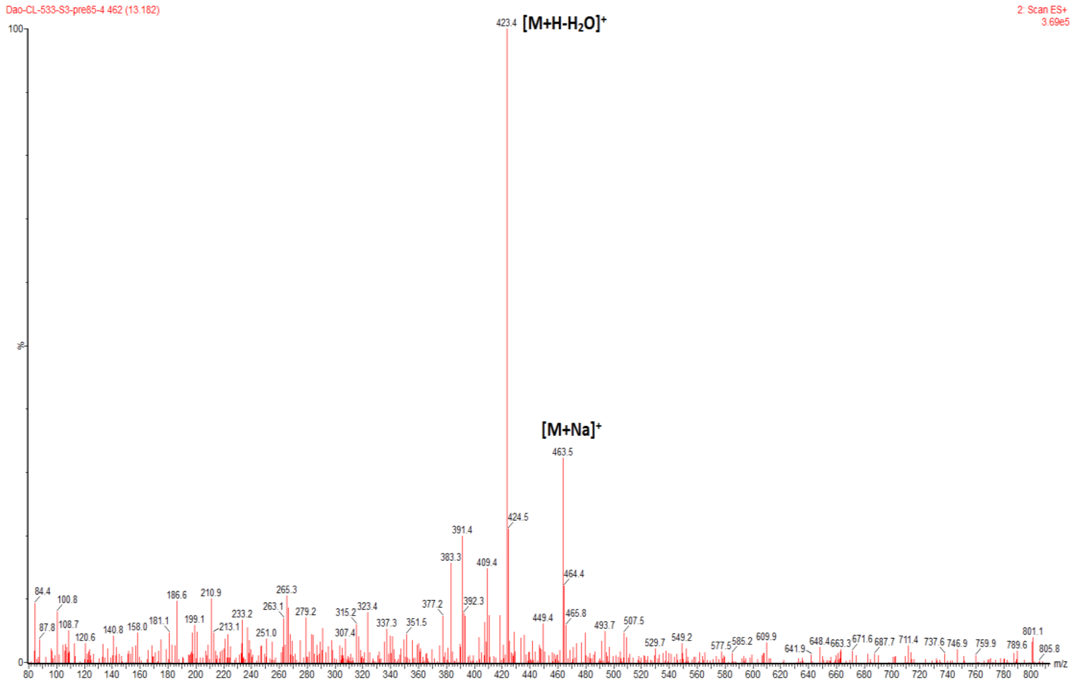

Figure S89: (+)ESI-MS spectrum of **18**.

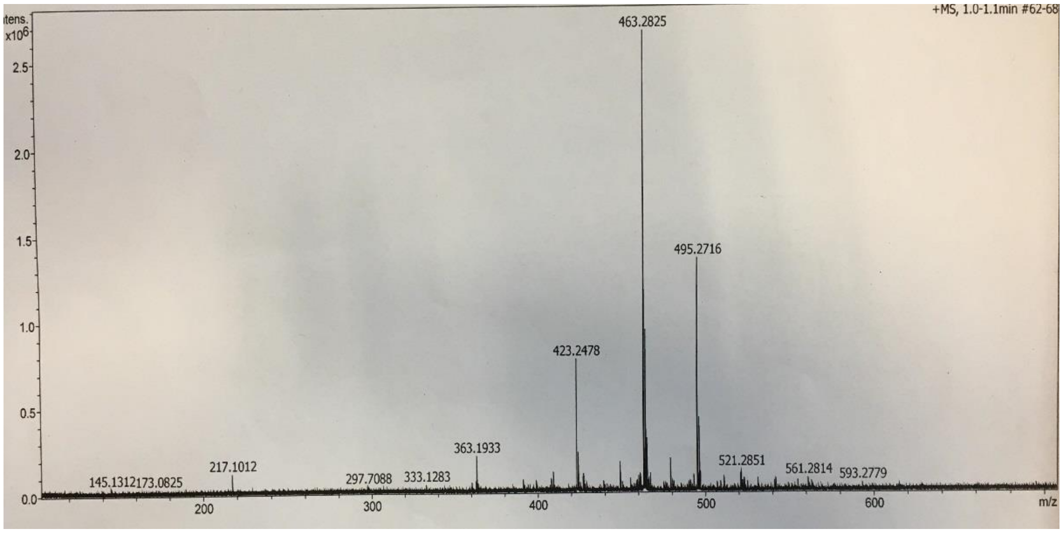

Figure S90: HRESIMS spectrum of **18**.

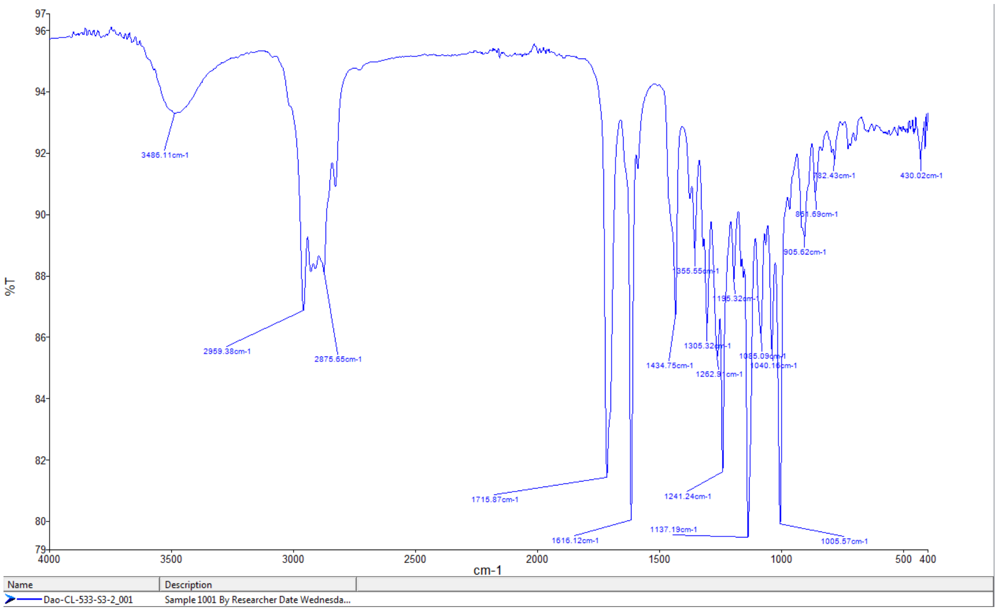

Figure S91: IR spectrum of **18** (film, KBr disc)

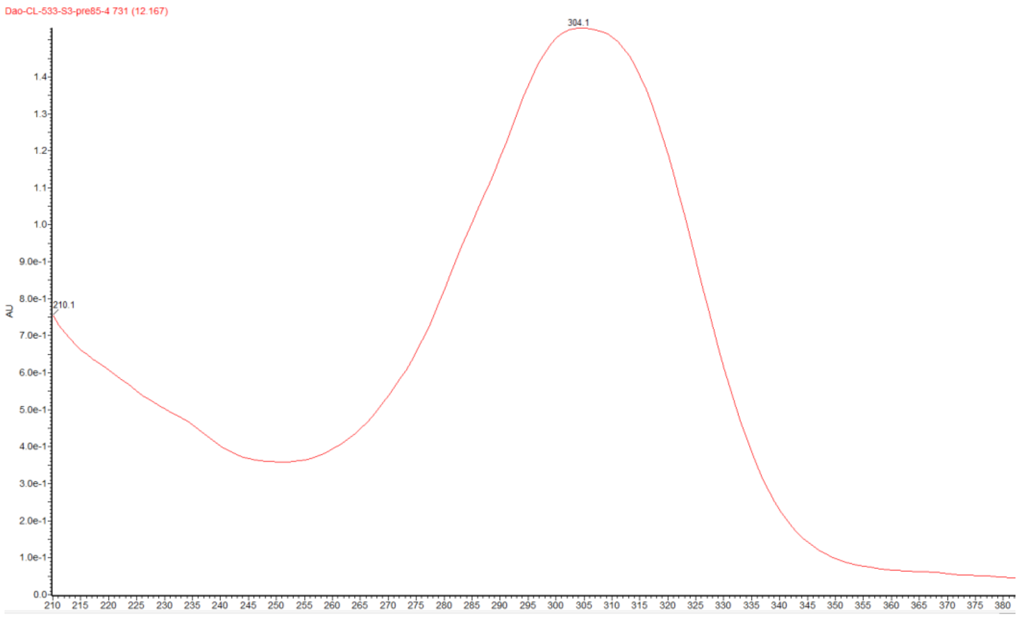

Figure S92: UV/vis (DAD) spectrum of **18** (MeOH).

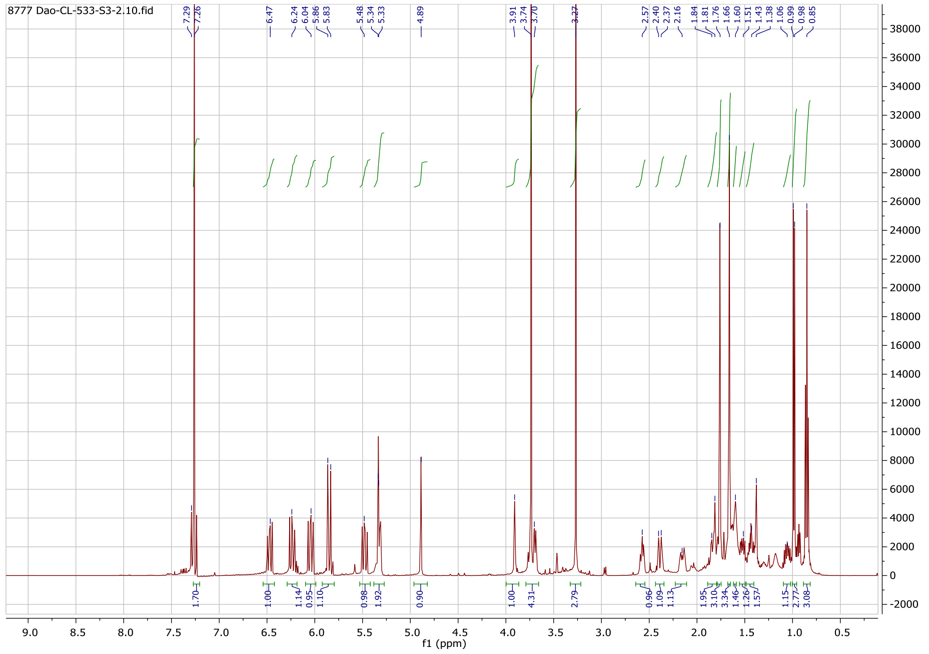

Figure S93: ^{1}H NMR spectrum (500 MHz, $CDCl_3$) of **18**.

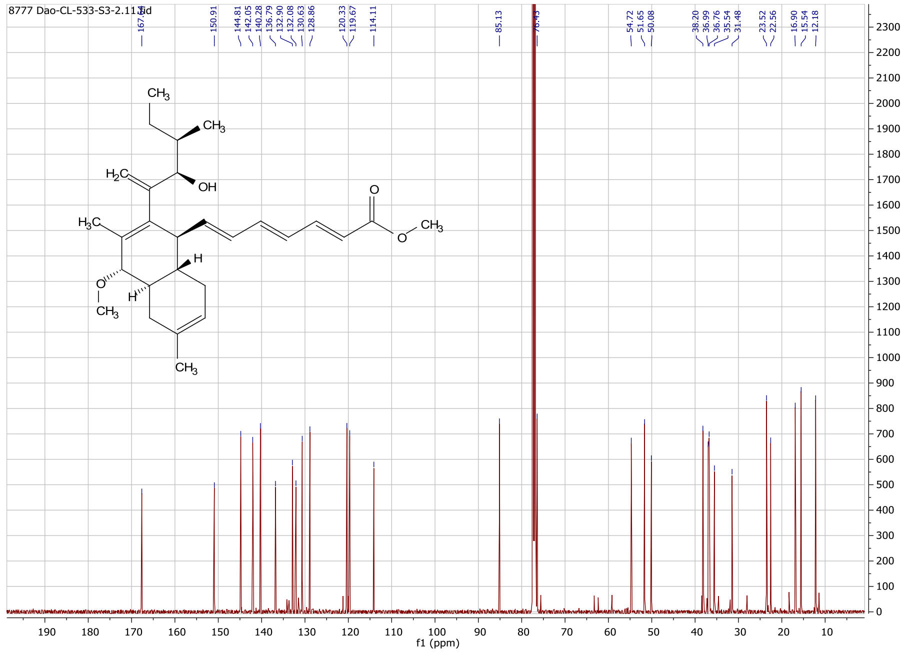

Figure S94: ^{13}C NMR spectrum (125 MHz, $CDCl_3$) of **18**.

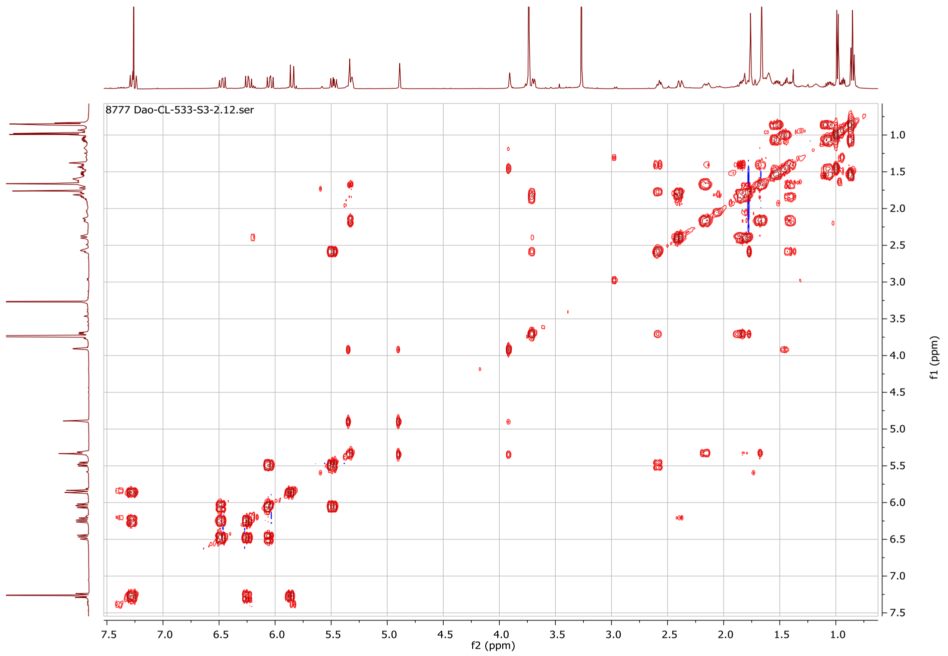

Figure S95: COSY spectrum of **18**.

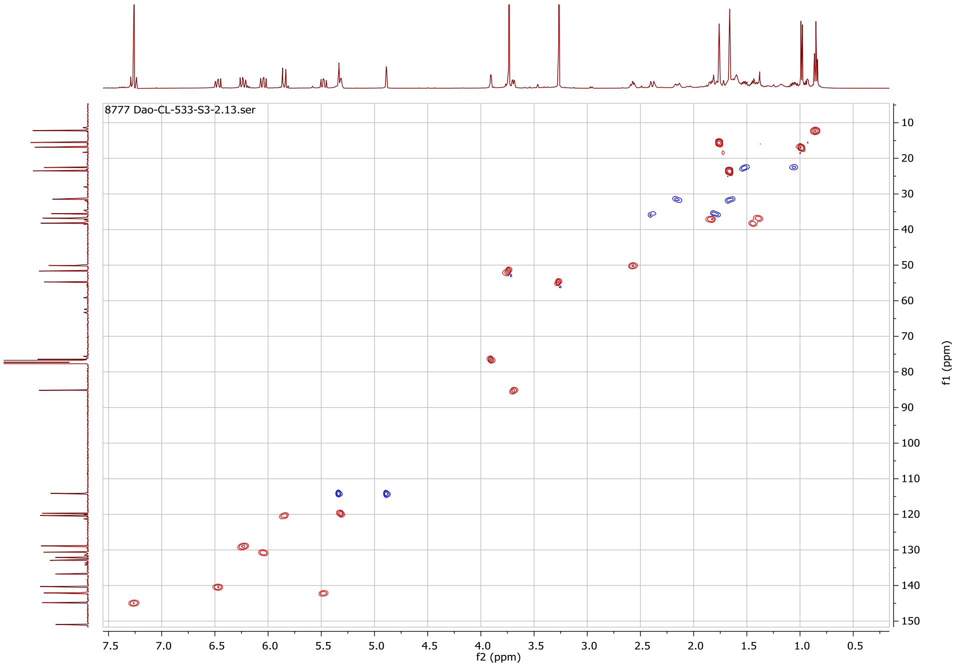

Figure S96: HSQC spectrum of **18**.

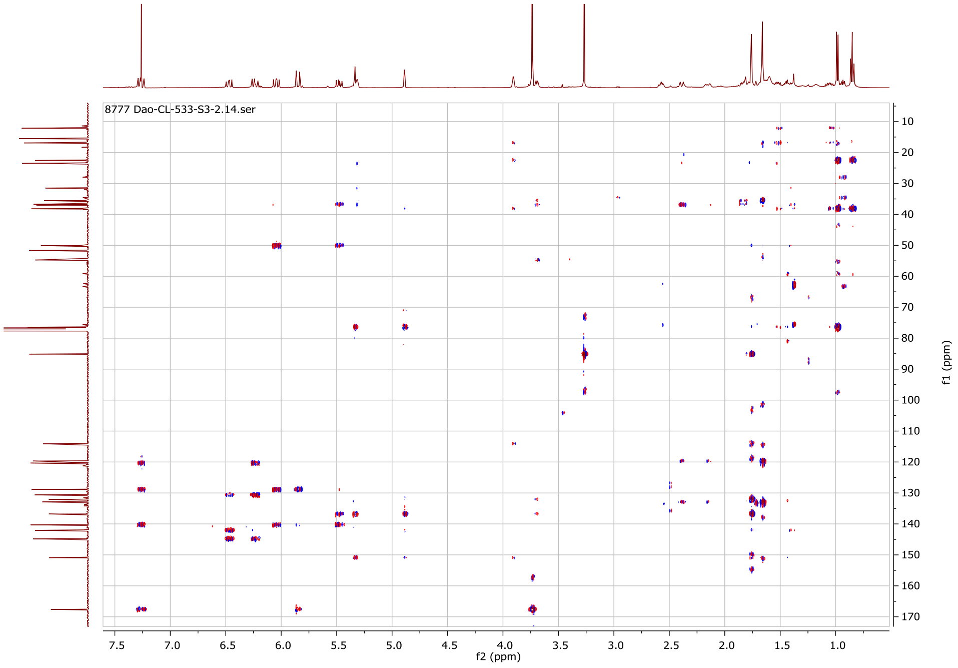

Figure S97: HMBC spectrum of **18**.

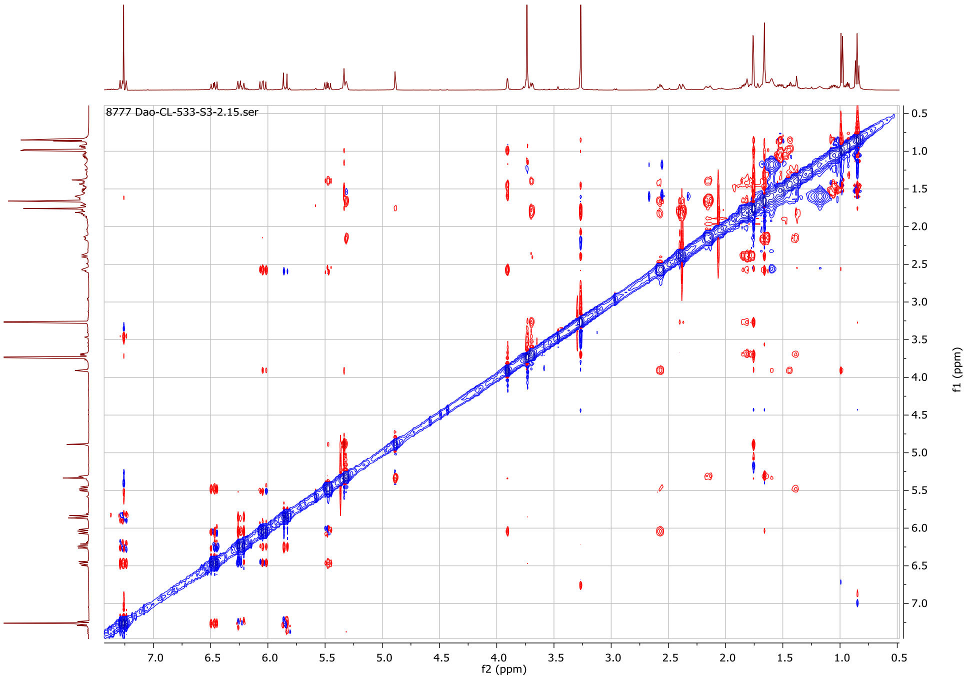

Figure S98: NOESY spectrum **18**.

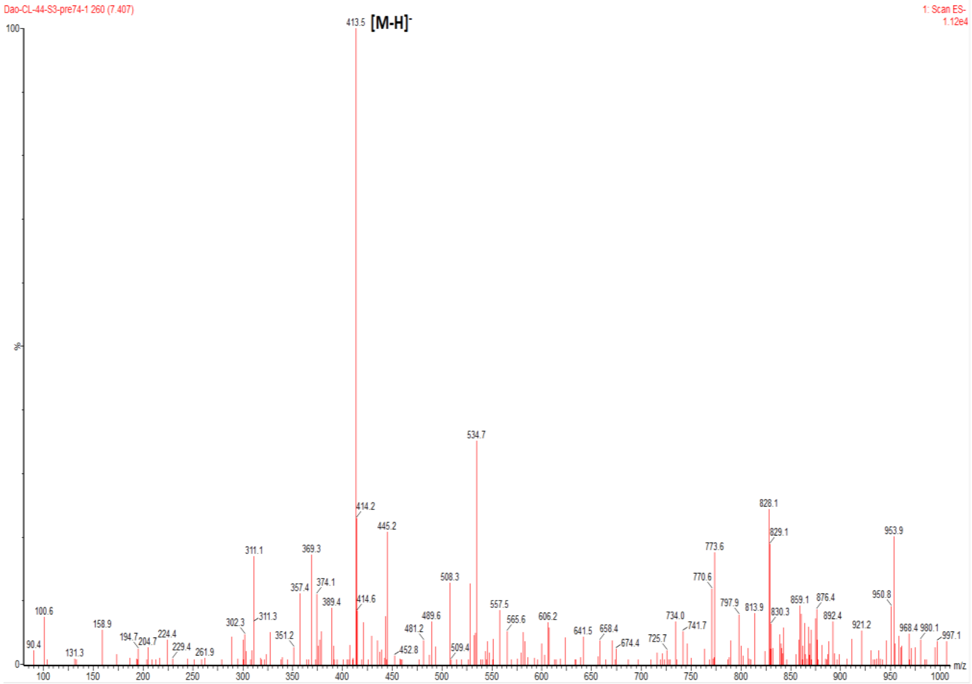

Figure S99: (-)ESI-MS spectrum of **19**.

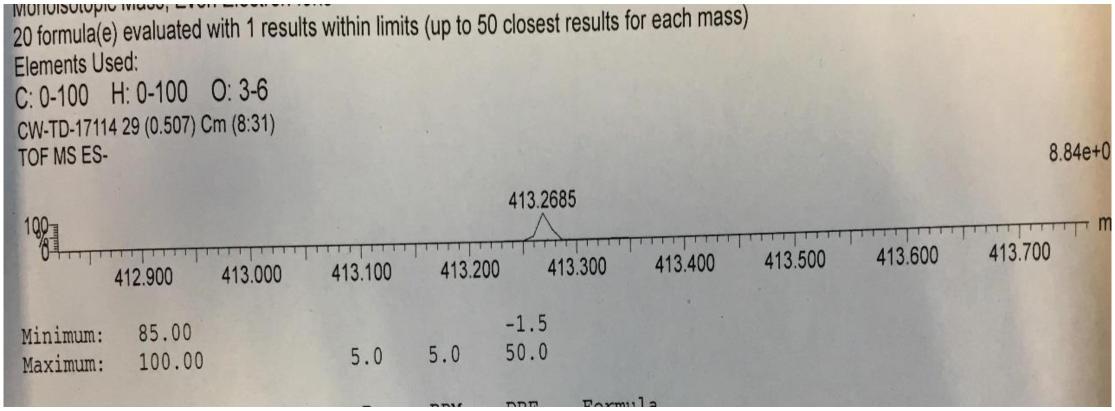

Figure S100: HRESIMS spectrum of **19**.

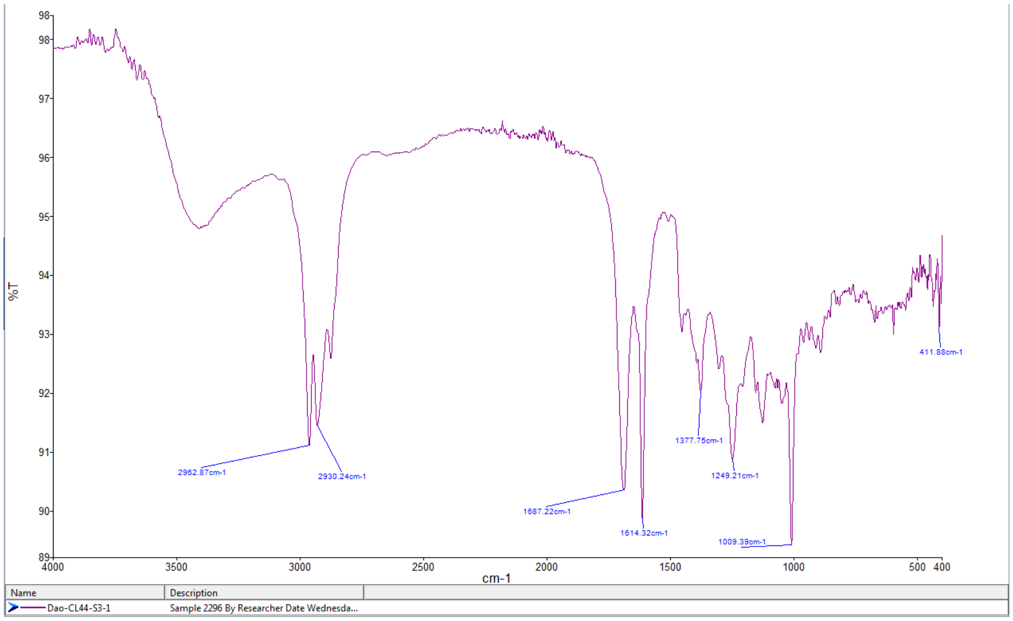

Figure S101: IR spectrum of **19** (film, KBr disc).

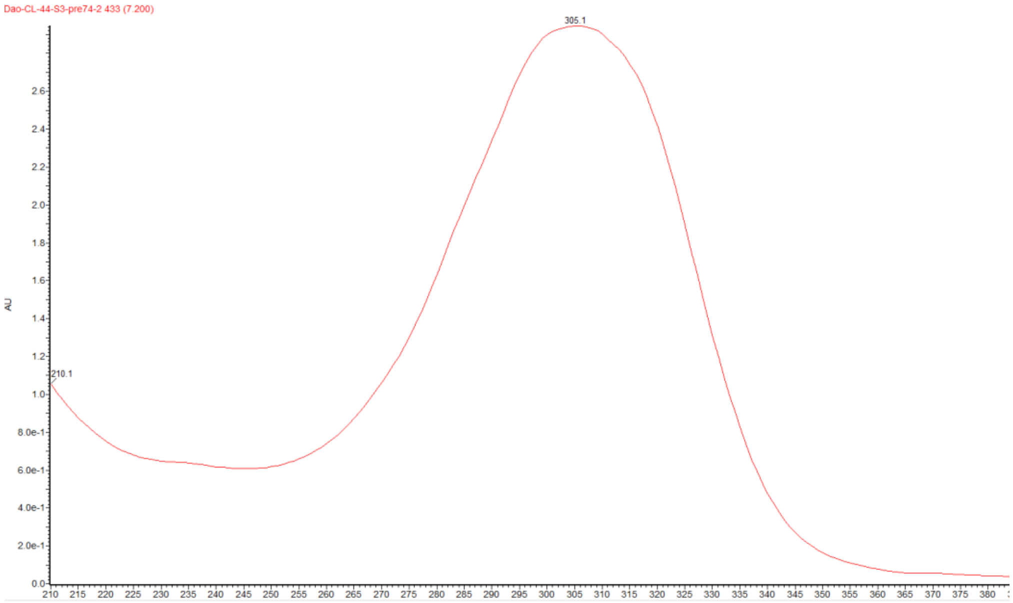

Figure S102: UV/vis (DAD) spectrum of **19** (MeOH).

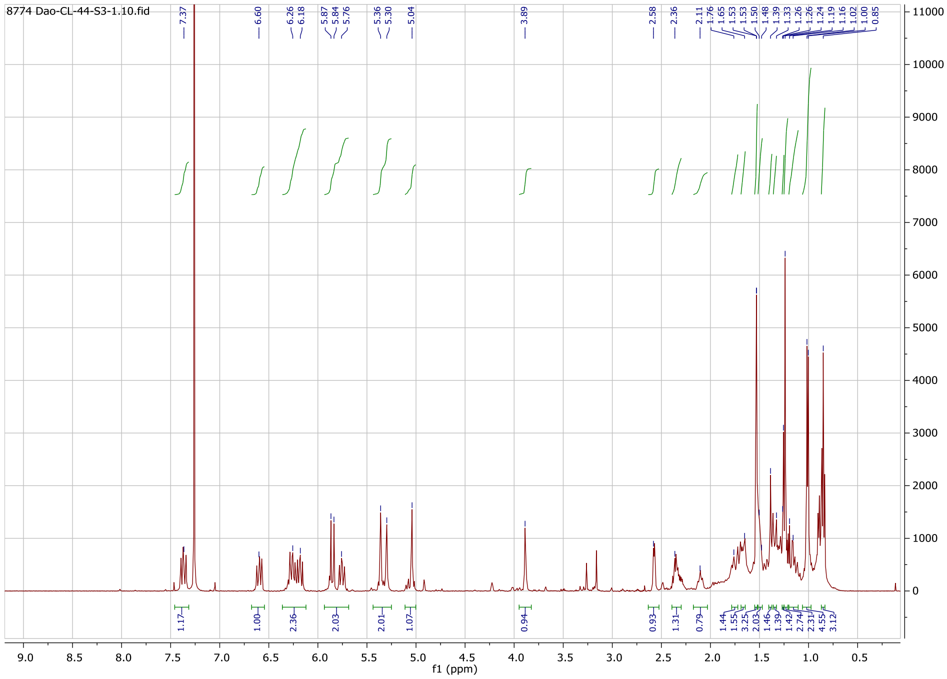

Figure S103: ^{1}H NMR spectrum (500 MHz, $CDCl_3$) of **19**.

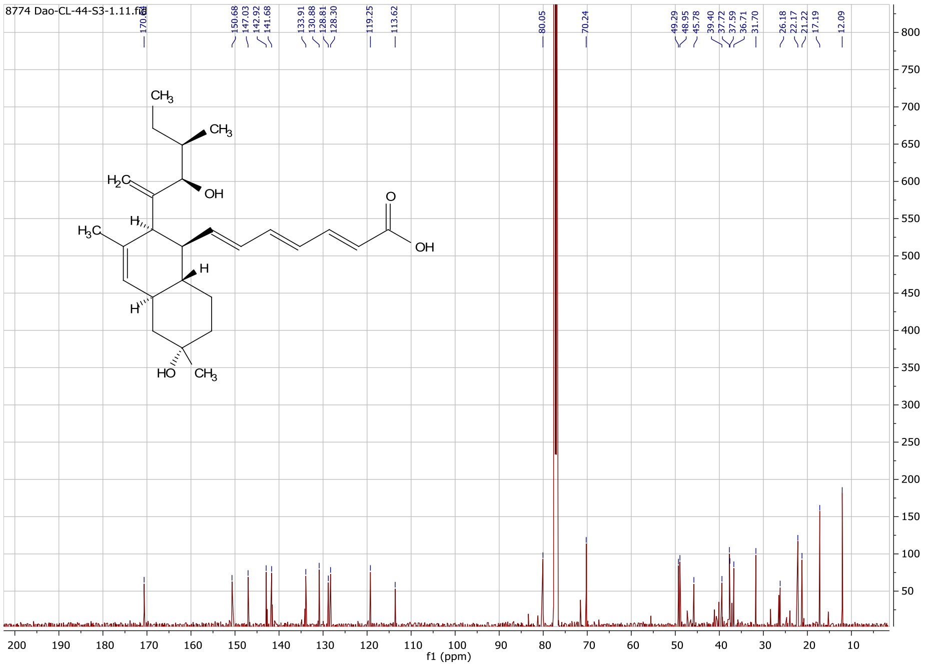

Figure S104: ^{13}C NMR spectrum (125 MHz, $CDCl_3$) of **19**.

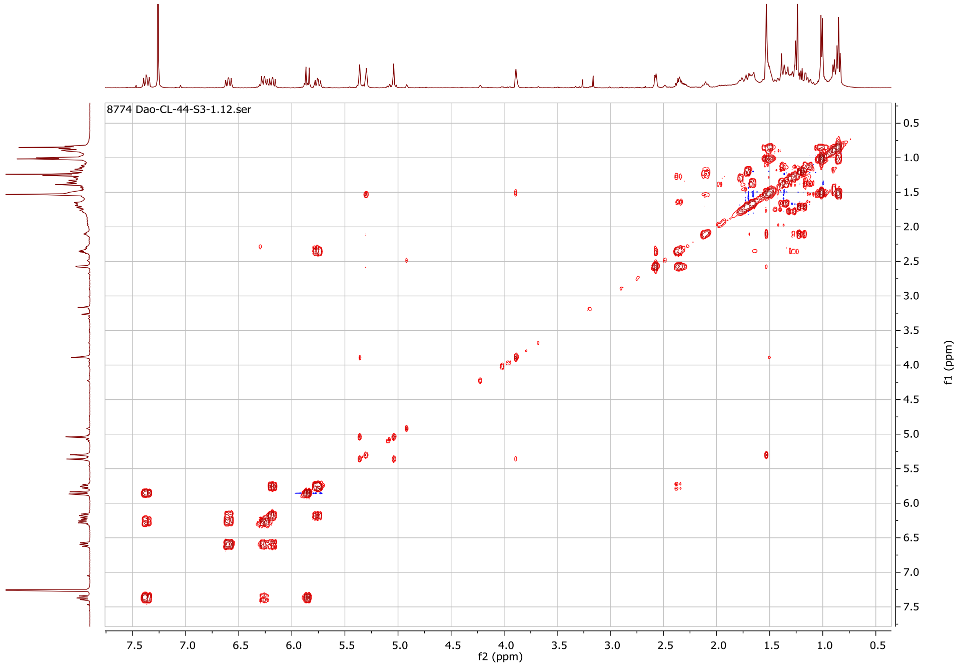

Figure S105: COSY spectrum of **19**.

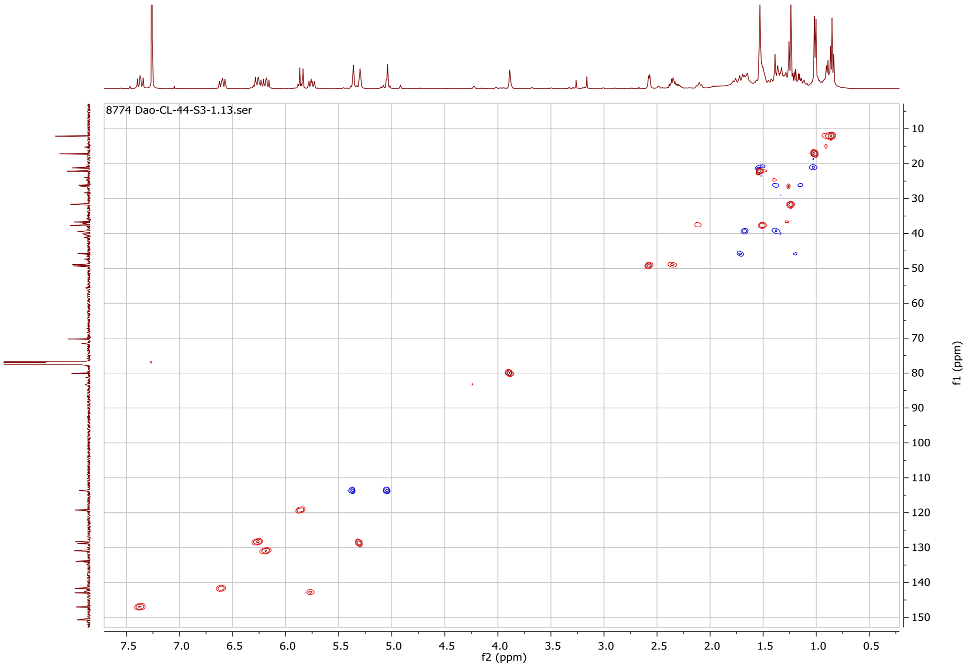

Figure S106: HSQC spectrum of **19**.

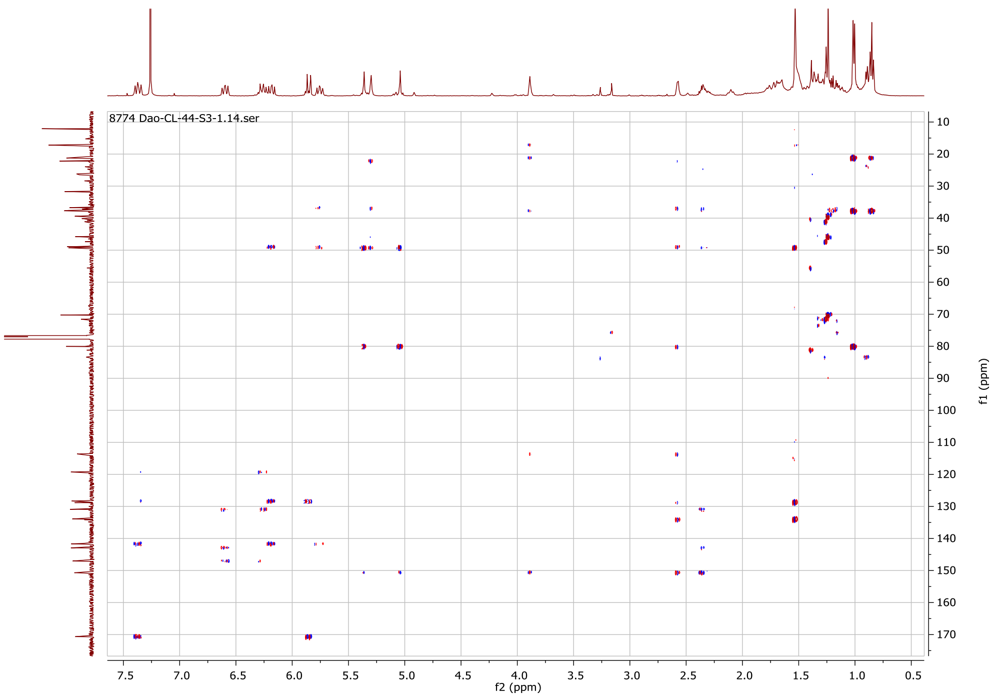

Figure S107: HMBC spectrum of **19**.

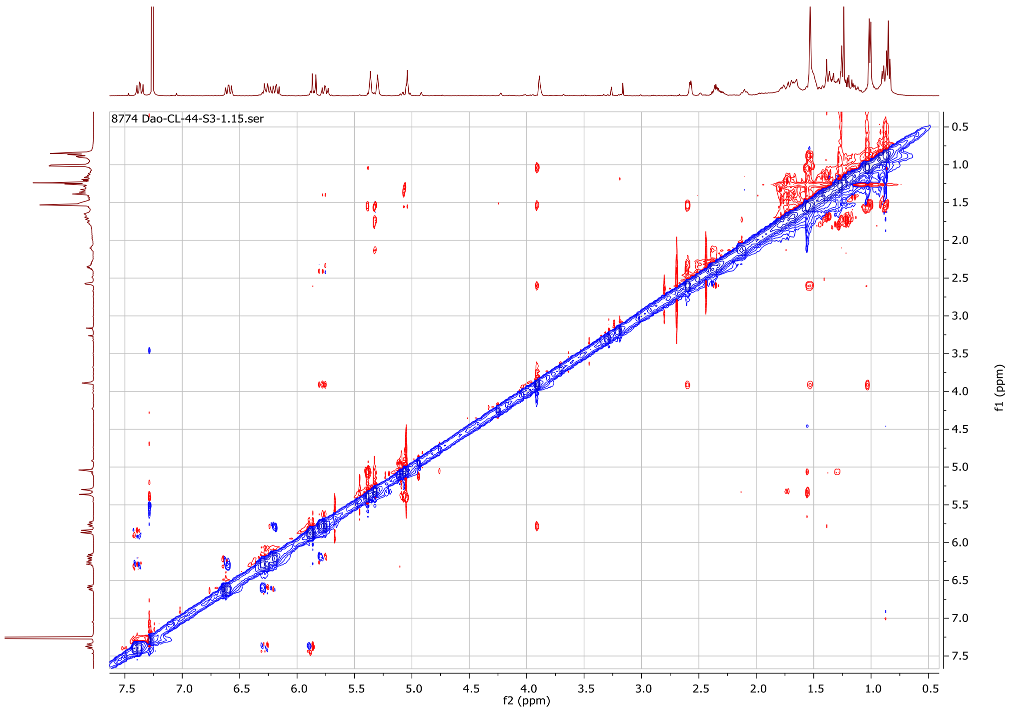

Figure S108: NOESY spectrum **19**.

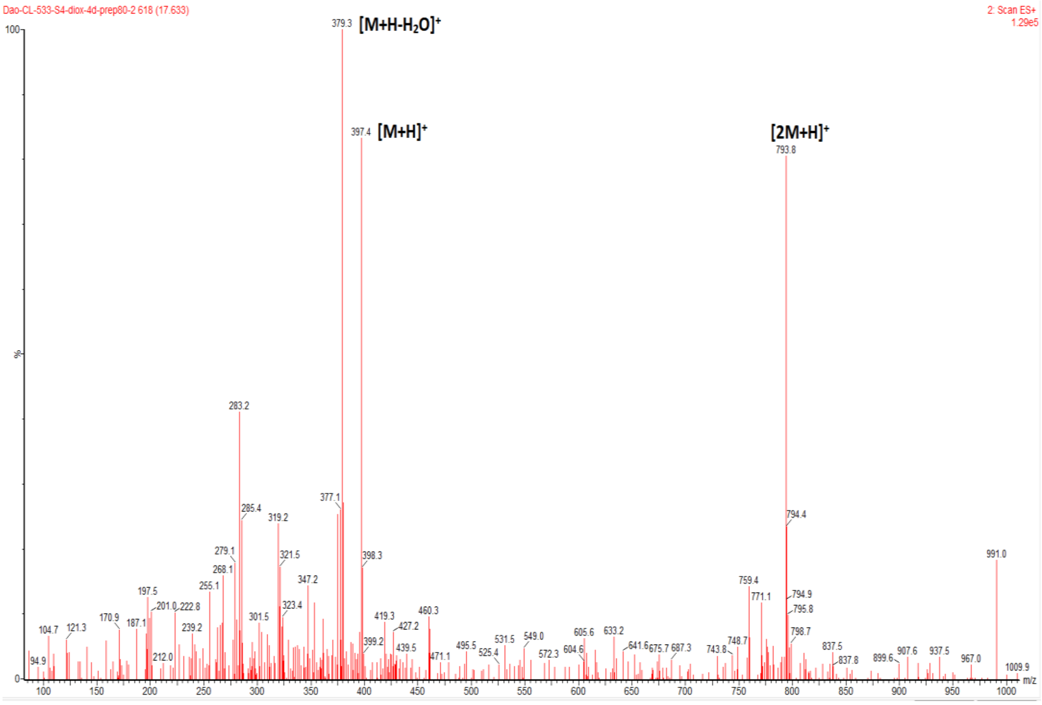

Figure S109: (+)ESI-MS spectrum of **20**.

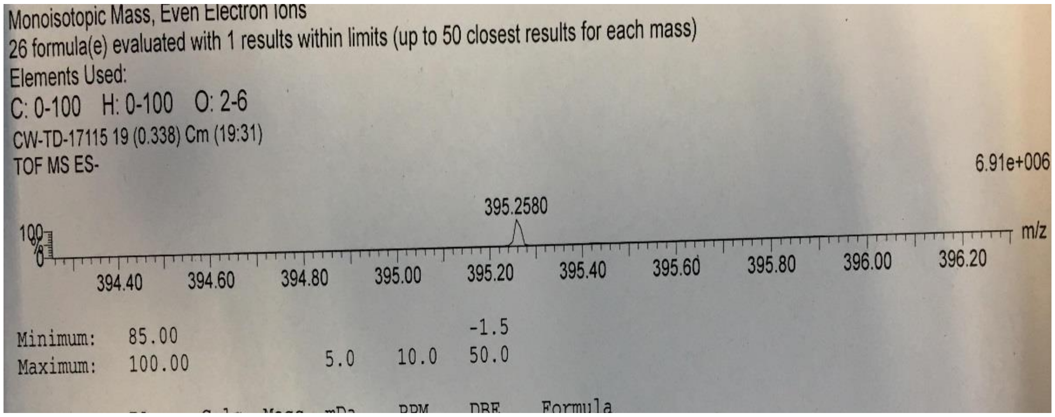

Figure S110: HRESIMS spectrum of **20**.

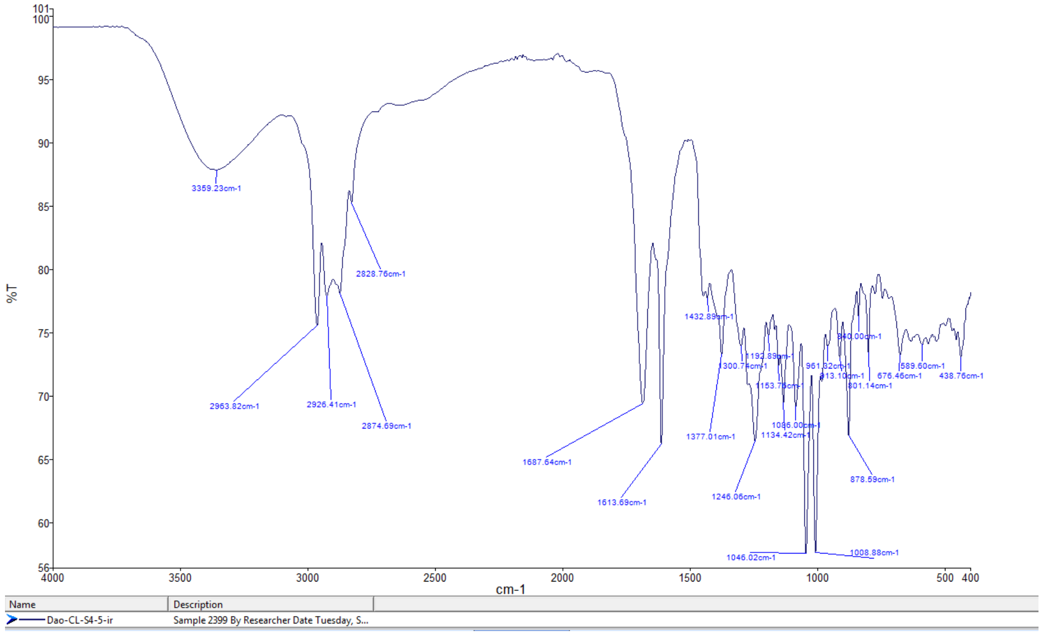

Figure S111: IR spectrum of **20** (film, KBr disc).

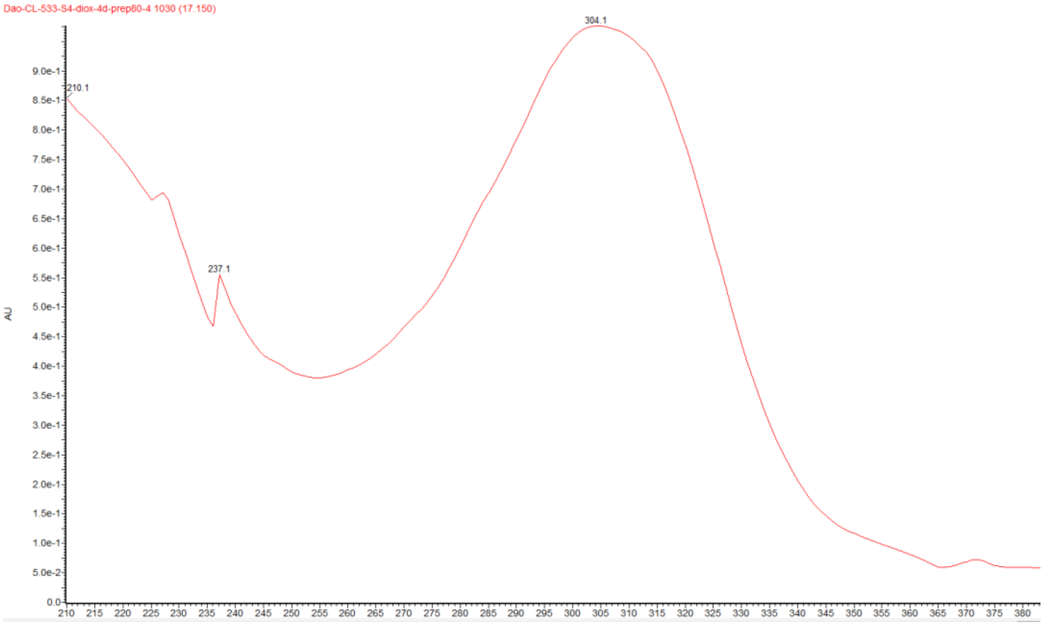

Figure S112: UV/vis (DAD) spectrum of **20** (MeOH).

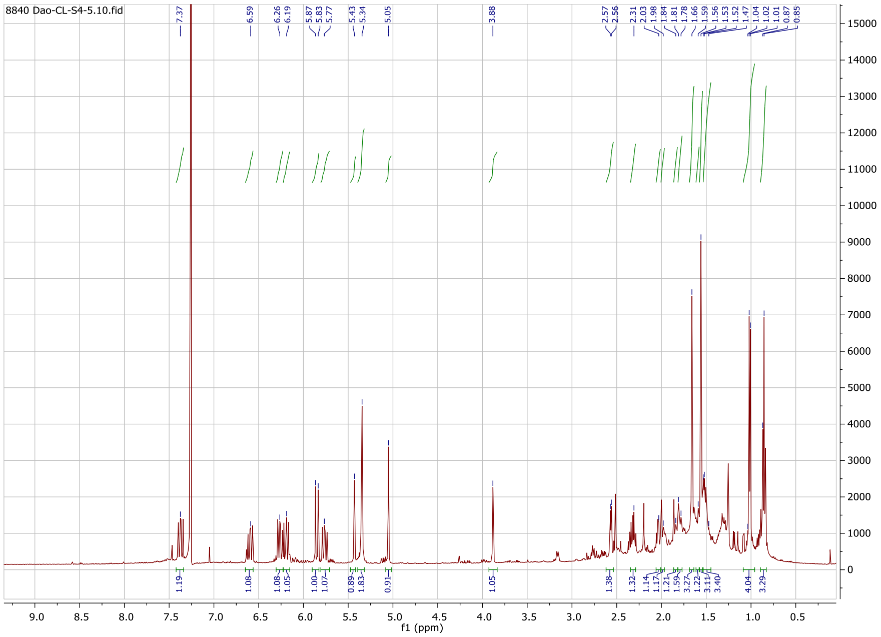

Figure S113: ^{1}H NMR spectrum (500 MHz, $CDCl_3$) of **20**.

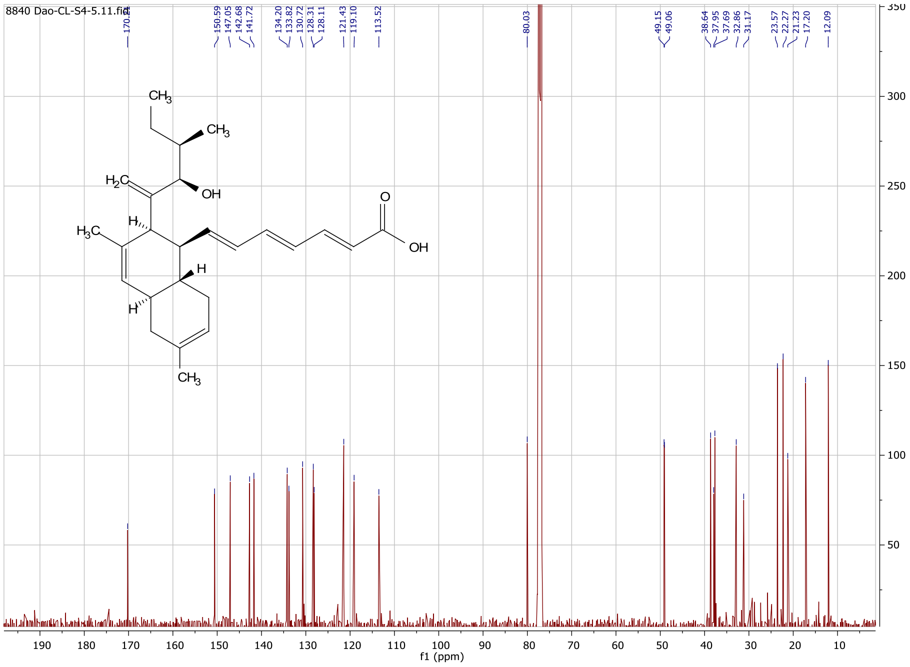

Figure S114: ^{13}C NMR spectrum (125 MHz, $CDCl_3$) of **20**.

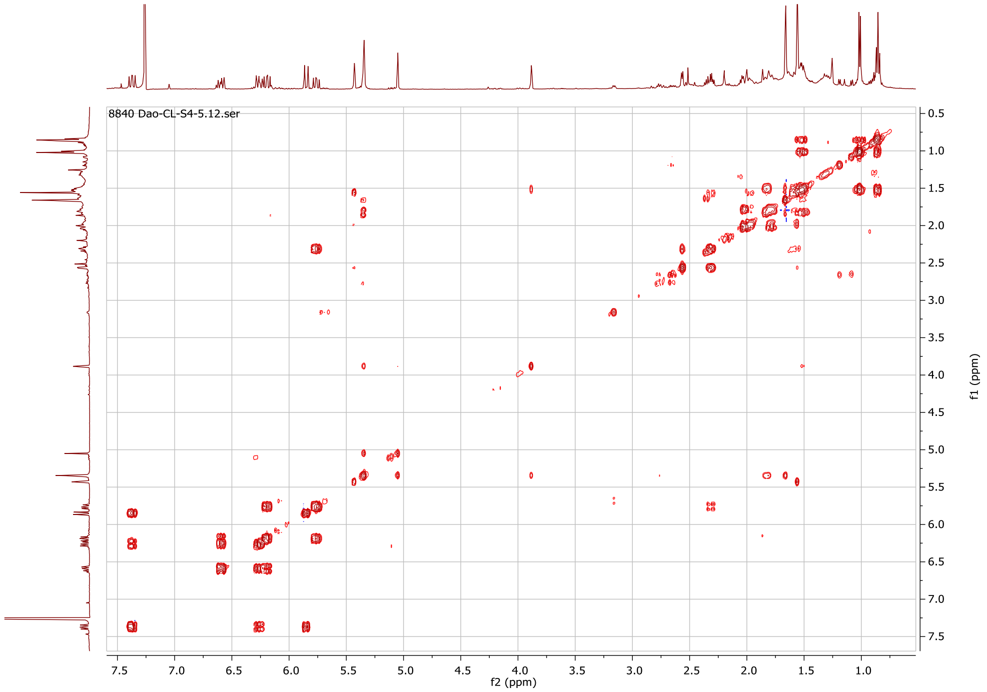

Figure S115: COSY spectrum of **20**.

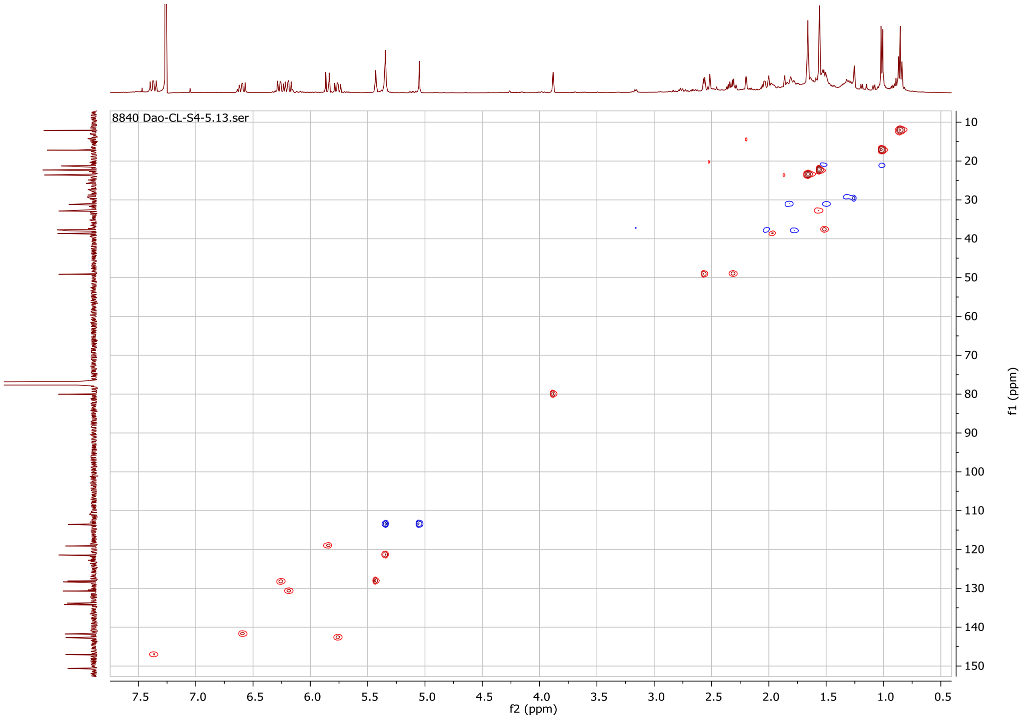

Figure S116: HSQC spectrum of **20**.

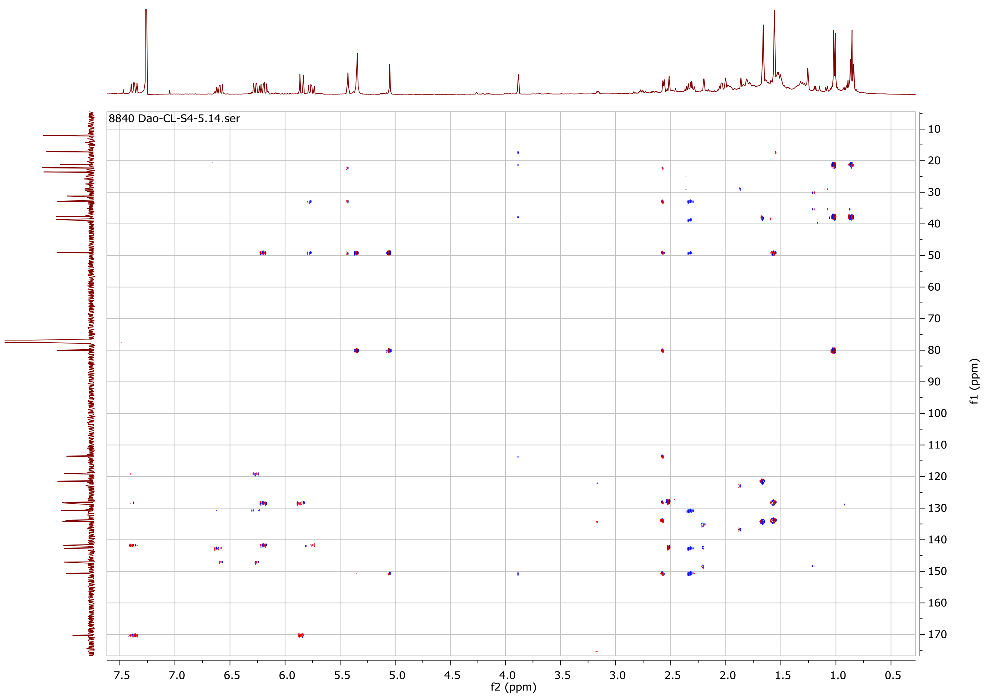

Figure S117: HMBC spectrum of **20**.

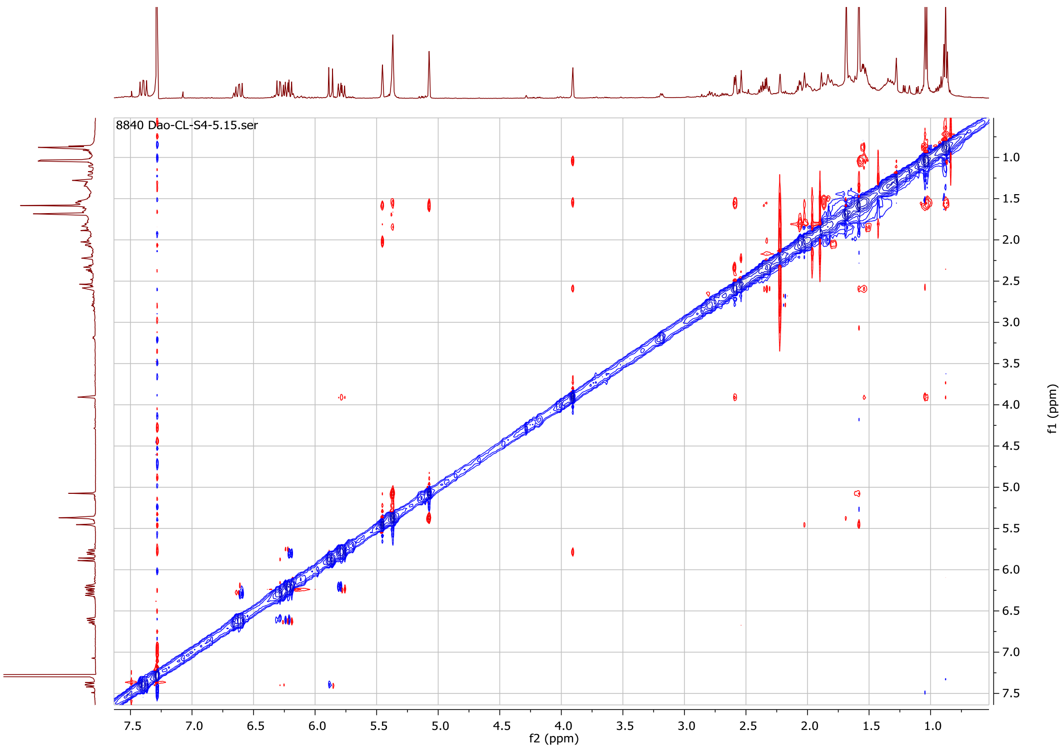

Figure S118: NOESY spectrum **20**.

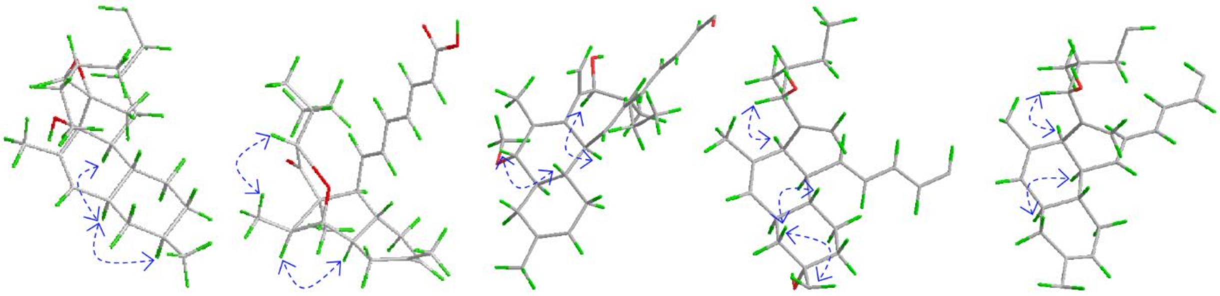

13 17 18 19 20

Figure S119: Key NOESY correlations (indicated by blue arrows) for compounds **13**, **17-20**.

Table S1: The minimum inhibitory concentration (MIC) of tested compounds against Gram-positive bacterium *Bacillus subtilis*.

**Compound**	MIC (μg/mL)	**Compound**	MIC (μg/mL)
**1**	64	**14**	128
**2**	128	**15**	128
**3**	64	**16**	256
**4**	128	**17**	64
**5**	256	**18**	128
**8**	128	**19**	32
**9**	256	**20**	16
**10**	128	Pseudomonic acid A[a]	0.0625
**11**	64	Vancomycin[a]	0.25
**12**	64		
**13**	16		

[a] Positive control.
